# Diretriz Conjunta sobre Tromboembolismo Venoso – 2022

**DOI:** 10.36660/abc.20220213

**Published:** 2022-04-07

**Authors:** Ana Cristina Lopes Albricker, Cláudia Maria Vilas Freire, Simone Nascimento dos Santos, Monica Luiza de Alcantara, Mohamed Hassan Saleh, Armando Luis Cantisano, José Aldo Ribeiro Teodoro, Carmen Lucia Lascasas Porto, Salomon Israel do Amaral, Orlando Carlos Gloria Veloso, Ana Cláudia Gomes Pereira Petisco, Fanilda Souto Barros, Márcio Vinícius Lins de Barros, Adriano José de Souza, Marcone Lima Sobreira, Robson Barbosa de Miranda, Domingos de Moraes, Carlos Gustavo Yuji Verrastro, Alexandre Dias Mançano, Ronaldo de Souza Leão Lima, Valdair Francisco Muglia, Cristina Sebastião Matushita, Rafael Willain Lopes, Artur Martins Novaes Coutinho, Diego Bromfman Pianta, Alair Augusto Sarmet Moreira Damas dos Santos, Bruno de Lima Naves, Marcelo Luiz Campos Vieira, Carlos Eduardo Rochitte

**Affiliations:** 1 Centro Universitário de Belo Horizonte Belo Horizonte MG Brasil Centro Universitário de Belo Horizonte (UniBH), Belo Horizonte , MG – Brasil; 2 Universidade Federal de Minas Gerais Belo Horizonte MG Brasil Universidade Federal de Minas Gerais (UFMG), Belo Horizonte , MG – Brasil; 3 Eccos Diagnóstico Cardiovascular Avançado Brasília DF Brasil Eccos Diagnóstico Cardiovascular Avançado , Brasília , DF – Brasil; 4 Hospital Quinta D’Or Rede D’Or São Luiz Rio de Janeiro RJ Brasil Hospital Quinta D’Or , Rede D’Or São Luiz , Rio de Janeiro , RJ – Brasil; 5 Instituto Dante Pazzanese de Cardiologia São Paulo SP Brasil Instituto Dante Pazzanese de Cardiologia , São Paulo , SP – Brasil; 6 Hospital Barra D’Or Rio de Janeiro RJ Brasil Hospital Barra D’Or , Rio de Janeiro , RJ – Brasil; 7 Ecovitall Rio de Janeiro RJ Brasil Ecovitall , Rio de Janeiro , RJ – Brasil; 8 PRENOTO Clínica Médica e Diagnóstica Ribeirão Preto SP Brasil PRENOTO Clínica Médica e Diagnóstica , Ribeirão Preto , SP – Brasil; 9 Universidade do Estado do Rio de Janeiro Rio de Janeiro RJ Brasil Universidade do Estado do Rio de Janeiro (UERJ), Rio de Janeiro , RJ – Brasil; 10 Hospital Samaritano Rio de Janeiro RJ Brasil Hospital Samaritano , Rio de Janeiro , RJ – Brasil; 11 UnitedHealth Group Rio de Janeiro RJ Brasil UnitedHealth Group (UHG), Rio de Janeiro , RJ – Brasil; 12 Angiolab Vitória Laboratório Vascular Rio de Janeiro RJ Brasil Angiolab Vitória , Laboratório Vascular , Rio de Janeiro , RJ – Brasil; 13 Rede Mater Dei de Saúde, Belo Horizonte MG Brasil Rede Mater Dei de Saúde, Belo Horizonte , MG – Brasil; 14 Ecocenter Medicina Diagnóstica Belo Horizonte MG Brasil Ecocenter Medicina Diagnóstica , Belo Horizonte , MG – Brasil; 15 Hospital das Clínicas Faculdade de Medicina de Botucatu Universidade Estadual Paulista Botucatu SP Brasil Hospital das Clínicas da Faculdade de Medicina de Botucatu (FMB), Universidade Estadual Paulista (UNESP), Botucatu , SP – Brasil; 16 Clínica Fluxo de Cirurgia Vascular São Bernardo do Campo SP Brasil Clínica Fluxo de Cirurgia Vascular , São Bernardo do Campo , SP – Brasil; 17 Universidade Federal do Rio de Janeiro Rio de Janeiro RJ Brasil Universidade Federal do Rio de Janeiro , Rio de Janeiro , RJ – Brasil; 18 Universidade Federal de São Paulo São Paulo SP Brasil Universidade Federal de São Paulo (UNIFESP), São Paulo , SP – Brasil; 19 RA Sabin Medicina Diagnóstica Brasília DF Brasil RA Sabin Medicina Diagnóstica , Brasília , DF – Brasil; 20 Universidade Federal do Rio de Janeiro Rio de Janeiro RJ Brasil Universidade Federal do Rio de Janeiro (UFRJ), Rio de Janeiro , RJ – Brasil; 21 Universidade de São Paulo Ribeirão Preto SP Brasil Universidade de São Paulo (USP), Ribeirão Preto , SP – Brasil; 22 Instituto do Cérebro do Rio Grande do Sul Pontifícia Universidade Católica do Rio Grande do Sul Porto Alegre RS Brasil Instituto do Cérebro do Rio Grande do Sul (InsCer) da Pontifícia Universidade Católica do Rio Grande do Sul (PUCRS), Porto Alegre , RS – Brasil; 23 Medicina Nuclear Joinville São Marcos Diagnósticos Joinville SC Brasil Medicina Nuclear Joinville São Marcos Diagnósticos , Joinville , SC – Brasil; 24 Hospital do Coração São Paulo SP Brasil Hospital do Coração (Hcor), São Paulo , SP – Brasil; 25 Hospital das Clínicas Faculdade de Medicina Universidade de São Paulo São Paulo SP Brasil Hospital das Clínicas da Faculdade de Medicina da Universidade de São Paulo , São Paulo , SP – Brasil; 26 Universidade Federal Fluminense Niterói RJ Brasil Universidade Federal Fluminense (UFF), Niterói , RJ – Brasil; 27 Hospital Madre Teresa Belo Horizonte MG Brasil Hospital Madre Teresa , Belo Horizonte , MG – Brasil; 28 Hospital Israelita Albert Einstein São Paulo SP Brasil Hospital Israelita Albert Einstein , São Paulo , SP – Brasil; 29 Instituto do Coração Faculdade de Medicina Universidade de São Paulo São Paulo SP Brasil Instituto do Coração (InCor) da Faculdade de Medicina da Universidade de São Paulo (FMUSP), São Paulo , SP – Brasil; 30 DASA-ALTA São Paulo SP Brasil DASA-ALTA , São Paulo , SP – Brasil

**Table:** 

Diretriz Conjunta sobre Tromboembolismo Venoso – 2022	
O relatório abaixo lista as declarações de interesse conforme relatadas à SBC pelos especialistas durante o período de desenvolvimento deste posicionamento, 2021/2022.	
Especialista	Tipo de relacionamento com a indústria
Adriano José de Souza	Nada a ser declarado
Alair Augusto Sarmet Moreira Damas dos Santos	Nada a ser declarado
Alexandre Dias Mançano	Nada a ser declarado
Ana Cláudia Gomes Pereira Petisco	Nada a ser declarado
Ana Cristina Lopes Albricker	Outros relacionamentos Financiamento de atividades de educação médica continuada, incluindo viagens, hospedagens e inscrições para congressos e cursos, provenientes da indústria farmacêutica, de órteses, próteses, equipamentos e implantes, brasileiras ou estrangeiras: - Bayer: curso sobre risco cardiovascular no INCOR.
Armando Luis Cantisano	Nada a ser declarado
Artur Martins Novaes Coutinho	Nada a ser declarado
Bruno de Lima Naves	Declaração financeira A - Pagamento de qualquer espécie e desde que economicamente apreciáveis, feitos a (i) você, (ii) ao seu cônjuge/ companheiro ou a qualquer outro membro que resida com você, (iii) a qualquer pessoa jurídica em que qualquer destes seja controlador, sócio, acionista ou participante, de forma direta ou indireta, recebimento por palestras, aulas, atuação como proctor de treinamentos, remunerações, honorários pagos por participações em conselhos consultivos, de investigadores, ou outros comitês, etc. Provenientes da indústria farmacêutica, de órteses, próteses, equipamentos e implantes, brasileiras ou estrangeiras: - Bayer: Xarelto. Outros relacionamentos Financiamento de atividades de educação médica continuada, incluindo viagens, hospedagens e inscrições para congressos e cursos, provenientes da indústria farmacêutica, de órteses, próteses, equipamentos e implantes, brasileiras ou estrangeiras: - Bayer: Xarelto; Apsen: Dobeven; Servier: Daflon.
Carlos Eduardo Rochitte	Nada a ser declarado
Carlos Gustavo Yuji Verrastro	Nada a ser declarado
Carmen Lucia Lascasas Porto	Declaração financeira A - Pagamento de qualquer espécie e desde que economicamente apreciáveis, feitos a (i) você, (ii) ao seu cônjuge/ companheiro ou a qualquer outro membro que resida com você, (iii) a qualquer pessoa jurídica em que qualquer destes seja controlador, sócio, acionista ou participante, de forma direta ou indireta, recebimento por palestras, aulas, atuação como proctor de treinamentos, remunerações, honorários pagos por participações em conselhos consultivos, de investigadores, ou outros comitês, etc. Provenientes da indústria farmacêutica, de órteses, próteses, equipamentos e implantes, brasileiras ou estrangeiras: - Bayer: Xarelto; Pfizer: Eliquis; Boehringer Ingelhem: Pradaxa Estudos fase III. B - Financiamento de pesquisas sob sua responsabilidade direta/pessoal (direcionado ao departamento ou instituição) provenientes da indústria farmacêutica, de órteses, próteses, equipamentos e implantes, brasileiras ou estrangeiras: - Bayer: Xarelto; Pfizer: Eliquis; Boehringer Ingelhem: Pradaxa Estudos fase III. Outros relacionamentos Financiamento de atividades de educação médica continuada, incluindo viagens, hospedagens e inscrições para congressos e cursos, provenientes da indústria farmacêutica, de órteses, próteses, equipamentos e implantes, brasileiras ou estrangeiras: - Bayer: pesquisa.
Cláudia Maria Vilas Freire	Nada a ser declarado
Cristina Sebastião Matushita	Declaração financeira A - Pagamento de qualquer espécie e desde que economicamente apreciáveis, feitos a (i) você, (ii) ao seu cônjuge/ companheiro ou a qualquer outro membro que resida com você, (iii) a qualquer pessoa jurídica em que qualquer destes seja controlador, sócio, acionista ou participante, de forma direta ou indireta, recebimento por palestras, aulas, atuação como proctor de treinamentos, remunerações, honorários pagos por participações em conselhos consultivos, de investigadores, ou outros comitês, etc. Provenientes da indústria farmacêutica, de órteses, próteses, equipamentos e implantes, brasileiras ou estrangeiras: - GE HealthCare: aula PET-CT PSMA e Aplication médica da CZT; R2/IBF: aula PET-CT 18-PSMA/1007; AMB: aula sobre Medicina Nuclear.
Diego Bromfman Pianta	Declaração financeira A - Pagamento de qualquer espécie e desde que economicamente apreciáveis, feitos a (i) você, (ii) ao seu cônjuge/ companheiro ou a qualquer outro membro que resida com você, (iii) a qualquer pessoa jurídica em que qualquer destes seja controlador, sócio, acionista ou participante, de forma direta ou indireta, recebimento por palestras, aulas, atuação como proctor de treinamentos, remunerações, honorários pagos por participações em conselhos consultivos, de investigadores, ou outros comitês, etc. Provenientes da indústria farmacêutica, de órteses, próteses, equipamentos e implantes, brasileiras ou estrangeiras: - GE Healthcare: aula PET-CT PSMA em câncer de próstata; R2/IBF: aula PET-CT 18F-PSMA em câncer de próstata.
Domingos de Morais	Nada a ser declarado
Fanilda Souto Barros	Nada a ser declarado
José Aldo Ribeiro Teodoro	Nada a ser declarado
Marcelo Luiz Campos Vieira	Nada a ser declarado
Márcio Vinícius Lins de Barros	Nada a ser declarado
Marcone Lima Sobreira	Declaração financeira A - Pagamento de qualquer espécie e desde que economicamente apreciáveis, feitos a (i) você, (ii) ao seu cônjuge/ companheiro ou a qualquer outro membro que resida com você, (iii) a qualquer pessoa jurídica em que qualquer destes seja controlador, sócio, acionista ou participante, de forma direta ou indireta, recebimento por palestras, aulas, atuação como proctor de treinamentos, remunerações, honorários pagos por participações em conselhos consultivos, de investigadores, ou outros comitês, etc. Provenientes da indústria farmacêutica, de órteses, próteses, equipamentos e implantes, brasileiras ou estrangeiras: - Sanofi:Enoxaparina e TEV; Bayer: Rivaroxabana e TEV; Pfizer: Apixabana e TEV. B - Financiamento de pesquisas sob sua responsabilidade direta/pessoal (direcionado ao departamento ou instituição) provenientes da indústria farmacêutica, de órteses, próteses, equipamentos e implantes, brasileiras ou estrangeiras: - Pfizer: Apixaban e Doença arterial periférica; Venosan: Ready Wrap é doença venosa crônica; Bayer: Rivaroxabana e TEV. Outros relacionamentos Financiamento de atividades de educação médica continuada, incluindo viagens, hospedagens e inscrições para congressos e cursos, provenientes da indústria farmacêutica, de órteses, próteses, equipamentos e implantes, brasileiras ou estrangeiras: - Sanofi: TEV.
Mohamed Hassan Saleh	Nada a ser declarado
Monica Luiza de Alcantara	Nada a ser declarado
Orlando Carlos Gloria Veloso	Nada a ser declarado
Rafael Willain Lopes	Nada a ser declarado
Robson Barbosa de Miranda	Declaração financeira A - Pagamento de qualquer espécie e desde que economicamente apreciáveis, feitos a (i) você, (ii) ao seu cônjuge/ companheiro ou a qualquer outro membro que resida com você, (iii) a qualquer pessoa jurídica em que qualquer destes seja controlador, sócio, acionista ou participante, de forma direta ou indireta, recebimento por palestras, aulas, atuação como proctor de treinamentos, remunerações, honorários pagos por participações em conselhos consultivos, de investigadores, ou outros comitês, etc. Provenientes da indústria farmacêutica, de órteses, próteses, equipamentos e implantes, brasileiras ou estrangeiras: - Mindray Brasil: Equipamentos de diagnóstico. Outros relacionamentos Participação societária de qualquer natureza e qualquer valor economicamente apreciável de empresas na área de saúde, de ensino ou em empresas concorrentes ou fornecedoras da SBC: - Área de Ensino: Curso de Ecografia Vascular com Doppler.
Ronaldo de Souza Leão Lima	Nada a ser declarado
Salomon Israel do Amaral	Nada a ser declarado
Simone Nascimento dos Santos	Declaração financeira A - Pagamento de qualquer espécie e desde que economicamente apreciáveis, feitos a (i) você, (ii) ao seu cônjuge/ companheiro ou a qualquer outro membro que resida com você, (iii) a qualquer pessoa jurídica em que qualquer destes seja controlador, sócio, acionista ou participante, de forma direta ou indireta, recebimento por palestras, aulas, atuação como proctor de treinamentos, remunerações, honorários pagos por participações em conselhos consultivos, de investigadores, ou outros comitês, etc. Provenientes da indústria farmacêutica, de órteses, próteses, equipamentos e implantes, brasileiras ou estrangeiras: - Jonhson e Abbott: Eco intracardíaco.
Valdair Francisco Muglia	Nada a ser declarado

## Sumário

### Introdução 801

1. Fisiopatologia e Diagnóstico Clínico e Laboratorial do Trombembolismo Venoso 802

1.1. Introdução 802

1.2. Fisiopatologia 802

1.3. Diagnóstico Clínico e Laboratorial 803

**1.3.1. Dosagem do D-dímero** 803

2. Alterações Ecocardiográficas no Trombembolismo Pulmonar 805

2.1. Introdução 805

2.2. Ecocardiograma em Trombembolismo Pulmonar (TEP) de Baixo Risco 805

2.3. Ecocardiograma em Trombembolismo Pulmonar de Alto Risco 807

2.4. Recomendações 813

3. Ultrassonografia Vascular no Diagnóstico da Trombose Venosa Profunda 813

3.1. Sinais Ultrassonográficos da Trombose Venosa: Modo B e *Doppler* 813

**3.1.1. Metodologia de Realização do Exame – Aspectos Técnicos** 813

3.2. Protocolos de Realização de USV na TVP 822

3.3. Protocolos de Exame 823

**3.3.1. Exame Completo de USV** 823

**3.3.2. Exame de Ultrassom de Compressão Completo das Veias dos Membros Inferiores** 826

**3.3.3. Exame de ultrassom de compressão estendido (3 pontos)** 826

**3.3.4. Exame de Ultrassom de Compressão de 2 Pontos** 826

3.4. Diagnóstico Diferencial 827

3.5. Recomendações 827

3.6. Como Descrever o Laudo do Exame 827

4. Ultrassonografia Vascular no Diagnóstico da recorrência de trombose venosa profunda e “síndrome pós-trombótica” 829

4.1. Recorrência de trombose venosa (RTV) 829

4.2. Diagnóstico Ultrassonográfico da Recorrência de Trombose 830

4.3. Síndrome Pós-trombótica 831

**4.3.1. Fisiopatologia** 831

**4.3.2. Quadro Clínico** 831

**4.3.3. Diagnóstico** 831

5. Protocolos de Seguimento com a Ultrassonografia Vascular após a Trombose Venosa Profunda 834

5.1. Introdução 834

5.2. Recanalização 834

5.3. Recorrência da Trombose 834

5.4. Insuficiência Valvular 834

5.5. Discussão 834

**5.5.1. Primeiro USV Negativo** 834

**5.5.2. Trombose Proximal x Distal** 834

**5.5.3. Recorrência de Trombose Venosa Profunda** 836

5.4. Recomendações 836

6. Diagnóstico do Trombembolismo Pulmonar por Angiotomografia, Angio-RM e Angiografia Pulmonar 837

6.1. Angiotomografia de Tórax (Angio-TC) 837

**6.1.1. Técnica e Protocolos de Exames** 837

6.3. Gerações Antigas de Tomógrafos 838

6.4. Critérios Diagnósticos 838

6.5. Critérios Prognósticos 840

6.6. Contraindicação e Situações Especiais 840

6.7. Angiorressonância Magnética (Angio-RM) 841

6.8. Angiografia Digital com Subtração 842

6.9. Recomendações 842

7. Cintilografia Pulmonar 842

7.1. Evidências 842

**7.1.1. Introdução** 842

**7.1.2. Evidências Relacionadas aos Critérios de Interpretação dos Exames** 843

**7.1.3. Acurácia Diagnóstica** 843

**7.1.4. Indicações da Cintilografia Pulmonar por Limitações da Angiotomografia Pulmonar** 845

**7.1.5. Protocolo** 845

**7.1.6. Reconstrução de imagens** 846

**7.1.7. Interpretação** 846

**7.1.8. Trombembolismo Pulmonar Crônico** 846

**7.1.9. Achados Diagnósticos Adicionais** 847

**7.1.10. Pitfalls na Interpretação de Estudos V/Q** 847

**7.1.11. O Futuro da Avaliação de EP por Técnicas de Medicina Nuclear** 847

**7.1.12. Algoritmo Clínico para Investigação de Pacientes com Suspeita de TEP** 848

**7.1.13. Algoritmo Diagnóstico** 849

**7.1.14. Conclusões** 850

Referências 850

## Introdução

O trombembolismo venoso (TEV) manifesta-se como trombembolismo pulmonar (TEP) e/ou trombose venosa profunda (TVP), fazendo parte de um mesmo espectro de doença e apresentando os mesmos fatores de risco. É a terceira causa mais frequente de síndrome cardiovascular aguda no mundo, com potencial risco de vida. ^1 , 2^

No Brasil, segundo dados do Ministério da Saúde coletados entre os anos de 2010 e 2021, o número de internações relacionadas ao TEV ultrapassou 520 mil, com um total de mais de 67.000 óbitos entre 2010 e 2019. ^3^

Apresenta um alto índice de mortalidade, sendo que aproximadamente 34% dos pacientes acometidos morrem subitamente ou em poucas horas após a primeira manifestação, ou seja, antes mesmo de receberem qualquer tipo de tratamento.

Quase 2/3 dos casos de TEV manifestam-se por TVP isolada, sendo a maioria trombose das veias proximais do membro inferior e um terço por TEP. ^2^

Na literatura, diversos estudos relacionam a presença de TEV com a variação climática. No Brasil, Ohki et al. ^4^ relataram maior incidência de TEV nos estados do Sul, onde as temperaturas são mais baixas. A incidência de casos de TEV nesses locais apresentou uma média de 2,86 para cada 100.000 habitantes. ^4^

Tal síndrome clínica aumenta exponencialmente com a idade, mesmo com a aplicação de estratégias de prevenção. ^5^ A mulheres são mais comumente afetadas na juventude, sobretudo no puerpério. Durante a gestação, o uso do tabaco, a trombofilia e/ou a história prévia de TEV aumentam o risco nesse grupo de pacientes. Outras situações, como imobilidade prolongada, obesidade, neoplasias, cirurgias de grande porte com tempo anestésico prolongado, politraumatismos, varizes de membros inferiores, terapia de reposição hormonal e doenças cardiovasculares, são consideradas de risco para TEV, reconhecendo-se que há variação na predição do risco entre essas causas. ^2^

A etnia é considerada um fator de risco para TEV, com uma incidência significativamente maior entre caucasianos e afro-americanos e menor entre hispânicos e asiáticos. De modo geral, aproximadamente 25-50% dos pacientes que apresentam o primeiro episódio de TEV têm condição idiopática, sem um fator de risco facilmente identificável. A mortalidade precoce no TEV está fortemente associada ao acometimento pulmonar, na forma de TEP, além de idade avançada, neoplasias e doença cardiovascular subjacente. ^2^

O índice de recorrência é relevante, e os casos não fatais podem cursar com sequelas como hipertensão pulmonar nos casos de TEP crônico ou síndrome pós-trombótica na TVP. Notadamente, houve uma redução dos óbitos por conta da melhor condução diagnóstica e terapêutica após a publicação de posicionamentos e diretrizes internacionais sobre o tema. ^4^

O diagnóstico de TEV pode não ser tão objetivo; portanto, é necessário foco na abordagem diagnóstica de TEP e TVP, visto que a falha no diagnóstico correto pode ser fatal ou levar a comorbidades permanentes.

Nesse contexto, o Departamento de Imagem Cardiovascular (DIC/SBC), o Colégio Brasileiro de Radiologia (CBR), a Sociedade Brasileira de Angiologia e Cirurgia Vascular (SBACV) e a Sociedade Brasileira de Medicina Nuclear (SBMN) desenvolveram em conjunto este documento. O objetivo é abordar aspectos clínicos e diagnósticos, visando a criar uma recomendação única entre as quatro sociedades, que sirva como fonte de informação para os médicos brasileiros, assim como padronização do diagnóstico clínico, laboratorial e por imagem do TEV. A abordagem profilática e terapêutica do TEV não está no escopo desse documento.

A criação dessa recomendação teve como base diretrizes e estudos populacionais e de caso existentes na literatura, assim como a *expertise* dos participantes. Cada grupo de trabalho compilou os dados, redigiu os escritos e posteriormente apresentou o texto revisado para os ajustes finais. Os temas mais polêmicos foram discutidos em reunião com a participação da maioria. Quando necessário, foram realizadas correções no texto original.

Essa recomendação é direcionada a médicos clínicos ou cirurgiões, visando a melhor informação a respeito da abordagem diagnóstica do TEV, fornecendo dados sobre a acurácia das ferramentas diagnósticas utilizadas na prática diária. Para os médicos imaginologistas, a descrição detalhada dos protocolos de exames poderá auxiliar na realização de diagnósticos mais precisos, contribuindo para a melhor condução clínica dos casos suspeitos ou confirmados.

## 1. Fisiopatologia e Diagnóstico Clínico e Laboratorial do Trombembolismo Venoso

### 1.1. Introdução

O espectro do trombembolismo venoso (TEV) compreende a trombose venosa profunda (TVP) e a embolia pulmonar (TEP). A doença trombembólica é a terceira doença cardiovascular aguda mais comum depois das síndromes isquêmicas cardíacas e do acidente vascular encefálico. A manifestação clínica dessas doenças abrange um amplo espectro, desde clinicamente silenciosa à embolia maciça, levando ao óbito. ^5^ Cerca de um terço de todos os casos de TEP é fatal, e o câncer mostra-se um dos muitos estados de doença associados a um maior risco de doença trombembólica. Aproximadamente dois terços dos casos de TEV são constituídos por TVP, 85-90% em membros inferiores e um terço por TEP. ^6 , 7^ Nesse contexto, devemos sempre lembrar que o TEV é doença grave e passível de prevenção.

### 1.2. Fisiopatologia

#### A) Trombose Venosa Profunda (TVP):

A TVP é uma afecção frequente e potencialmente fatal, tendo como principal complicação a embolia pulmonar, que pode ser considerada a primeira causa de morbimortalidade evitável no ambiente intra-hospitalar. Além disso, também pode levar, como complicação crônica, a síndrome pós-trombótica (SPT), o que acarreta importante impacto socioeconômico. Tais complicações podem ocorrer, apesar do diagnóstico e da terapêutica adequados, tendo seu potencial deletério diminuído quão mais cedo for realizado o diagnóstico e instituído o tratamento correto.

A TVP pode ocorrer tanto em pacientes internados quanto ambulatoriais, tendo sintomatologia inespecífica, podendo variar desde completamente assintomática até um Quadro catastrófico, como na *phlegmasia cerulea dolens* (PCD). A PCD caracteriza-se pela oclusão total ou suboclusão maciça das veias de drenagem do membro (segmento femoroilíaco) e colaterais, podendo se estender à microcirculação. A hipertensão venosa chega a tal nível que impede o fluxo arterial. ^7 , 8^ Com a elevada pressão hidrostática e sequestro de fluidos para o interstício, há formação de edema endurecido de todo o membro, que pode acumular de 3 a 5 litros em volume e levar ao colapso das arteríolas, flictenas pela isquemia tecidual, equimose púrpura, cianose, perda dos pulsos distais, colapso circulatório e choque hipovolêmico. ^9 , 10^

É importante ressaltar que o diagnóstico da TVP tem início com a história clínica. Deve-se atentar para antecedentes que potencializem ou predisponham ao risco aumentado de desenvolver fenômenos trombembólicos venosos, conforme postulado por Virchow:

**- ALTERAÇÕES DO FLUXO (estase):** idade, imobilização ≥3 dias ou inatividade física como em pós-operatório, acidente vascular encefálico, fratura de quadril ou joelho, cirurgia geral de grande porte (>45min, aumentando o risco quanto mais longo for o procedimento), traumatismo grave, lesão da medula espinal com paresia de membro, imobilização de extremidades com aparelhos gessados ou órteses, viagem prolongada em área confinada, gravidez, insuficiência cardíaca congestiva, varizes, DPOC e queimaduras, entre outras. ^11^
**- LESÃO DE ENDOTÉLIO (traumatismo):** idade avançada (a partir dos 40 anos, dobrando o risco a cada década), tabagismo, antecedente trombembólico conhecido, traumatismos, cirurgias e cateteres venosos, entre outras. ^11 , 12^
**- HIPERCOAGULABILIDADE:** neoplasias e/ou seus tratamentos, trombofilias hereditárias ou adquiridas, obesidade, uso de contraceptivos orais contendo estrogênio e reposição hormonal, gravidez, tabagismo, doenças infecciosas agudas, síndrome nefrótica e doença inflamatória intestinal, entre outras. ^9 , 11 , 13 , 14^

## 1.3. Diagnóstico Clínico e Laboratorial

Identificado o risco epidemiológico para TVP, que deve ser obrigatório, proceder ao exame físico do paciente. O diagnóstico clínico da TVP apresenta baixa sensibilidade e especificidade, visto que apenas 20-40% dos pacientes com Quadro clínico sugestivo têm a doença confirmada. Os sinais e sintomas mais comuns apresentados pelos pacientes com TVP são dor e edema. Apesar da baixa acurácia do diagnóstico clínico, é importante ressaltar que, a princípio, todo edema assimétrico em extremidades inferiores deve ser valorizado até a completa avaliação diagnóstica finalizada.

Por outro lado, deve-se levar em conta que em uma parcela considerável dos casos (20-50%) o paciente pode apresentar Quadro de TVP extensa (até mesmo proximal), sem ter qualquer sintomatologia sugestiva. A suspeição clínica é essencial para o diagnóstico nesses casos, mas convém ter por base que o diagnóstico clínico não mostra sensibilidade/especificidade satisfatórios, e sua confirmação deve ser feita por meio de exames complementares. Entre esses métodos, a ultrassonografia vascular com *Doppler* é o método de escolha por sua alta acurácia, sendo de fácil execução, boa reprodutibilidade e inócuo. Entretanto, a disponibilidade dessa modalidade de diagnóstico é limitada em centros médicos menores e ambulatórios e durante os turnos noturnos e de fins de semana. ^15^

A TVP é classificada como proximal quando envolve as veias femorais e/ou poplítea, com ou sem o envolvimento de outras veias da perna; e distal quando envolve as veias profundas infrapatelares. ^16 , 17^ A TVP proximal é a de maior potencial emboligênico. Outras classificações, que podem determinar a gravidade da TVP, são aquelas baseadas no grau de extensão do trombo; os mais extensos costumam gerar consequências maiores por carregarem a hipertensão venosa e o grau de obstrução da luz parcial e total. O grau de ocupação da luz, bem como sua localização e extensão determinará a gravidade e o prognóstico do Quadro clínico da trombose venosa.

No ano de 1997, tentando simplificar a abordagem diagnóstica desses pacientes, Wells et al. ^18^ desenvolveram um modelo de predição clínica para classificar os pacientes quanto ao risco de TVP. O método, quando associado a exames complementares não invasivos, mostrou-se factível e útil em diversos estudos. O mesmo grupo implementou modificações em 2003, tornando mais fácil o entendimento dos clínicos. ^6^ Várias sociedades de especialidade e consensos recomendam a utilização de modelos de predição clínica para estimar a probabilidade do diagnóstico de TVP, antes da realização da USV. ^19^ Entre as ferramentas, a mais utilizada é o escore de Wells associado aos valores séricos do D-dímero. ^7^ ( Quadro 1 ).

**Quadro 1:** – Modelo de predição clínica de trombose venosa profunda (TVP) de Wells 6

Achados Clínicos	Escore
Câncer ativo OU câncer tratado nos últimos seis meses.	1
Paresia, paralisia ou imobilização recente nos MMII.	1
Acamado recente por mais de 3 dias OU cirurgia maior nas últimas 4 semanas.	1
Palpação dolorosa ao longo do trajeto de veias do sistema venoso profundo.	1
Edema de toda a extremidade.	1
Edema de panturrilha com circunferência medindo pelo menos 3cm mais que a circunferência da panturrilha contralateral com medida realizada 10cm abaixo da tuberosidade da tíbia.	1
Edema depressível (cacifo positivo) apenas na perna sintomática.	1
Veias colaterais superficiais não varicosas.	1
TVP prévia documentada.	1
Presença de diagnóstico diferencial mais provável: linfedema, celulite, alterações articulares, tromboflebite superficial, ruptura muscular, cisto de Baker.	-2

- Escore simplificado: ≥ 2 TVP provável; ≤1 TVP improvável
- A variação na acurácia diagnóstica do escore de Wells depende da população avaliada (ambulatorial x internado), da extensão da TVP (proximal x distal) e do grau de probabilidade (baixa x moderada x alta), sendo melhor nos pacientes ambulatoriais com TVP proximal e que apresentam escore de alta probabilidade (>2). De modo geral, Silveira et al., em estudo populacional, obtiveram uma acurácia diagnóstica de 0,56 (AUC – área sobre a curva) para pacientes internados. Em pacientes ambulatoriais, a eficácia do escore pode variar de 11,9% a 79,5%. ^6 , 20 , 21^

### 1.3.1. Dosagem do D-dímero

A medição do D-dímero (um produto de degradação do coágulo de fibrina reticulado) é amplamente utilizada na investigação de pacientes com suspeita de trombembolismo venoso. ^22 , 23^ O ensaio quantitativo do D-dímero, com base no método rápido de Elisa, apresenta alta sensibilidade (próximo de 95%) para o diagnóstico. No entanto, o teste revela baixa especificidade (40%), visto que o D-dímero pode estar aumentado em várias condições além do trombembolismo venoso, como nos casos de infarto agudo do miocárdio, acidente vascular encefálico, inflamações, câncer ativo e gravidez. A especificidade também cai com a idade, e em idosos pode alcançar apenas 10%. Em uma revisão sistemática, apurou-se que o uso de um ponto de corte ajustado para pacientes acima de 50 anos visando a afastar a presença de TVP (idade do paciente em anos [acima de 50] x 10 µg/L) parece tão seguro quanto o ponto de corte padrão, o que foi apontado como uma recomendação na última diretriz europeia de 2019. ^24 , 25^

Consequentemente, um teste quantitativo negativo do D-dímero tem um alto valor preditivo negativo para TEV. Os resultados dos estudos revelam que o risco de desenvolvimento de TEP em pacientes com baixa probabilidade clínica, que não são tratados após um teste de D-dímero negativo, é <1% em três meses após a avaliação inicial. Por outro lado, por conta de seu baixo valor preditivo, um teste D-dímero quantitativo positivo não modifica a probabilidade pré-teste (clínica) e, portanto, é clinicamente inútil. Um teste de D-dímero negativo de alta sensibilidade em combinação com uma baixa probabilidade pré-teste pode excluir a TVP. ^26^

Os níveis do D-dímero também podem variar de acordo com a quantidade de veias acometidas, extensão e volume do trombo. Tromboses proximais e mais extensas apresentam níveis mais altos de D-dímero do que as confinadas às panturrilhas, o que pode ser importante na avaliação da carga da doença trombembólica. ^22 , 27 - 29^ Evidências recentes também sugerem que níveis muito altos do D-dímero estão associados a um aumento de quatro vezes na probabilidade de TEP. ^22 , 30^ Entretanto, quando a probabilidade clínica é alta, independente dos níveis do D-dímero, é obrigatória a realização de um exame de USV para confirmar ou descartar a presença de TVP ^31^ ( Quadro 2 ).

**Quadro 2:**
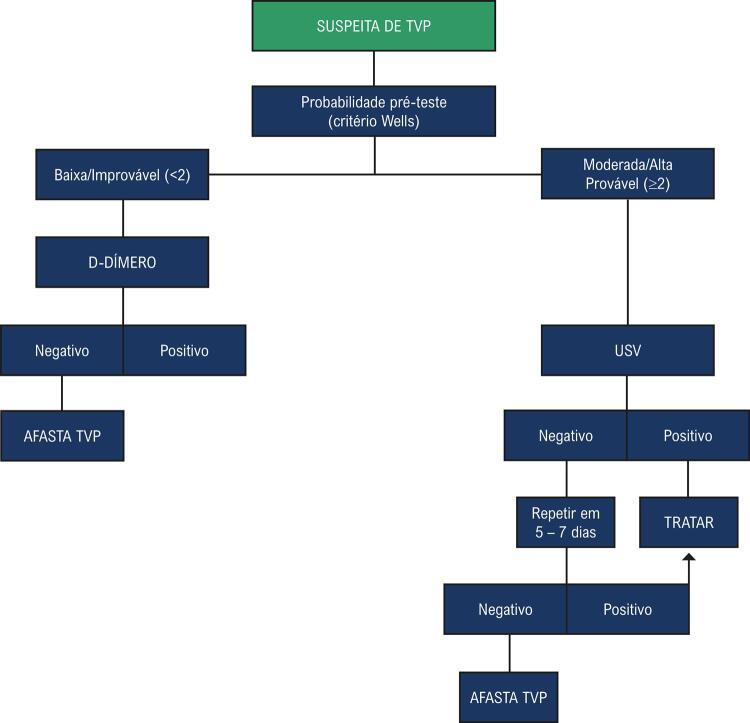
– Fluxograma diagnóstico para a pesquisa de trombose venosa profunda (TVP)

## B) Trombembolismo Pulmonar (TEP):

Define-se o TEP como a obstrução de uma ou mais artérias pulmonares. Na maioria dos casos, é causado por coágulos sanguíneos que chegam às artérias pulmonares vindo, mais comumente, de TVP das extremidades inferiores.

Os sinais clínicos de TEP são inespecíficos, como dispneia, dor torácica, hemoptise, síncope ou pré-síncope. Em alguns casos, pode ser assintomática e descoberta incidentalmente. A síncope parece estar presente em cerca de 17% dos casos e está associada a alta prevalência de instabilidade hemodinâmica e falência do ventrículo direito. O TEP que apresenta instabilidade hemodinâmica não é frequente, mas indica um comprometimento maciço da circulação pulmonar.

Quando suspeito, o TEP deve ser confirmado ou refutado para evitar os riscos de super e subtratamento. Habitualmente, utilizam-se os escores de risco clínico de Wells ou de Genebra para classificar a probabilidade pré-teste.

A acurácia diagnóstica dos escores de Wells para o diagnóstico de TEP foi estabelecida por uma metanálise de 11 estudos, na qual a sensibilidade variou de 63,8% a 79,3%; e a especificidade, de 48,8% a 90%, com área sobre a curva (AUC) de 0,778. Por outro lado, no escore de Genebra a sensibilidade variou de 55,3% a 73,6%; e especificidade, de 48,8% a 90,0%, com área sobre a curva (AUC) de 0,693. ^32^

Tais escores, quando combinados com a dosagem do D-dímero, como descrito anteriormente, apresentam implicação importante na condução diagnóstica e na indicação de testes de imagem ( Quadros 3 e 4 ).

**Quadro 3:** – Modelo de Wells de predição clínica de trombembolismo pulmonar 33

VARIÁVEIS	PONTUAÇÃO SIMPLIFICADA
Sinais clínicos de TVP	3
Frequência cardíaca > 100 bpm	1,5
Imobilização ou cirurgia recente	1,5
TEP ou TVP prévias	1,5
Hemoptise	1
Câncer	1
Diagnóstico alternativo menos provável que TEP	3
TEP provável > 4 e TEP improvável ≤ 4	

**Quadro 4:** – Modelo de Genebra revisado para predição clínica de trombembolismo pulmonar (TEP). Adaptado de Le Gal et al. 34

VARIÁVEIS	PONTOS CLÍNICOS AVALIADOS	
	ORIGINAL	SIMPLIFICADO
TEP ou TVP prévia	3	1
FREQUÊNCIA CARDÍACA		
75 – 94 bpm	3	1
≥ 95 bpm	5	2
Cirurgia ou fratura ≤ 1 mês	2	1
Hemoptise	2	1
Câncer ativo	2	1
Dor unilateral membro inferior	3	1
Dor a palpação no trajeto venoso profundo do membro e edema unilateral	4	1
Idade maior 65 anos	1	1
**PROBABILIDADE CLÍNICA**		
**Escore de 3 níveis**		
Baixo	0 - 3	0 - 1
Intermediário	4 - 10	2 - 4
Alto	≥ 11	≥ 5
**Escore de 2 níveis**		
Improvável	0 - 5	0 - 2
Provável	≥ 6	≥ 3

Na avaliação da probabilidade clínica de TEP, as estratégias diagnósticas dependerão da estabilidade hemodinâmica do paciente. Além do Quadro clínico e do D-dímero, as técnicas complementares recomendadas para diagnóstico de TEP são a angiotomografia pulmonar (angio-TC), a cintilografia com V/Q Spect e o ecocardiograma ( Quadros 5 e 6 ). Cada uma dessas técnicas e o papel do ecocardiograma no fluxograma diagnóstico quando o TEP tem repercussão hemodinâmica, hipotensão ou choque, será discutida nas próximas sessões.

**Quadro 5:**
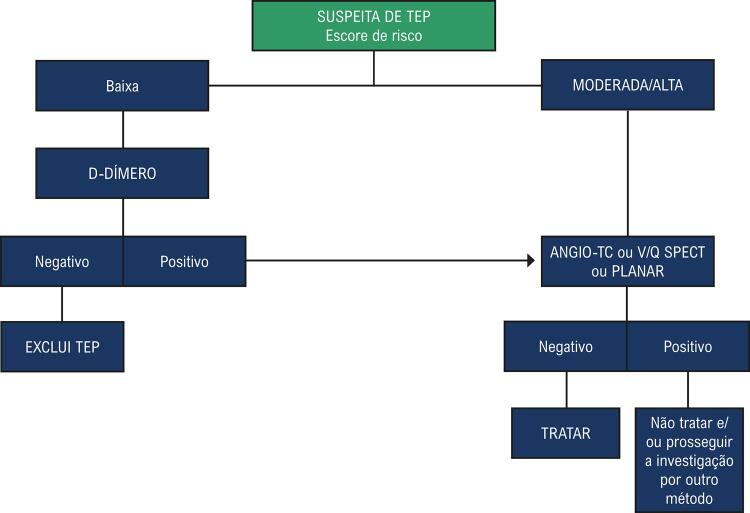
– Fluxograma diagnóstico na avaliação da suspeita de trombembolismo pulmonar (TEP) sem repercussão hemodinâmica

**Quadro 6:**
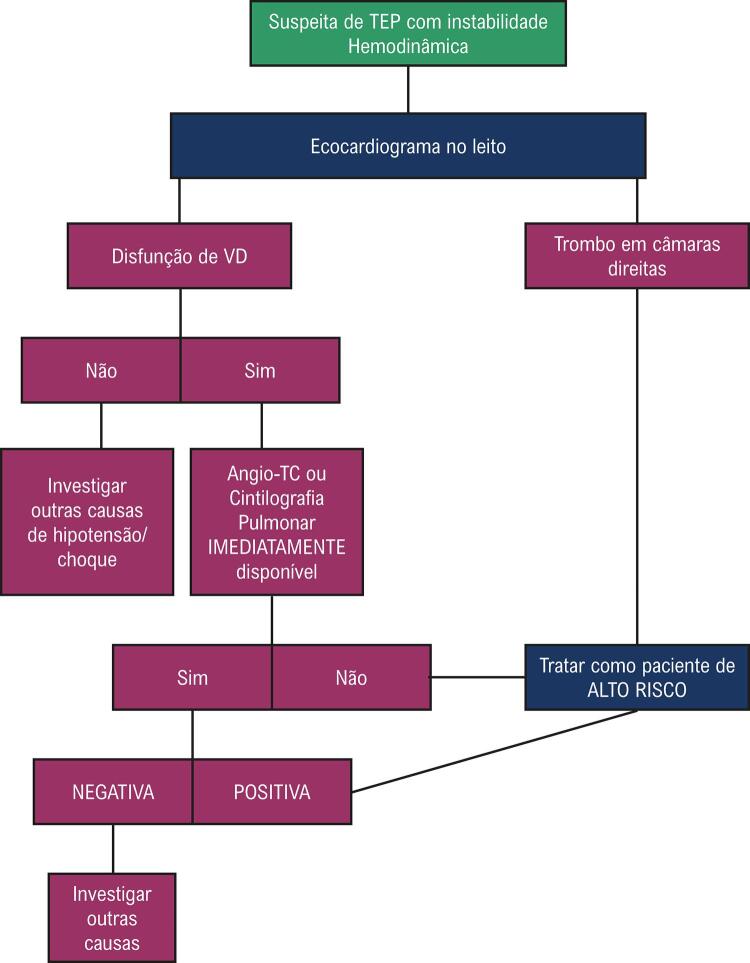
– Algoritmo diagnóstico para trombembolismo pulmonar (TEP) agudo em pacientes com instabilidade hemodinâmica

## 2. Alterações Ecocardiográficas no Trombembolismo Pulmonar

### 2.1. Introdução

A utilidade do ecocardiograma (transtorácico e/ou transesofágico) no trombembolismo pulmonar agudo (TEP) reside na investigação de sobrecarga pressórica de ventrículo direito (decorrente de aumento da resistência vascular pulmonar, com consequente elevação da pós-carga de VD) e na avaliação funcional dessa cavidade. Pacientes com episódio trombembólico sem repercussão hemodinâmica cursam, na maioria das vezes, com exames normais. Em pacientes com instabilidade hemodinâmica, o ecocardiograma desempenha importante papel de apoio nas etapas de diagnóstico e acompanhamento não invasivo da resposta ao tratamento.

## 2.2. Ecocardiograma em Trombembolismo Pulmonar (TEP) de Baixo Risco

O ecocardiograma não é um exame obrigatório na rotina diagnóstica em pacientes com suspeita de TEP com estabilidade hemodinâmica nos algoritmos de investigação. ^1^ O valor preditivo negativo da ecocardiografia varia entre 40-50%; e o resultado normal não exclui TEP. ^35 - 37^ Ainda que não contribua para o diagnóstico, representa importante ferramenta para discriminação prognóstica: a ausência de alterações de tamanho ou função do VD indicam bom prognóstico. ^38 - 41^ O exame é também importante para definir diagnósticos diferenciais de dispneia aguda.

O estudo do ventrículo direito, com seu formato único em crescente, apresenta dificuldade técnica inerente à geometria assimétrica da cavidade. Entretanto, a padronização da técnica para um exame completo foi estabelecida em 2015 em documento conjunto atualizado das sociedades de ecocardiografia norte-americana e europeia. ^42^ As recomendações gerais definem janelas e cortes essenciais que fornecem as imagens exigidas na obtenção de todos os dados necessários para a quantificação:

- Paraesternal esquerda, eixo longo e curto;
- Apical de 4 câmaras;
- Apical de 4 câmaras focando cavidade ventricular direita;
- Paraesternal esquerda demonstrando a via de entrada de VD;
- Subcostal.

O Quadro 7 contém as recomendações para aferir as dimensões cavitárias direitas e os parâmetros de função sistólica ventricular direita.

**Quadro 7:** – Recomendações para medidas de dimensões cavitárias direitas e parâmetros de função sistólica ventricular direita (VD)

MEDIDAS LINEARES DO TRATO DE ENTRADA DO VENTRÍCULO DIREITO (VD)	
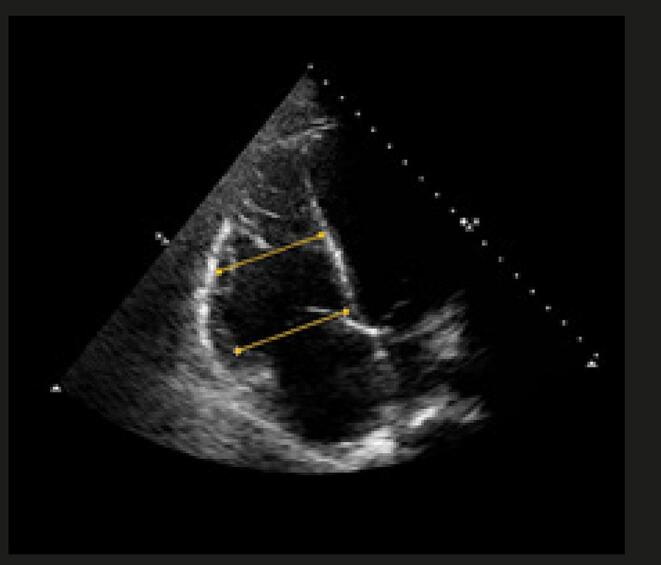	**VDd1 =** diâmetro linear basal do VD (dimensão transversa máxima no 1/3 basal do trato de entrada da cavidade, ao final da diástole na janela focada no VD). VN = 33 ± 4mm ^25-41^ **VDd2 =** diâmetro linear médio cavitário do VD (dimensão transversa do VD no 1/3 médio do trato de entrada, aproximadamente no ponto entre o diâmetro basal máximo e o ápice, na altura dos músculos papilares, ao final da diástole. VN = 27 ± 4mm ^19-35^
**MEDIDAS LINEARES DO TRATO DE SAÍDA DO VENTRÍCULO DIREITO (VD)**	
** 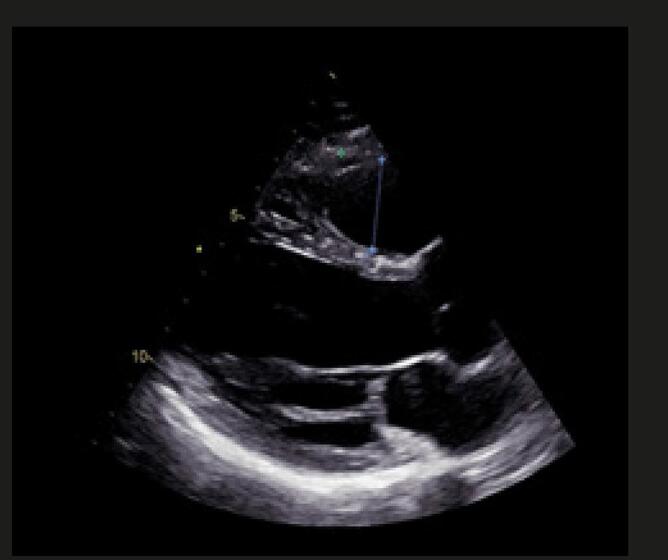 **	**A)** Diâmetro proximal do trato de saída do VD (janela paraesternal longitudinal) = dimensão linear medida da parede anterior do VD até a junção interventricular septo aórtica, ao final da diástole. VN = 25 ± 2,5mm ^20-30^
** 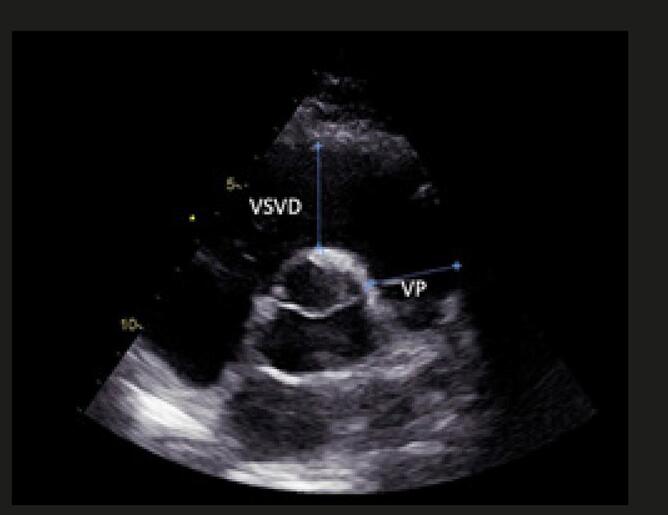 **	**B)** Diâmetro proximal do trato de saída de VD (janela paraesternal, corte transverso) = dimensão linear medida da parede anterior do VD até a válvula aórtica, ao final da diástole. VN = 28 ± 3,5mm ^21-35^
** 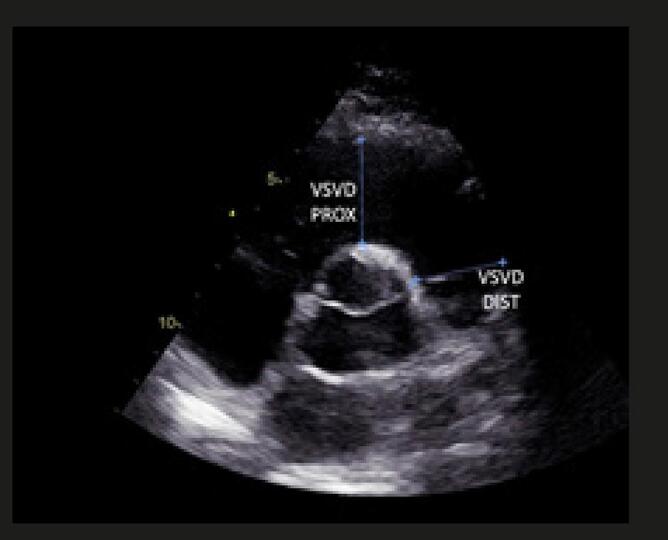 **	**C)** Diâmetro distal do trato de saída do VD (janela paresternal, corte transverso) = dimensão linear transversal medida justa proximal à válvula pulmonar ao final da diástole. VN = 22 ± 2,5mm ^17-27^
**MEDIDAS LINEARES DA ESPESSURA DA PAREDE DO VD**	
** 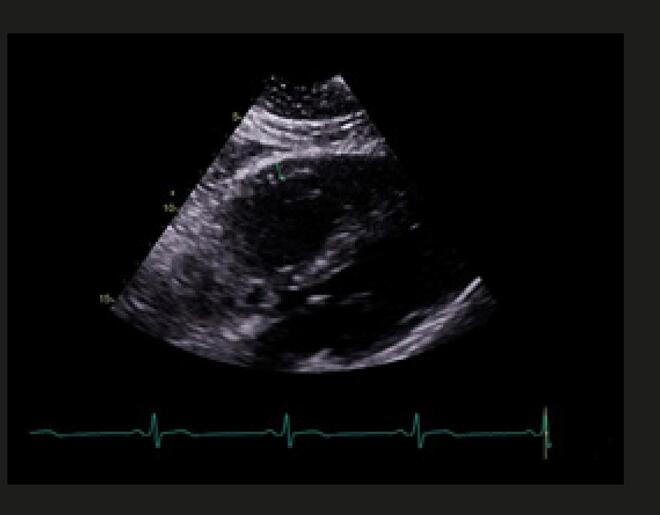 **	A medida linear da espessura de parede do VD (por meio do modo uni ou bidimensional) é realizada ao final da diástole, abaixo do anel tricúspide e em uma distância que se aproxima do comprimento do folheto anterior desta válvula (totalmente aberta e paralela à parede livre do ventrículo direito). VN = 3 ± 1mm ^1-5^
**MEDIDAS VOLUMÉTRICAS DO VD AO ECOCARDIOGRAMA TRIDIMENSIONAL (ECO 3D)**	
** 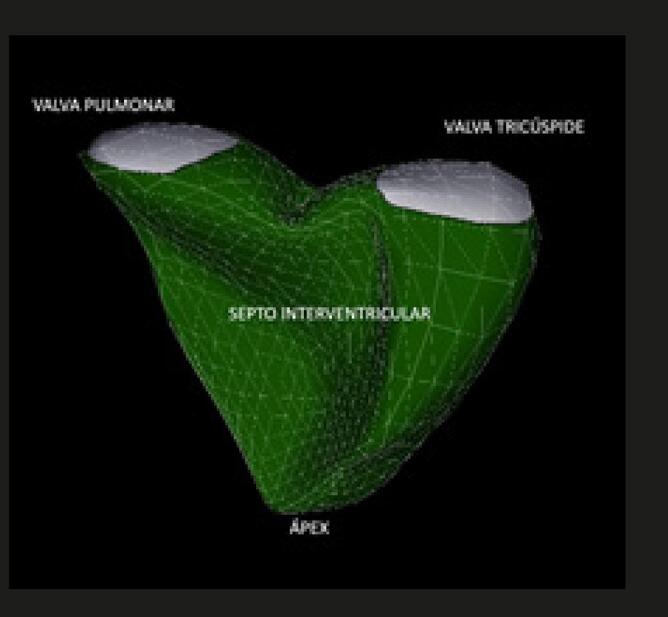 **	Recomenda-se que o tamanho do VD deve ser medido por meio do eco bidimensional convencional, utilizando múltiplas janelas acústicas. Entretanto, nos laboratórios com experiência em eco 3D, é recomendada a quantificação dos volumes através de tal método. VN para VDFVD: Homem 61 ± 13mL/m ^235-87^ Mulher 53 ± 10,5mL/m ^232-74^ VN para VSFVD: Homem 27 ± 8,5mL/m ^210-44^ Mulher 22 ± 7 mL/m ^28-36^
**FUNÇÃO SISTÓLICA GLOBAL DO VD AVALIADA PELA ÁREA DA FRAÇÃO DE ENCURTAMENTO DE VD – (FAC)**	
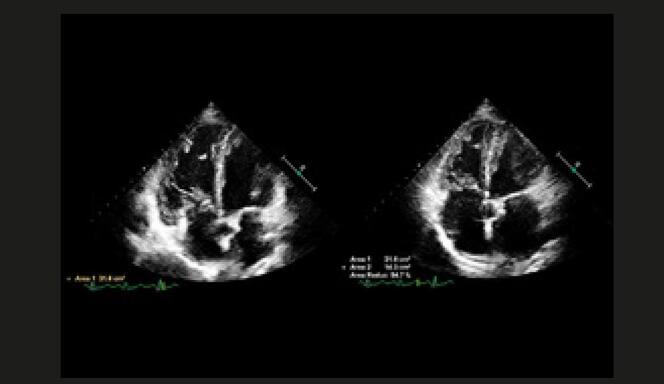	FAC VD (%) = 100 X (ADF – ASF)/ADF Apical de 4 câmaras focado no VD. Traçado manual da borda endocárdica do VD desde o anel tricúspide lateral, passando pela parede livre até o ápice e retornando ao anel tricúspide medial através do septo interventricular. Medida realizada ao final da sístole (ASF) e diástole (ADF). Trabeculações, músculos papilares e banda moderadora são incluídos na medida da área cavitária. VN = 49 ± 7% (>35%)
**FUNÇÃO SISTÓLICA GLOBAL DE VD AVALIADA ATRAVÉS DA FRAÇÃO DE EJEÇÃO PELO ECO 3D**	
** 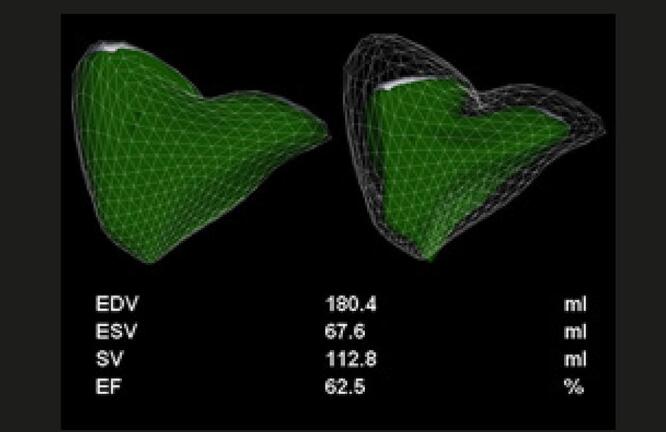 **	Alteração fracional de volume de ventrículo direito pelo eco 3D [FEVD (%) = 100 X (VDF-VSF)/VDF]. Quando realizada corretamente, correlaciona-se com fração de ejeção calculada pela ressonância nuclear magnética. Em laboratórios capacitados, recomenda-se a medida de fração e de ejeção de VD pelo eco 3D. VN = 58 ± 6,5% (>45%)
**FUNÇÃO SISTÓLICA LONGITUDINAL DE VD AVALIADA PELO TAPSE**	
** 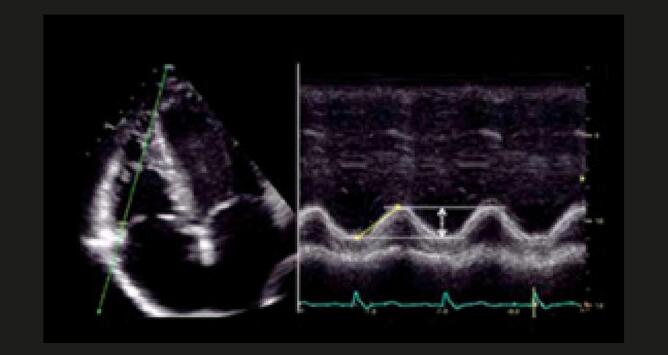 **	Excursão longitudinal do anel tricúspide medido usando-se o modo-M, entre o fim da diástole e o pico sistólico. Janela apical, corte 4 câmaras focado no VD, para alinhamento adequado entre o cursor do modo-M e a excursão da direção longitudinal do VD (anel tricúspide lateral). VN=24 ± 3,5mm (>17mm)
**FUNÇÃO SISTÓLICA LONGITUDINAL DE VD AVALIADA PELA ONDA “ S’ ” DO *DOPPLER* TECIDUAL**	
** 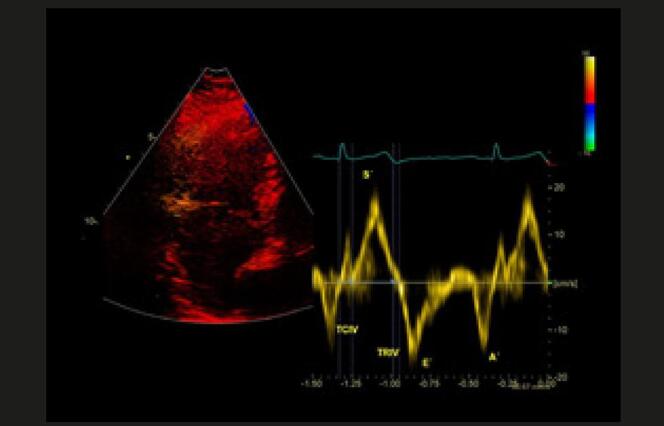 **	Velocidade de pico sistólico do anel tricúspide avaliado por meio do uso de *Doppler* tecidual pulsado (cm/s), através da janela apical e corte de 4 câmaras focado no VD. Importante manter segmento basal e anel alinhados com o *Doppler* para evitar subestimativa dessa medida. VN = 14,1 ± 2,3cm/s (>9,5cm/s)
**FUNÇÃO SISTÓLICA LONGITUDINAL DE VD AVALIADA ATRAVÉS DO *STRAIN* DE LONGITUDINAL GLOBAL**	
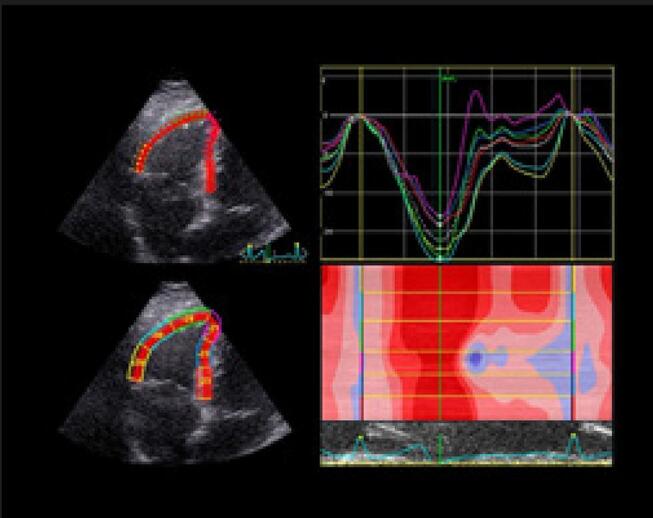	O *strain* longitudinal do ventrículo direito deve ser medido através do corte apical 4 câmaras focado na avaliação do VD. O painel mostra o *strain* global longitudinal dos 6 segmentos (3 da parede livre e 3 segmentos septais); calcula-se a média dos valores. O *strain* longitudinal do VD é realizado, muitas vezes, por *software* não dedicado a essa câmara. Hoje, temos *softwares* dedicados para VD, que devem ser os de preferência e fornecem dados do *strain* da parede livre do VD e do *strain* que incorpora o septo interventricular. VN: 29 ± 4.5 ( 20% em valor absoluto)
**MEDIDA DO VOLUME DE ÁTRIO DIREITO (VOLUME PELO ECO2D)**	
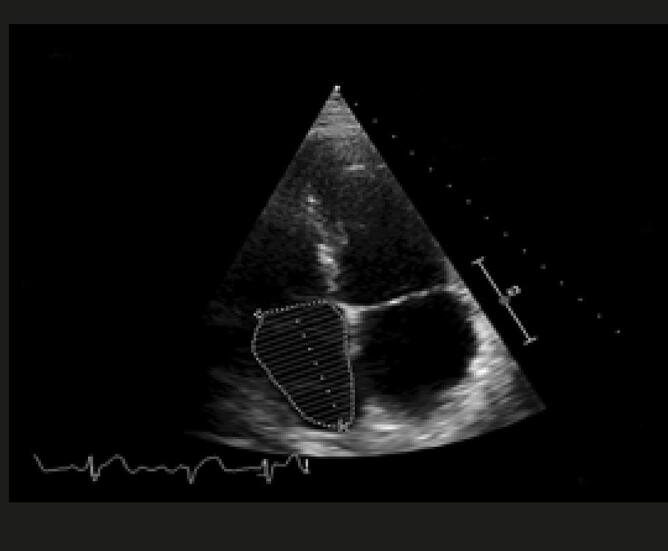	O parâmetro recomendado para avaliar o tamanho do AD é seu volume calculado usando a técnica com plano único do somatório dos discos (2D), no corte apical 4 câmaras realizado adequadamente. VN: Homem 25 ± 7mL/cm ^2 18-32^ Mulher 21 ± 6mL/cm ^2 15-27^
**MEDIDA DAS DIMENSÕES DA VEIA CAVA INFERIOR (VCI)**	
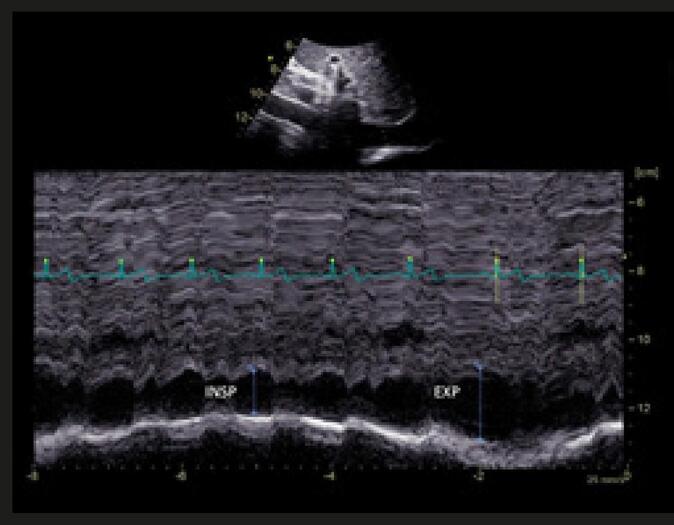	Janela subcostal, posição supina, cerca de 1-2 cm da junção com o AD (perpendicular ao eixo longo). O “índice de colapsabilidade” (% de redução do diâmetro da VCI durante a inspiração) se correlaciona com a pressão em AD. VCI ≤ 21mm e IC > 50% = pressão AD normal (3mmHg, variando entre 0-5 mmHg). VCI ≤ 21mm e IC < 50% = pressão intermediária (8mmHg, variando entre 5-10mmHg). VCI > 21mm e IC < 50% = pressão elevada 15mmHg, variando entre 10-20mmHg).


*TSVD: trato de saída do VD; ASC: área de superfície corporal; VD: ventrículo direito; AD: átrio direito; VCI: veia cava inferior; VN: valor de normalidade.*

## 2.3. Ecocardiograma em Trombembolismo Pulmonar de Alto Risco

Em todos os pacientes com suspeita clínica de TEP e instabilidade hemodinâmica, o ecocardiograma é exame obrigatório, pois consegue detectar com segurança alterações em cavidades direitas tradutoras do aumento súbito e grave da resistência vascular pulmonar (pós-carga de VD), responsáveis por disfunção e hipotensão. Nesses pacientes, o registro de um exame normal elimina em definitivo a hipótese de embolia pulmonar maciça. ^39 , 43^ Por outro lado, a detecção de alterações correspondentes à sobrecarga de pressão e à disfunção ventricular direita (na ausência de outras causas óbvias de diagnóstico diferencial) permite o diagnóstico de TEP maciço e a terapia de reperfusão de emergência, mesmo na impossibilidade de realização de angiotomografia de tórax ou cintilografia de ventilação e perfusão. ^44^

**Figura 1:**
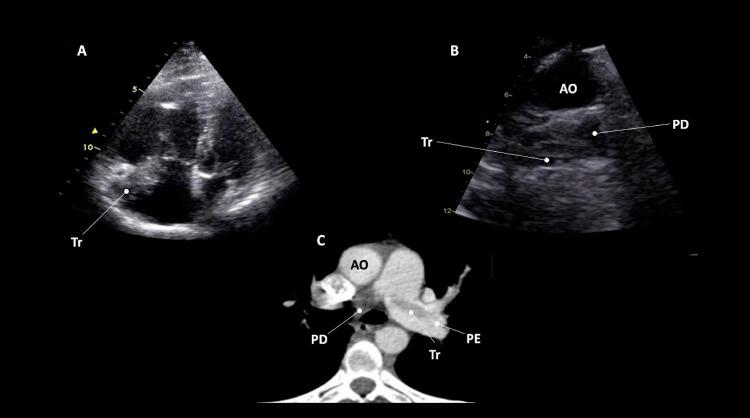
– A-C: visibilização direta de trombo em cavidades direitas e artéria pulmonar esquerda. Tr: trombo; Ao: aorta; PE: artéria pulmonar esquerda; PD: artéria pulmonar direita.

O único achado ecocardiográfico patognomônico de TEP é a presença de trombos móveis em cavidades direitas, tronco e/ou ramos de artéria pulmonar ( Figura 1 ). Tal achado associa-se a alta mortalidade precoce ^45 - 49^ e ocorre em apenas 4% dos casos de embolia pulmonar em geral, podendo alcançar 18% de prevalência nos pacientes com TEP em unidades de terapia intensiva. ^50 , 51^

- a) As alterações fundamentais relativas ao trombembolismo agudo grave ^52^ são:
- b) Dilatação de VD;
- c) Disfunção de VD;

Sobrecarga pressórica pulmonar. Ocorre em 30-40% dos pacientes com TEP e indica pior prognóstico. ^53 - 55^

**Figura 2:**
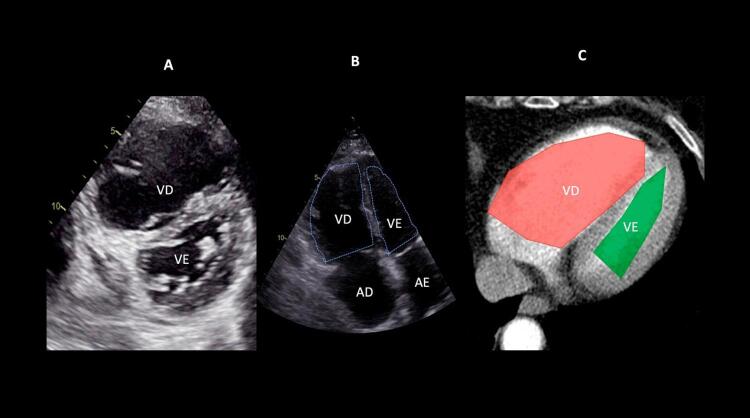
– Sobrecarga pressórica e/ou disfunção do ventrículo direito (VD) A: retificação do septo ventricular com dilatação do VD ao corte paraesternal transverso; B: dilatação do VD avaliada ao corte apical pela relação área diastólica final VD/VE (>0,6 leve; 1-2 importante; >2 severa); C: equivalente da relação VD/VE avaliada pela angio-TC. VD: ventrículo direito; VE: ventrículo esquerdo; AD: átrio direito; AE: átrio esquerdo.

**Figura 3:**
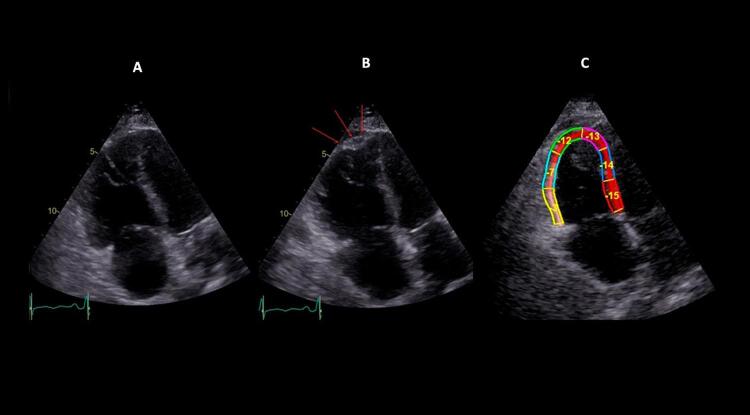
– Sinal de McConell. Observam-se dilatação do ventrículo direito, hipocinesia de parede livre e preservação da contratilidade apical. A: diástole; B: sístole; C: strain longitudinal do ventrículo direito (VD); ventrículo esquerdo (VE); átrio direito (AD); e átrio esquerdo (AE).

**Figura 4:**
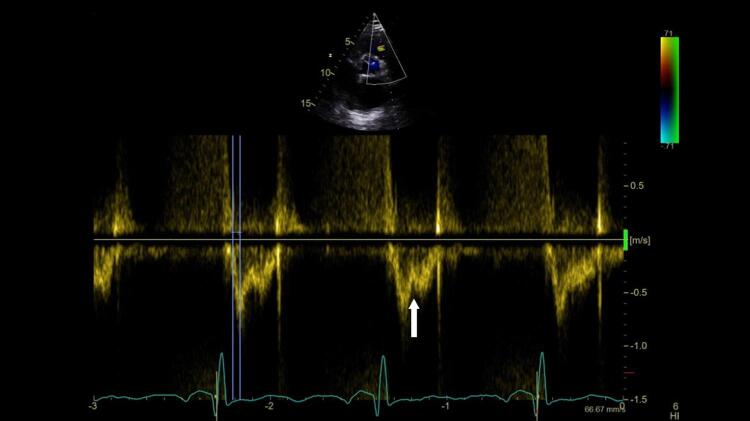
– Perfil hemodinâmico do fluxo da via de saída do ventrículo direito (VSVD) causada pelo aumento do pós-carga do VD: redução do tempo de aceleração e presença de desaceleração mesossistólica (notch – seta branca).

Quando a dilatação da cavidade ventricular ( Figura 2 ) associa-se ao sinal 60/60 – combinação de tempo de aceleração do fluxo de ejeção pulmonar <60ms e ao gradiente de pico sistólico valvar tricúspide <60 mmHg –, ou com sinal de McConell – hipocinesia de segmentos basal e médio de parede livre do VD e contratilidade normal da região apical ( Figura 3 ) –, o valor preditivo positivo para embolia pulmonar maciça é alto. ^56^ No entanto, o sinal 60/60 está presente em apenas 12%; e o sinal de McConell em 20% dos pacientes não selecionados. Outro sinal de aumento da pós-carga do VD é redução do tempo de aceleração pulmonar e a presença de desaceleração mesossistólica ( Figura 4 ). Sinais de sobrecarga pressórica de VD ajudam a diferenciar hipocinesia ou acinesia de parede livre decorrente de TEP daquela causada por infarto agudo de ventrículo direito (que pode mimetizar o sinal de McConell). ^52 , 57^

Pacientes com embolia pulmonar também podem apresentar excursão sistólica do plano do anel tricúspide (Tapse) reduzido. ^58 , 59^ O *Doppler* tecidual e o *strain* parietal do VD apresentam baixa sensibilidade como achados isolados nos pacientes com TEP agudo. ^60 , 61^ Nos pacientes com suspeita de TEP agudo, mas que apresentem espessura aumentada da parede livre de VD ou velocidades do jato regurgitante valvar tricúspide acima >3,8 m/s, ou gradiente de pico sistólico valvar tricúspide >60 mmHg, devemos incluir diagnóstico diferencial com hipertensão arterial pulmonar trombembólica crônica. ^38 , 62^

**Figura 5:**
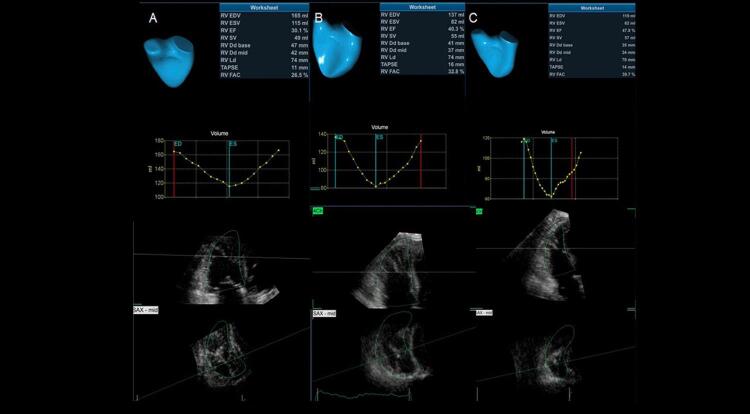
– Análise evolutiva ao eco 3D do ventrículo direito (VD), com medida dos volumes diastólico final (VDFVD), sistólico final (VSFVD) e de fração de ejeção (FEVD). Paciente com trombembolismo pulmonar submaciço em 3 momentos: A: pré-trombólise (VDFVD = 165mL, VSFVD = 115mL, FEVD = 30%); B: segundo dia pós-trombólise (VDFVD = 137mL, VSFVD = 82mL, FEVD = 40%); C: quinto dia pós-trombólise (VDFVD = 113mL, VSFVD = 62mL, FEVD = 47,8%).

O ecocardiograma 3D pode ser utilizado em laboratórios com experiência na técnica, para a avaliação da análise evolutiva dos pacientes com TEP maciço e submetidos a trombólise. Nas Figuras 5 e 6 , está exemplificado um caso de TEP maciço, com importante repercussão hemodinâmica, tratado com trombolítico, evoluindo para um Quadro de melhora da fração de ejeção do VD e redução significativa dos volumes cavitários direitos.

**Figura 6:**
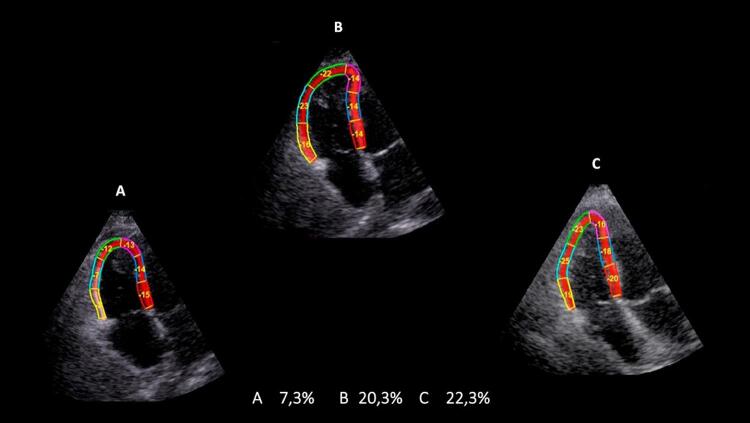
– Análise evolutiva do strain longitudinal da parede livre do ventrículo direito (VD) em valores absolutos e 3 momentos: A: pré-trombólise; B: segundo dia pós-trombólise; C: quinto dia pós-trombólise com valores de strain normalizados.

No início de 2020, a pandemia do novo coronavírus (covid-19) acrescentou um novo e crucial papel para a ecocardiografia. Até o momento, os achados registrados em publicações indicam que o vírus causa aumento significativo no risco de trombose venosa (traduzida por elevação do D-dímero) e, consequentemente, da ocorrência de TEP. Por outro lado, parece provocar trombose local em vasos da microcirculação pulmonar, gerando sobrecarga pressórica sobre o ventrículo direito. Ambos os fatores podem resultar em dilatação e disfunção de VD, mensurados por vários parâmetros, como citado no Quadro 8 . Esses pacientes evoluem com insuficiência respiratória aguda, exigindo ventilação mecânica invasiva sob pressão positiva, o que também contribui para o aumento de pós-carga ao VD. Nesses pacientes, as alterações ecocardiográficas são muito semelhantes (dilatação e disfunção de VD com hipertensão pulmonar), e nem sempre é possível diferenciar a causa preponderante. Em publicação recente de uma coorte multinacional de 870 pacientes em internação hospitalar por covid-19, o *strain* da parede livre do VD foi um marcador prognóstico independente de mortalidade sem a análise de outros parâmetros como Tapse e S’ do anel tricúspide. ^63^ Além do *strain* do VD, a dilatação dele foi um marcador prognóstico de pacientes com a forma grave da doença. ^64^ Concluindo, o papel da ecocardiografia nesse cenário é fundamental, não só do exame completo como também do ecocardiograma focado ( *point of care* ), recentemente abordado pelo posicionamento do DIC/SBC. ^65^

**Quadro 8:** – Sumário de alterações ecocardiográficas no tromboembolismo pulmonar (TEP) agudo

PARÂMETROS	ALTERAÇÃO ECOCARDIOGRAMA	IMAGEM
Dimensões da cavidade ventricular direita	Eco 2D: diâmetro linear basal > 4,2 cm e longitudinal > 8,1 cm. Eco 3D: volume diastólico final > 87 ml/m ^2^ em homens e 74 mL/m ^2^ em mulheres; volume sistólico final > 44 mL/m ^2^ em homens e 36 mL/m ^2^ em mulheres. Relação de dimensões VD/VE > 1,0	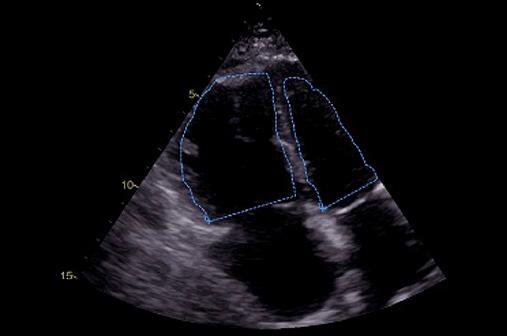
Função sistólica do VD	Hipocinesia ou acinesia de paredes. FAC < 35%. TAPSE < 17 mm. *Doppler* tecidual: onda S’ < 9,5 cm/s. “ *Strain* ” longitudinal de parede livre acima de -20% (< 20% em valor absoluto). Eco 3D: fração de ejeção < 45%.	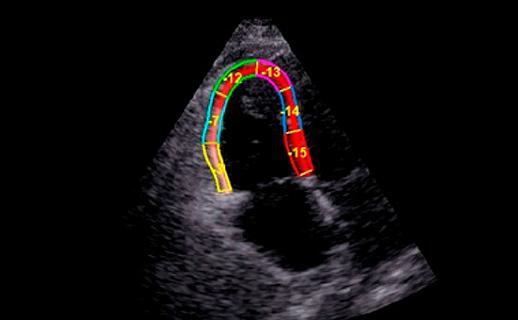
Septo interventricular	Retificação ou deslocamento para esquerda devido à sobrecarga pressórica em VD.	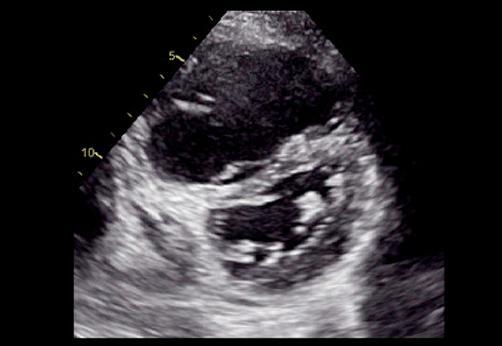
Diâmetro do tronco de artéria pulmonar	Dilatação (acima 3,0 cm diâmetro).	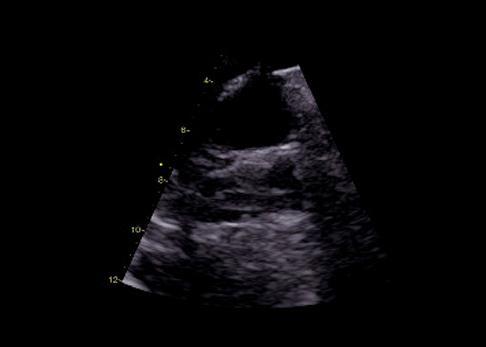
Refluxo tricúspide	Aumento do refluxo tricúspide devido ao aumento da pressão sistólica em artéria pulmonar.	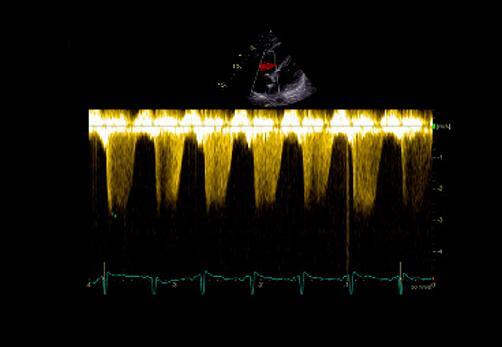
“Sinal de 60/60”	TAC do fluxo pulmonar < 60ms com entalhe mesossistólico + gradiente de pico sistólico da regurgitação tricúspide < 60 mmHg.	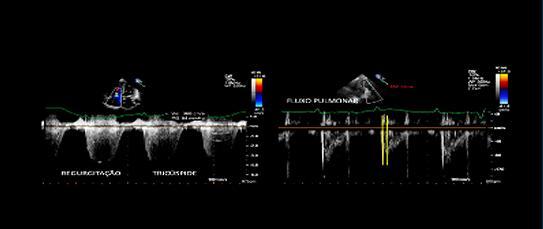
“Sinal de McConell”	Hipocinesia ou acinesia de segmentos basal e médio de parede livre do VD e contratilidade preservada do segmento apical.	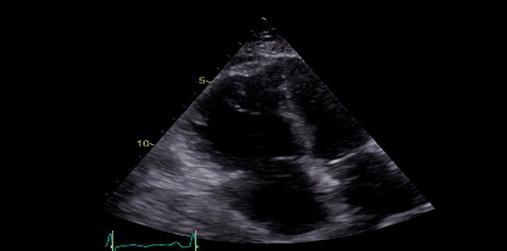
Cava inferior	“Índice de colapsabilidade” < 50% durante a inspiração.	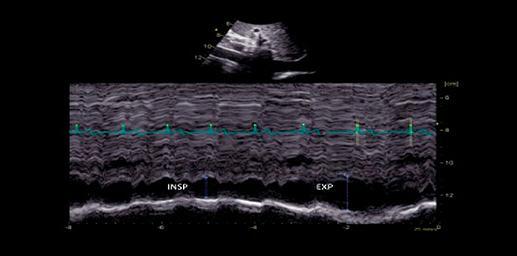
Trombo intracavitário	Único achado patognomônico [presente em cavidades direitas e/ou tronco (ou ramos) de artéria pulmonar em apenas 4% dos casos].	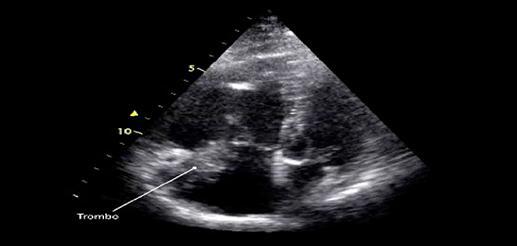

## 2.4. Recomendações

1. O ecocardiograma a beira do leito é exame obrigatório em todo paciente com suspeita clínica de TEP e instabilidade hemodinâmica. ^39 , 43 , 44^ (classe I/nível de evidência C).
2. O ecocardiograma não é exame obrigatório na rotina diagnóstica em paciente com suspeita de TEP e hemodinamicamente estável, nos algoritmos de investigação. ^1 ,^ ^35 - 37^ No entanto, representa uma importante ferramenta para discriminação e definição do diagnóstico diferencial. ^38 - 41^ (classe IIa/nível de evidência B).

## 3. Ultrassonografia Vascular no Diagnóstico da Trombose Venosa Profunda

### 3.1. Sinais Ultrassonográficos da Trombose Venosa: Modo B e *Doppler*

A trombose venosa profunda (TVP) é uma condição que deve ser diagnosticada e tratada adequadamente, podendo levar ao tromboembolismo pulmonar (TEP), situação potencialmente fatal. ^66^ Os trombos originam-se dentro do sistema venoso, muitas vezes em uma área de fluxo mais lento, como a cúspide de uma válvula, propagando-se ao longo do lúmen da veia, que é preenchido do modo parcial ou total pelo trombo. Na fase aguda, os trombos induzem uma resposta inflamatória na parede venosa adjacente; dessa maneira, o termo tromboflebite é aplicado, com sintomas de câimbras e dor local. ^67^ Muitos fatores podem iniciar a trombose venosa: no caso de cateteres venosos, o traumatismo da parede inicia a resposta inflamatória que leva a trombose dentro do lúmen venoso. ^68 , 69^ Entretanto, os sinais clínicos da TVP muitas vezes podem ser inespecíficos; portanto, a anamnese, o exame físico e o conhecimento dos principais fatores que se relacionam com o processo trombótico são fundamentais. ^16 , 70^

Os fatores de risco para trombose venosa podem ser agrupados em modelos de predição clínica, sendo o escore de Wells um método bem estabelecido pelo qual se pode acessar a probabilidade clínica do diagnóstico da TVP. Uma vez que o diagnóstico clínico da TVP possa estar correto em apenas 50% dos casos, exames complementares são necessários para confirmar ou excluir o diagnóstico. ^70^ De modo geral, a avaliação dos pacientes com suspeita de TVP passa por estratificação, escore clínico, dosagem do D-dímero e realização da ultrassonografia vascular (USV). ^6^

#### 3.1.1. Metodologia de Realização do Exame – Aspectos Técnicos

A USV é o exame de escolha na suspeita de TVP, e fornece informações da anatomia e funcionalidade venosa. Não é um procedimento invasivo, não usa contraste nefrotóxico e é reprodutível e de baixo custo, mas é examinador e depende de equipamento. ^71^ Dessa forma, analisamos as imagens da parede e do lúmen venoso em tempo real no modo bidimensional (modo B), realizando as manobras de compressão com o transdutor e o estudo do mapeamento de fluxo em cores (MFC), *Doppler* pulsado e *Power Doppler* . ^72^

Como publicado recentemente pelo Departamento de Imagem Cardiovascular, ^73^ a técnica para realização do exame de pesquisa de TVP inclui os seguintes itens.

#### a) Posicionamento do paciente:

Para membros inferiores, o paciente deve ser posicionado em decúbito dorsal, com o tronco e cabeça elevados até 30°, posição confortável, próximo da borda do leito, do mesmo lado do examinador e fazendo uma leve rotação lateral da articulação coxofemoral e flexão do joelho. Para o exame das veias femorais comum e femoral superficial, e tibiais posteriores, o paciente deve manter a perna em rotação externa e o joelho levemente fletido. Para o exame da veia poplítea e das veias fibulares (peroneiras), o paciente pode ficar em decúbito ventral ou lateral, apoiado no membro contralateral e com o joelho levemente fletido. Neste último caso, se houver condições clínicas, o paciente pode permanecer sentado, com as pernas pendentes ao lado da cama. ^74^ Para o exame das tibiais posteriores, fibulares e musculares da panturrilha, eventualmente podemos manter o paciente em decúbito dorsal e a perna dobrada com o pé apoiado na cama. Por outro lado, o estudo das veias cavas e ilíacas é realizado com o paciente em decúbito dorsal, lembrando-se da necessidade de preparo prévio para diminuição dos gases intestinais. Na suspeita de síndrome pós-trombótica, o paciente, quando possível, deve ser examinado também em posição ortostática, e para membros superiores, deve ser posicionado em decúbito dorsal, com o membro estendido paralelamente ao corpo e discretamente afastado dele.

#### b) Ajustes do aparelho de US:

Deve-se trabalhar com equipamentos capazes de fornecer uma boa qualidade de imagem em modo B e que sejam dotados de *Dopplers* espectral e colorido. A escolha do transdutor baseia-se primordialmente na relação frequência do transdutor e da localização do objeto a ser estudado. Transdutores de maior frequência fornecem melhor qualidade de imagem, porém têm menor penetração nos tecidos. Em contrapartida, os transdutores de menor frequência permitem uma maior penetração, ainda que ocorra perda de qualidade da imagem. Habitualmente, são utilizados transdutores lineares com frequência entre 4 a 10 MHz para estudo das veias profundas dos membros inferiores ou superiores, e transdutores lineares com frequência entre 7 a 13 MHz para estudo das veias superficiais dos membros inferiores ou superiores. Nos casos de membros inferiores edemaciados e em pacientes obesos, ou seja, com diâmetros muito aumentados e, também, para a avaliação das veias ilíacas e da veia cava inferior, por vezes torna-se necessário a utilização de transdutores convexos multifrequenciais (entre 1 a 5 Mhz), os quais exibem frequências de onda menores e maior penetração das ondas de ultrassom. Os *presets* devem ser adequadamente calibrados para o exame do sistema profundo e superficial, com zona focal ajustada e escala de cinza, de modo que o lúmen seja escuro na ausência de estase ou trombose. O ganho do *Doppler* , seja colorido ou espectral, deve ser ajustado para uma escala de velocidades baixas e adequada na avaliação do fluxo venoso. Quando o exame se direciona à avaliação das veias profundas, os equipamentos de USV devem ser ajustados com foco e densidade de linha adequados para o exame bidimensional e as estruturas são avaliadas em cortes transversos e longitudinais. ^73^ Em geral, as veias são facilmente compressíveis pelo transdutor. ^71^

#### c) Sequência do exame:

**Figura 7:**
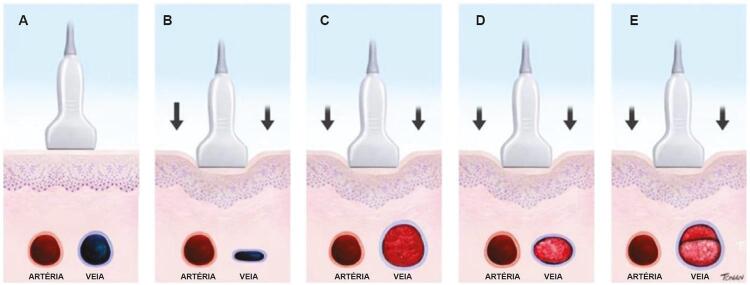
– A-E: Esquema de avaliação da compressibilidade venosa pelo transdutor do ultrassom utilizado na pesquisa de trombose venosa. A: artéria e veia sem compressão pelo transdutor. B: veia normal com compressão total. C: veia dilatada e incompressível, com trombo de aspecto agudo/recente. D: veia de calibre normal a reduzido, pouco compressível pela manobra com o transdutor e compatível com achados de trombose venosa antiga/crônica. E: recorrência de trombose.

**Modo B:** inicialmente, o transdutor é posicionado abaixo do ligamento inguinal, visualizando-se a junção safenofemoral. Faz-se, então, uma varredura transversa e longitudinal de todo o sistema venoso do membro inferior. No corte transverso, deve ser realizada a compressão com o transdutor, em especial ao longo da veia femoral comum, da veia femoral e da veia poplítea, com distâncias entre cada compressão em torno de 2 a 3 cm. A técnica de uso da USV para diagnóstico da TVP foi inicialmente descrita por Talbot em 1982, e vem sendo aprimorada ao longo dos anos. ^75^ Uma veia normal e sem trombose demonstra completo colabamento luminal pela compressão de suas paredes pelo transdutor ( Figuras 7 e 8 ).

**Figura 8:**
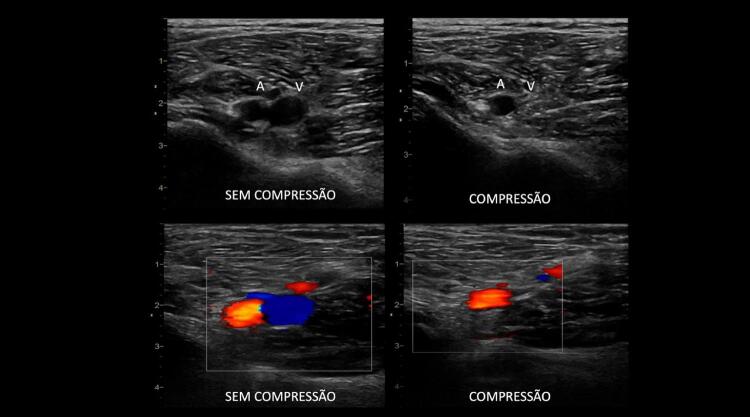
– Corte ultrassonográfico transversal da artéria e veia femoral comum para teste de compressibilidade venosa.

As figuras demonstram a compressibilidade completa da veia femoral comum ao modo B (superiores) e com mapeamento de fluxo em cores (inferiores). A = artéria, V = veia.

**Figura 9:**
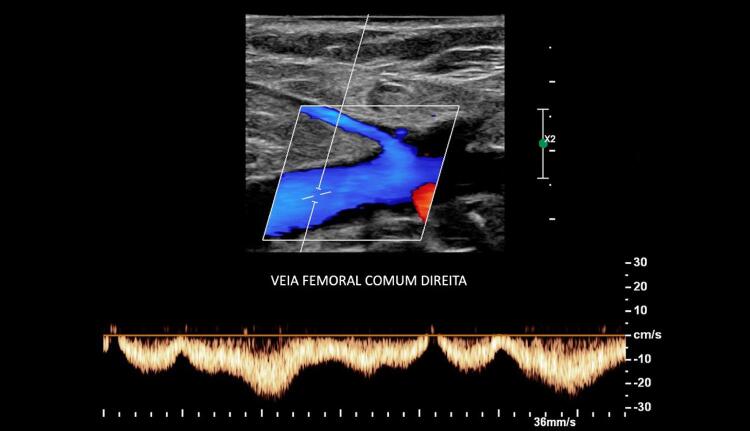
– Doppler pulsado em veia femoral comum demonstrando fluxo normal, fásico com os movimentos respiratórios e compatível com ausência de trombose oclusiva.

***Doppler* :** Objetiva-se registrar as velocidades do fluxo venoso, que são baixas em condições normais ao *Doppler* pulsado. Na presença de trombose venosa, as velocidades podem estar ainda mais reduzidas, necessitando de ajustes em suas escalas. O fluxo venoso nos membros inferiores é espontâneo e apresenta-se fásico com a respiração e as variações da pressão intra-abdominal ( Figura 9 ). Na fase inspiratória, as velocidades diminuem por conta da elevação na pressão intra-abdominal; por outro lado, a manobra de compressão distal (compressão da panturrilha, p. ex.) demonstra um pico de fluxo na veia interrogada quando esta se encontra patente. Em alguns casos, a detecção adequada do fluxo só é possível através do uso do *Power Doppler* , que, apesar de não demonstrar sua direção, consegue detectar fluxos de velocidade muito baixos.

#### d) Alterações ao modo B, *Doppler* colorido e espectral no diagnóstico da TVP aguda/recente:

O diagnóstico ultrassonográfico da TVP aguda/recente baseia-se na alteração da compressão com o transdutor, total ou parcial da veia acometida, bem como a dilatação se dá pela presença de material trombótico intraluminal. A alteração na compressibilidade da veia, mesmo diante de um Quadro muito recente de TVP, talvez seja o achado mais importante para o diagnóstico de trombose venosa. A presença de trombo só pode ser excluída quando, após a compressão, ocorrer o desaparecimento total do lúmen venoso. Caso isso não aconteça completamente, o lúmen pode estar parcialmente preenchido com trombos. Para tal procedimento de compressão, é adequado que seja observada a artéria adjacente.

**Figura 10:**
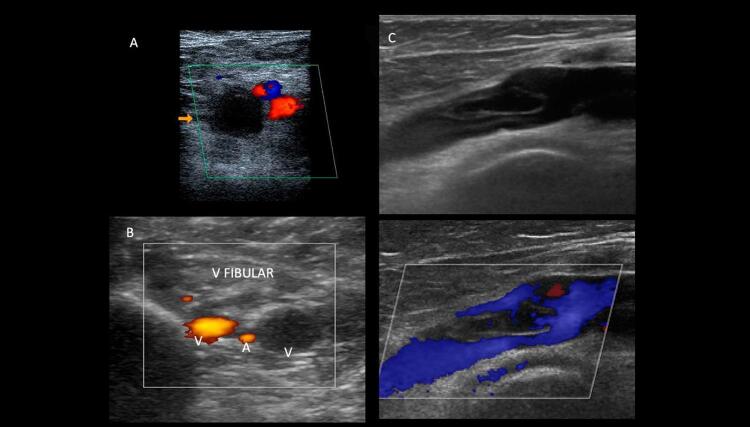
– Trombose oclusiva e não oclusiva de aspecto agudo ao ultrassom. A: corte transversal da veia femoral comum; B: veias fibulares evidenciando dilatação e incompressibilidade sem evidências de fluxo ao mapeamento de fluxo em cores e ao Power Doppler; C: corte longitudinal da veia femoral comum com e sem mapeamento de fluxo em cores evidenciando trombo flutuante de aspecto recente e não oclusivo.

Na maioria das vezes, as veias recentemente trombosadas apresentam-se distendidas, com diâmetros maiores que os vasos arteriais adjacentes. Essa distensão venosa também é importante na diferenciação entre trombos agudos e antigos. Quando o trombo é oclusivo, há dilatação venosa e ausência de fluxo detectável tanto ao *Doppler* pulsado quanto ao MFC e ao *Power Doppler* – Figura 10 A e B . A dilatação venosa já foi descrita como um parâmetro acurado para identificar a TVP aguda. ^76^

Primariamente, o trombo agudo é composto por uma densa malha de fibrina, que persiste por aproximadamente 5 a 7 dias. Nessa fase, o trombo é mais ecolucente ou tem ecogenicidade intermediária, com brilho menor que os tecidos adjacentes. O diagnóstico do trombo agudo/recente é importante, pois em tal fase pode ser melhor tratado com terapia anticoagulante e fibrinolítica. Em geral, considera-se a trombose venosa como aguda nas primeiras 2 semanas de seu início. ^77^ Muitas vezes, na extremidade de um trombo agudo, forma-se um coágulo mais recente e que flutua livre dentro da veia. Nesses casos, convém maior cautela para evitar o deslocamento dele, requerendo uma menor manipulação do paciente ( Figura 10 A-C ). Adicionalmente, devem ser avaliados, além da compressibilidade venosa, o aspecto da parede venosa (paredes finas e lisas costumam estar presentes na TVP aguda), o tamanho do lúmen da veia, a funcionalidade das válvulas e a presença de circulação colateral.

Quanto ao fluxo, dependendo do grau de obstrução total ou parcial do lúmen venoso, pode estar diminuído ou ausente, com ausência de variação respiratória. Entretanto, um lúmen parcialmente obstruído pode não alterar os sinais de fluxo ao *Doppler* .

#### e) Alterações ao modo B e *Color Doppler* no diagnóstico da TVP clinicamente subaguda e na TVP crônica/antiga: 11 

**Figura 11:**
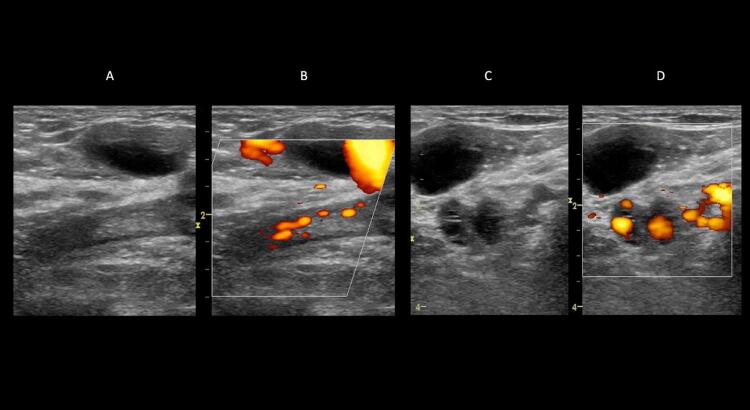
– Alterações crônicas pós-trombóticas. A: veia femoral ao modo B sem dilatação, com imagem ecogênica luminal correspondendo a alterações crônicas pós-trombóticas; B: fluxo luminal reduzido ao Power Doppler; C: veias tibiais posteriores com paredes espessadas ao modo B; D: preenchimento parcial da luz do vaso das veias tibiais posteriores pelo Power Doppler.

Em geral, na avaliação clínica da TVP, considera-se a trombose venosa como aguda nas primeiras 2 semanas do seu início; subaguda, entre 2 semanas e 6 meses; e crônica, após 6 meses de evolução. ^72 , 78^ Na evolução da TVP, o trombo torna-se mais ecogênico; no entanto, como tal alteração é variável, muitas vezes não é possível estimar de maneira precisa sua idade apenas avaliando a ecogenicidade dele. Por essa razão, quando encontramos um trombo anecoico ou hipoecoico, com poucos dias de evolução, corrobora-se o diagnóstico de trombose aguda. Porém, quando temos uma imagem intraluminal isoecoica ou hiperecoica, fica muito difícil nesse intervalo estimar a idade do trombo com exatidão. Durante tal período, podem ocorrer lise ou retração do trombo, com a veia menos distendida; por isso a importância do exame seriado. Com a retração do trombo e sua lise, o fluxo pode ser reestabelecido não necessariamente ao normal, e as paredes da veia tornam-se espessas ( Figura 11 ). ^71 , 72 , 79^ Desse modo, pela USV, nem sempre é possível determinar a idade do trombo por sua aparência ou sua ecogenicidade. ^71 , 72 , 79^

Após a trombose aguda, caso o trombo não desapareça por completo, ele se torna infiltrado por fibroblastos, se organiza e reendoteliza. A fibrose produz cicatrizes, espessamento das paredes e sinéquias que podem causar obstrução parcial do vaso e permanecer por anos; esse material residual não deve mais ser chamado de trombo. O espessamento da parede venosa é um achado comum, o calibre pode estar reduzido e o fluxo apresenta-se alterado, dependendo do calibre venoso. Em alguns casos, cicatrizes fibróticas pós-trombóticas surgem com a evolução do Quadro e produzem, ao longo da parede venosa, imagens semelhantes a placas, pois são ecogênicas e eventualmente apresentam calcificações com sombras acústicas. A Sociedade Americana de Radiologistas sugeriu, em consenso publicado em 2018, que as imagens ultrassonográficas referentes a essas alterações, que persistem após o evento agudo da TVP, fossem denominadas “alterações crônicas pós-trombóticas”. Portanto, as anormalidades encontradas na TVP, identificadas à ultrassonografia, deveriam ser classificadas, segundo esses autores, como: “trombose venosa aguda, alterações crônicas pós-trombóticas ou alterações indeterminadas”, estas últimas, quando os achados são duvidosos, e não é possível, apenas pela USV, determinar a idade do trombo, como nos casos de TVP clinicamente subaguda. De acordo com essa recomendação, alguns casos podem ser enquadrados em caráter experimental como trombose subaguda no laudo ultrassonográfico como:

1. Diante de exame prévio mostrando a TVP aguda;
2. Em estudo subsequente que mostre alteração da aparência do trombo. ^80^

Esses conceitos são corroborados por recente publicação da SBACV. ^81^ Entretanto, em consenso, seus autores, com o receio de que o uso do termo “alterações indeterminadas” possa gerar confusão para o clínico solicitante quanto à necessidade de iniciar o tratamento ou não, sugerem que o ultrassonografista vascular informe as características da imagem de forma completa, analise a informação clínica em relação ao tempo de ocorrência e dê preferência a concluir como “predominância de alterações agudas ou recentes” ou “predominância de alterações crônicas ou antigas”. Dessa forma, o termo indeterminado seria usado em poucas ocasiões, nas quais não seja realmente possível avaliar o tempo do evento.

**Figura 12:**
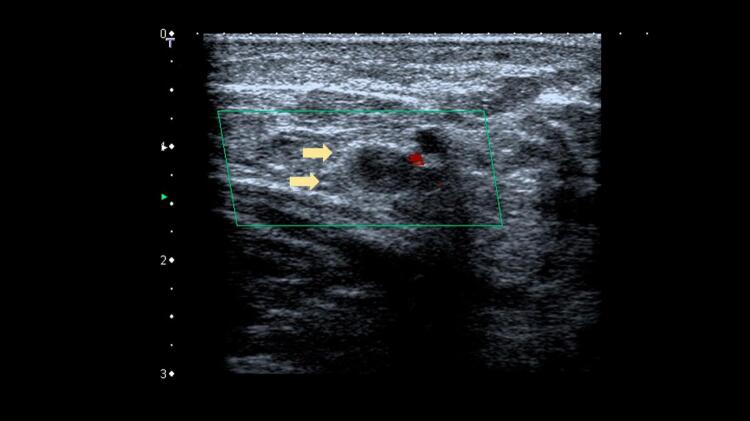
– Trombose venosa de veias plantares incompressíveis e sem fluxo no mapeamento de fluxo a cores.

A TVP pode acometer diversos segmentos venosos em conjunto ou isoladamente. Um exemplo é a trombose de veias plantares, não avaliada com frequência. Deve-se buscar esse diagnóstico quando há a presença de dor aguda e/ou edema no pé. Os aspectos ultrassonográficos da TVP plantar recentemente observados são a dilatação venosa e a presença do trombo luminal com redução da compressibilidade venosa ( Figura 12 ).

O Quadro 9 , adaptado de *Gornik HL and Sharma AM* , ^72^ demonstra as alterações ultrassonográficas que podem ser encontradas na TVP aguda/recente, crônica/antiga ou de características agudas ou crônicas. Devemos sempre lembrar que, em alguns casos, há sobreposição das características ultrassonográficas, que devem ser relatadas como “idade indeterminada”.

**Quadro 9:** – Características ultrassonográficas na trombose venosa profunda (TVP)

Característica	Aguda/Recente	Predomínio de trombo agudo ou Predomínio de trombo crônico	Pós-trombótica Crônica/Antiga
Ecogenicidade do trombo	Hipoecoico ou isoecoico	Variável (mais ecogênico que na TVP aguda)	Hiperecoico
Presença de trombo móvel	Pode estar presente	Geralmente ausente	Ausente
Aderência do trombo à parede venosa	Fracamente aderido	Firmemente aderido	Traves fibróticas hiperecogênicas no lúmen ou na parede
Aparência da parede venosa	Variável	Variável	Paredes espessadas, cicatrizes. Pode haver depósito de cálcio
Lúmen venoso	Dilatado	Retraindo para o tamanho normal	Menor que o tamanho normal
Compresibilidade	Pouco compressível ou levemente deformável	Mais compressível que o agudo	Parcialmente compressível
Compressibilidade	Geralmente ausente	Pode estar presente	Pode estar presente
Função das válvulas venosas	Geralmente competentes	Pode haver incompetência	Geralmente incompetentes

**Figura 13:**
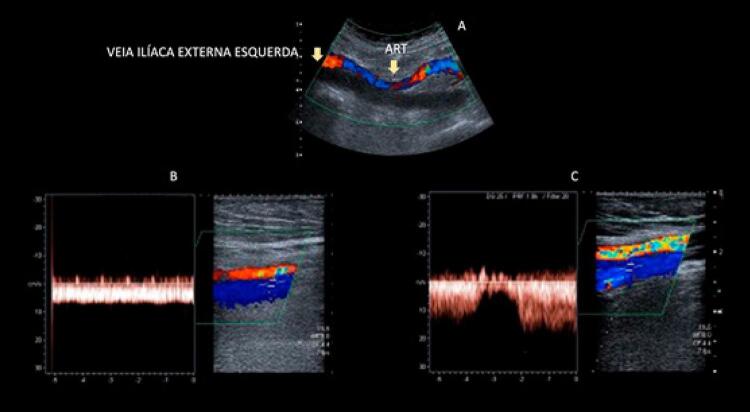
– Trombose de veia ilíaca externa esquerda: comparação de fluxo nas veias femorais comuns esquerda e direita. A: veia ilíaca externa esquerda dilatada, com a luz preenchida por trombo de aspecto recente, sem fluxo ao mapeamento de fluxo a cores; B: fluxo contínuo ao Doppler espectral em veia femoral comum esquerda, secundário a trombose de veia ilíaca externa ipsolateral; C: fluxo fásico e normal na veia femoral comum contralateral.

O comprometimento da fasicidade respiratória e a redução na velocidade do fluxo em um vaso venoso de aspecto normal, com fluxo presente e compressível, podem estar relacionados com o acometimento trombótico em segmento venoso proximal ao vaso que está sendo avaliado ( Figura 13 ).

## 3.2. Protocolos de Realização de USV na TVP

O USV é utilizado desde a década de 1960 para o diagnóstico da trombose venosa e vem se aperfeiçoando ao longo do tempo com estudos sobre a medida do fluxo sanguíneo venoso e a compressibilidade venosa. Como dito anteriormente, a avaliação da compressibilidade venosa em combinação com a cor e o *Doppler* espectral do fluxo é a abordagem recomendada para a detecção do coágulo venoso. Isso se deve, em parte, à disponibilidade, à facilidade de implantação em uma variedade de contextos clínicos e à sua acurácia.

Desde os estudos de acurácia em pacientes sintomáticos e assintomáticos com suspeita de TVP supra ou infrageniculares, a USV é o método de referência para o diagnóstico. ^82^ Atualmente, o US de compressão das veias dos membros inferiores substitui amplamente a venografia para diagnosticar TVP, pois apresenta sensibilidade acima de 90% e especificidade de cerca de 95% para o diagnóstico de tromboses proximais. Nas tromboses distais, a sensibilidade do método cai para cerca de 65%, mas mantém especificidade acima de 90%. Em contrapartida, o ultrassom vascular completo, ou seja, a associação de modo B e o *Doppler* de todas as veias, é o exame de imagem de primeira linha para o diagnóstico de TVP, com sensibilidade de 96% e especificidade de 98% a 100%. Entretanto, ele se mostra um exame demorado, que requer o transporte do paciente para o local de realização e a disponibilidade imediata de um especialista. ^38 , 83 , 84^ Além disso, para um diagnóstico acurado, o examinador deve estar familiarizado com as indicações e limitações do método. Lembramos que apenas 20% a 30% dos exames com suspeita de TVP são alterados, e 90% dos pacientes com TEP fatal são assintomáticos para TVP. ^16 , 18^ As limitações na realização do exame e, consequentemente, sua acurácia, estão relacionadas com obesidade, restrição de mobilidade, edema importante, e/ou presença de feridas, dispositivos ortopédicos, ataduras/gesso etc. Informações clínicas relevantes e um exame físico focado são elementos essenciais para ajudar na interpretação de um exame de USV na pesquisa de TVP, e o adequado treinamento na realização dos protocolos de exames mostra-se fundamental. Apesar dessas limitações, a grande disponibilidade de aparelhos portáteis, o fácil manejo e a inocuidade tornam essa ferramenta de extrema aplicabilidade na prática diária. Além disso, os avanços na miniaturização eletrônica resultaram no advento de dispositivos de exames de ultrassom de bolso equipados com transdutor de matriz linear, tornando o uso do US de compressão para o diagnóstico de TVP ainda mais difundido. ^85^

Seja qual o protocolo de exame escolhido, a sala de exame deverá estar confortavelmente aquecida para evitar o venoespasmo. Para avaliar a veia cava inferior (VCI), o paciente deverá estar em posição de Trendelenburg reverso. ^71^ A *Society for Vascular Ultrasound* recomenda um tempo de aproximadamente 75 minutos para realizar um exame completo bilateral, com 40-60 minutos para fazer diretamente o exame e 15 minutos para análise de dados clínicos, consulta de exames prévios e preparo da sala e do paciente. ^86^

## 3.3. Protocolos de Exame

Com a maior disponibilidade de aparelhos de USV, que habitualmente apresentam ótima resolução, o treinamento de profissionais não especialistas em imagem vem se tornando muito frequente e ganhando aceitação, sobretudo nos setores de emergência e urgência ou ainda em locais distantes. Assim, a realização de exames para pesquisa de TVP por intensivistas, emergencistas e residentes do último ano de radiologia/US/ecografistas vasculares é uma realidade atual. A acurácia do exame de US realizado por emergencistas foi testada em uma metanálise e revisão sistemática que incluiu 16 estudos com 2.379 pacientes. Os autores encontraram uma prevalência em TVP de 23% (7,4% – 47,3%), com sensibilidade média dos exames realizados por emergencistas de 96,1 % (95% IC; 90,6 – 98,5%) e uma especificidade de 96,8 % (95% IC; 94,6 – 98,1%). Entretanto, os estudos incluídos eram muito heterogêneos, os treinamentos dos emergencistas foram mal descritos e não havia, na maioria deles, um seguimento maior. Um estudo prospectivo publicado no mesmo ano e com um número muito menor de pacientes não reproduziu esses dados, o que refletiu a necessidade de protocolos de treinamento rigorosos desses profissionais, visto que, nesse estudo, apenas os três primeiros exames realizados por não especialistas foram supervisionados. ^84 , 87^ Muitos dos protocolos realizados por não especialistas em imagens vasculares foram desenvolvidos para locais em que não há disponibilidade de profissional especializado ou mesmo para reduzir o número de chamadas fora do horário de funcionamento habitual do laboratório de USV. Assim, apenas um protocolo de simples realização não é suficiente para afastar definitivamente a TVP. Além do adequado treinamento sob supervisão, mostra-se fundamental a análise da probabilidade pré-teste associada, ou seja, o uso de escores clínicos de probabilidade de TEP/TVP e o conhecimento da menor acurácia da USV no diagnóstico da TVP infragenicular e na TVP assintomática. Nas publicações que avaliaram esse tema, não houve prejuízo maior para o paciente, e o seguimento de protocolos bem estruturados, contemplando os critérios de probabilidade clínica de Wells, ajudou na melhora da acurácia do exame e no encaminhamento adequado dos pacientes com difícil acesso ao especialista. ^82 , 88 - 90^

Para a pesquisa de trombose venosa pelo USV, convém analisar os parâmetros descritos, ou seja, a combinação de componentes da escala de cinza (modo B) com manobras de compressão pelo transdutor e avaliação pelo mapeamento de fluxo em cores e *Doppler* espectral. Os protocolos de exames vão usar esses parâmetros para chegar ao diagnóstico: compressibilidade da veia (o mais importante); calibre (diâmetro) da veia, aspecto ecográfico das paredes e do lúmen venoso, e a análise do mapeamento de fluxo em cores e do *Doppler* espectral.

Existem alguns protocolos de pesquisa de TVP bastante estabelecidos, que podem ser completos ou não ( *Point of Care* ). Eles são escolhidos conforme a disponibilidade de aparelho de US no local em que o paciente é atendido, do horário do atendimento e da presença de médico especialista ou não para realizar o exame de USV.

**Figura 14:**
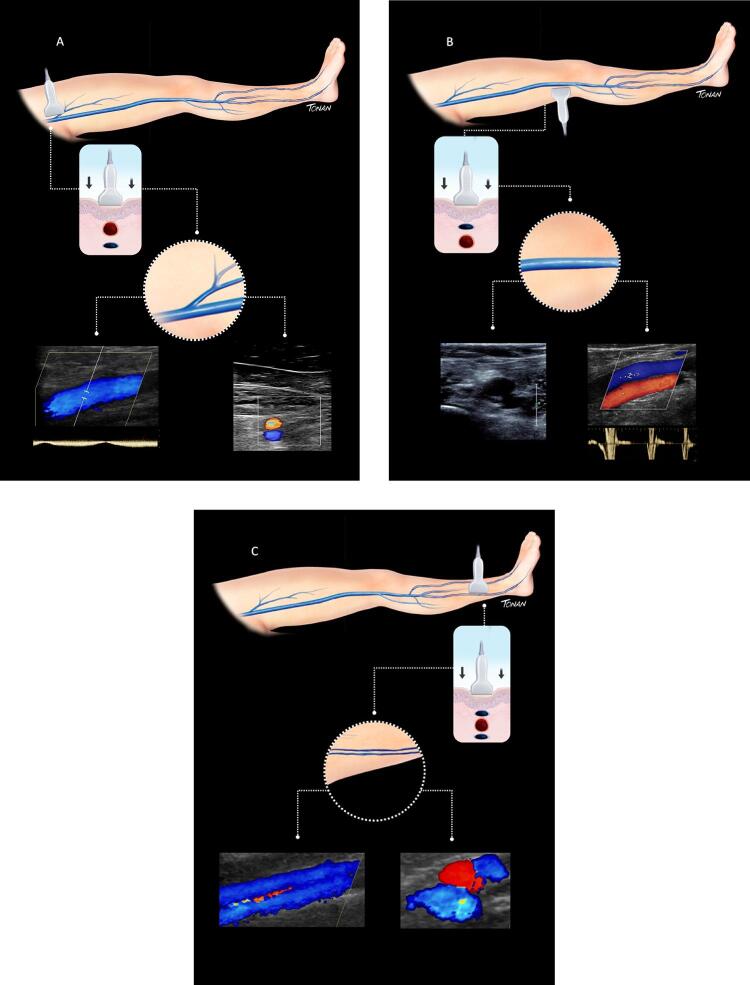
– Protocolo de exame completo de ecografia vascular das veias do membro inferior para o diagnóstico de TVP. A: desenho esquemático dos pontos de compressão em todo sistema venoso suprapatelar, e avaliação pelo mapeamento de fluxo em cores e Doppler pulsado do segmento femoral; B: desenho esquemático da compressão e avaliação pelo mapeamento de fluxo em cores e Doppler pulsado do segmento poplíteo; C: desenho esquemático de compressão e avaliação pelo mapeamento de fluxo em cores do segmento distal.

Protocolos para pesquisa de TVP ( Figuras 14 e 15 ):

**Figura 15:**
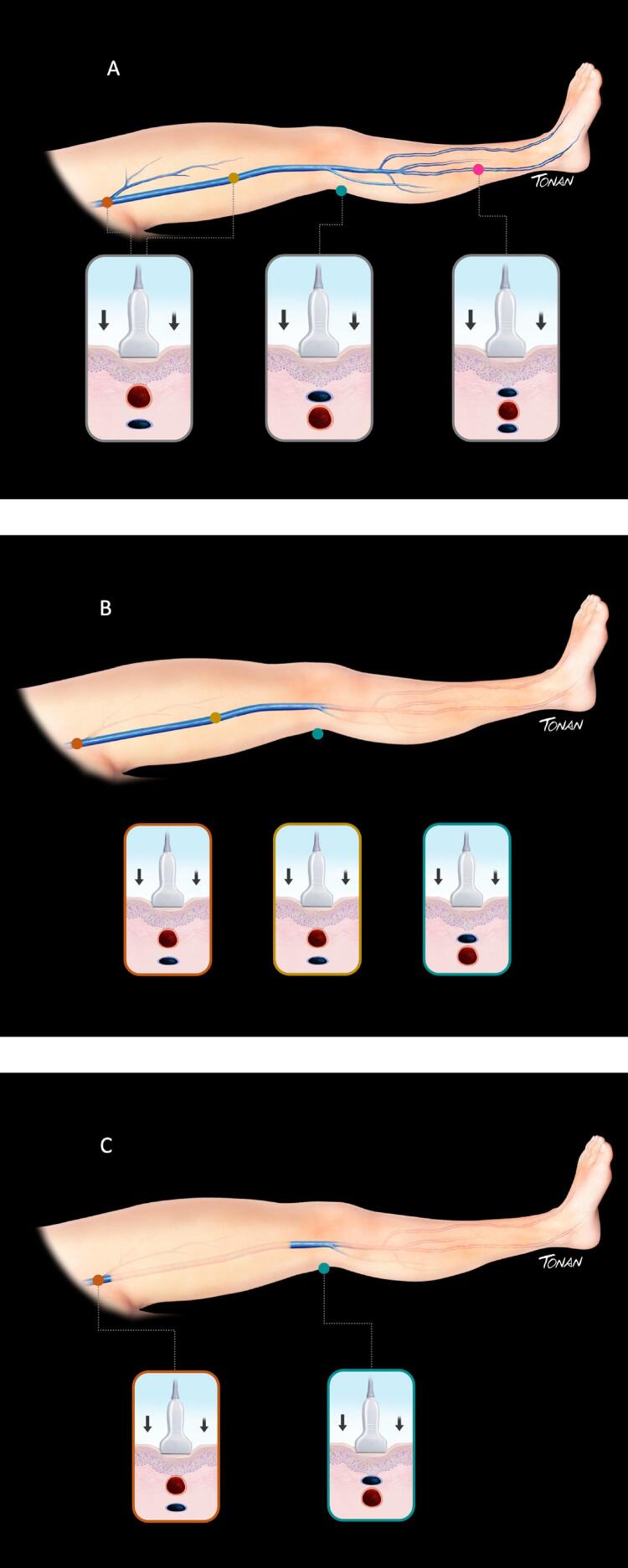
– A: protocolo de exame de ultrassom de compressão completo das veias dos membros inferiores; B: protocolo de exame de ultrassom de compressão estendido (3 pontos); C: protocolo de exame de ultrassom de compressão (2 pontos)

1. Exame completo de ultrassonografia vascular – Figura 14A-C .
2. Exame de ultrassom de compressão completo das veias dos membros inferiores – Figura 15A .
3. Exame de ultrassom de compressão estendido (3 pontos) – Figura 15B .
4. Exame de ultrassom de compressão de 2 pontos – Figura 15C .

### 3.3.1. Exame Completo de USV

Recentemente, a Sociedade Americana de Radiologia e a Sociedade Brasileira de Angiologia e Cirurgia Vascular recomendaram (classe I/nível de evidência A) a realização do exame completo de ecografia vascular para o diagnóstico de TVP aguda. Além de ser uma abordagem mais completa do paciente, a inclusão do exame do segmento infrapoplíteo permite o diagnóstico de outras doenças musculosqueléticas, que fazem parte do diagnóstico diferencial da TVP, como cisto de Baker, hematomas ou doenças musculares. ^80 , 81^ O protocolo completo constitui na utilização de todos os recursos do US para definir o diagnóstico da trombose, ou seja, avaliação da compressibilidade, calibre, aspecto ecográfico da parede e lúmen e *Doppler* colorido e espectral de todas as veias infrainguinais até o tornozelo, em todos os segmentos: veias femorais, poplítea, tibiais posteriores e fibulares ( Figura 14A-C ). De modo geral, a pesquisa de trombose venosa nas veias tibiais anteriores não está incluída nesse protocolo, por conta da raridade de seu comprometimento, sendo avaliada quando há sinais e/ou sintomas em seus trajetos. O sistema venoso superficial e muscular deve ser avaliado especialmente nas áreas sintomáticas.

Realiza-se o exame com compressões a cada 2 cm de intervalo, análise do preenchimento do vaso pelo *Doppler* colorido (usando ajustes de escalas necessários), observação da fasicidade do fluxo e análise da morfologia do *Doppler* espectral em veias femorais e poplíteas. Ainda que seja realizado de forma completa apenas o exame do membro sintomático, é relevante lembrar que a análise do *Doppler* espectral deverá ser feita em ambas as veias femorais comuns para avaliar a simetria. Quando houver assimetria no padrão do fluxo, há a necessidade de estudar as veias intrabdominais (classe I/nível de evidência A). ^91 - 94^

### 3.3.2. Exame de Ultrassom de Compressão Completo das Veias dos Membros Inferiores

Desde o ano de 1982, Steve Talbot descreveu a técnica de compressão que se tornou o exame padrão no diagnóstico de TVP. ^75^ Esse protocolo utiliza apenas a avaliação da compressibilidade de todas as veias infrainguinais até o tornozelo, em todos os segmentos ( Figura 15A ). Como mencionado anteriormente, a perda da compressibilidade da veia é o indicador mais confiável de presença de trombo.

### 3.3.3. Exame de ultrassom de compressão estendido (3 pontos)

Nesse protocolo de exame, apenas a compressibilidade é avaliada da veia femoral comum até a veia poplítea, onde há a confluência das veias da perna. Ele também é chamado de protocolo de 3 pontos, visto que avalia a compressibilidade de todas as veias proximais do membro inferior investigado ( Figura 15B ). A sensibilidade apurada nesse protocolo de exame foi significativamente maior do que no protocolo de compressão de 2 pontos (90,57% *vs.* 82,76%) com a mesma especificidade (98,52%). ^95^ Um resultado bastante semelhante foi obtido em um estudo de acurácia do protocolo de 3 pontos realizado por médicos emergencistas (91,7% de acurácia; 95% IC; 85-95,6%). ^96^ Uma metanálise recente, que incluiu 17 estudos de 16 artigos originais, comparou o protocolo de Pocus ( *Point of Care UltraSound* ) de 3 pontos com o de 2 pontos. No geral, o Pocus de 2 pontos apresentou sensibilidade combinada [0,91; IC 95%, 0,68-0,98; P = 0,86) e especificidade (0,98; IC 95%, 0,96-0,99; P = 0,60) semelhantes com o Pocus de 3 pontos (sensibilidade 0,90; IC 95% 0,83-0,95 e especificidade, 0,95; IC 95%, 0,83 -0,99). As taxas de falso-negativos do Pocus 2 pontos (4,0%) e 3 pontos (4,1%) foram quase semelhantes. A análise de metarregressão mostrou que alta sensibilidade e especificidade tendem a estar associadas ao executante inicial do Pocus e ao treinamento separado do Pocus para TVP. ^97^ (classe II / nível de evidência A).

### 3.3.4. Exame de Ultrassom de Compressão de 2 Pontos

Nesse protocolo de exame, é avaliada a compressibilidade em apenas 2 locais: na veia femoral comum 1-2 cm acima e abaixo da junção safenofemoral (na virilha); e na veia poplítea até onde houver a confluência das veias da perna ( Figura 15C ).

De modo geral, os médicos emergencistas conseguem realizar esse protocolo após treinamento, com boa reprodutibilidade, e vários são os métodos deste protocolo de pesquisa de TVP no cenário da emergência. A maioria dos treinamentos inclui aulas teóricas sobre a técnica de realização do exame, treinamento em modelos e observação de um número variável de exames. Entretanto, existem estudos que citam treinamentos de apenas 10 minutos ou menos de duas horas. Essa grande diferença entre os protocolos dificulta a comparação entre os estudos e, consequentemente, a acurácia. ^95 - 100^ Os médicos de emergência podem obter um nível de competência equivalente ao dos especialistas, mas esse processo requer treinamento e prática substanciais para alcançarem e manterem esse desempenho. Para chegarem a esse nível de excelência, precisam de treinamento regular com aplicações de ultrassom.

Um estudo comparativo entre procedimentos de 2 pontos, realizado por médicos emergencistas experientes com o exame completo, mostrou que, em 2.451 pacientes avaliados, 362 deles apresentavam TVP. O US de 2 pontos deixaria de diagnosticar 23 pacientes com TVP proximal (6,2%). ^100^ Outro estudo, controlado, randomizado e envolvendo mais de 2.000 pacientes em que foi comparado o US completo com o de 2 pontos seriado associado a dosagem do dímero D no manejo de pacientes sintomáticos para TVP atendidos na emergência, mostrou equivalência entre as duas estratégias. Entretanto, neste último estudo, os médicos eram especialistas em USV, o que indica a necessidade do protocolo deste exame ser realizado por médicos bem treinados para que seja acurado, e siga os escores clínicos de probabilidade. Na maioria das vezes, quando o paciente permanece com sintomas ou o dímero-D está elevado, o US de compressão deve ser repetido. ^101^

Por fim, o protocolo completo recomendado de exame é o destinado à pesquisa de TVP, pois se avalia toda a rede de veias profundas da perna nesse procedimento, devendo ser realizado por especialistas. Além disso, exige máquinas dedicadas de alto nível e que os pacientes sejam transferidos do pronto-socorro/enfermaria para serem avaliados.

Por outro lado, os protocolos de US de compressão de 2 e 3 pontos são abordagens simples, rápidas e de cabeceira, que não necessitam de operadores especializados ou equipamentos sofisticados. Tais metodologias são muito adequadas para serem realizadas em setores de emergência e enfermarias de hospitais, especialmente após o horário comercial e nos fins de semana, para que, juntamente à avaliação de probabilidade pré-teste, sejam capazes de conduzir inicialmente o paciente com suspeita de TVP.

A utilização dos protocolos de US de compressão de 2 pontos em pacientes críticos é uma ferramenta importante em alguns cenários, como na atual pandemia do novo coronavírus (covid-19). Existe uma relação com um estado protrombótico, o que favorece a ocorrência de fenômenos trombembólicos. Assim, diante da possibilidade clínica de TVP, a realização de um exame de US rápido e focado no membro suspeito deve ser avaliada quando o diagnóstico impactar na conduta do paciente. ^102^

## 3.4. Diagnóstico Diferencial

Os sintomas desencadeados pelo evento trombótico podem ser comuns a diversas outras condições agudas ou crônicas, como cistos sinoviais rotos, insuficiência venosa crônica, roturas e hematomas musculares. ^103^ Em outras situações, a trombose venosa pode ocorrer devido à compressão extrínseca por anormalidades vasculares como aneurismas e pseudoaneurismas, ou condições extravasculares como hematomas, abscessos, cistos sinoviais, linfadenomegalias, e tumores de linhagem neural e hematológica. ^104^ O exame ultrassonográfico completo do membro proporciona a capacidade de identificar as condições clínicas associadas e diferenciá-las das anormalidades não trombóticas. Por outro lado, um membro edemaciado, associado a estase ou diminuição da variabilidade respiratória na veia femoral, pode indicar compressão extrínseca em território pélvico. Linfadenomegalias, miomas uterinos, tumores de retroperitônio e síndrome de May-Thurner podem ser os agentes da compressão. ^105^ Nessa situação, o examinador deve chamar a atenção para os achados e sugerir essa possibilidade em seu relatório.

Linfadenomegalias, notadamente em região inguinal por doença linfoproliferativa, podem cursar com Quadro clínico de edema pronunciado no membro, tanto pelo eventual componente compressivo venoso, quanto pela insuficiência na drenagem linfática regional. Embora seja de fácil identificação, ocasionalmente a massa tumoral pode ser de tal forma volumosa que oculta a visualização da veia, dificultando a caracterização de fluxo ou mesmo a presença ou ausência de trombo. ^106^

Pseudoaneurismas, não obstante tenham características bastante definidas ao USV com fluxo colorido, podem cursar, quando trombosados, com variedade de ecogenicidade ao modo B na dependência do volume e tempo de oclusão. Quando volumosos, podem também levar à compressão venosa e estarem associados a outras condições como traumatismos fechados ou tumorações ósseas, levando à dificuldade de identificação da veia. ^107^

Um diagnóstico diferencial ultrassonográfico frequente da trombose venosa profunda é o hematoma muscular, principalmente quando localizado na musculatura da panturrilha. A apresentação clínica é muito semelhante à oclusão venosa e nem sempre é clara. Na história clínica, há referências ao traumatismo ou dor súbita. Su et al. ^108^ descrevem algumas características que podem sugerir que a lesão se trata de um hematoma, como: ecogenicidade mista, ausência de áreas anecoicas, hiperecogenicidade perilesional (sobretudo reforço no tecido subjacente) e relação entre o eixo longitudinal e transverso da lesão > 2.

**Figura 16:**
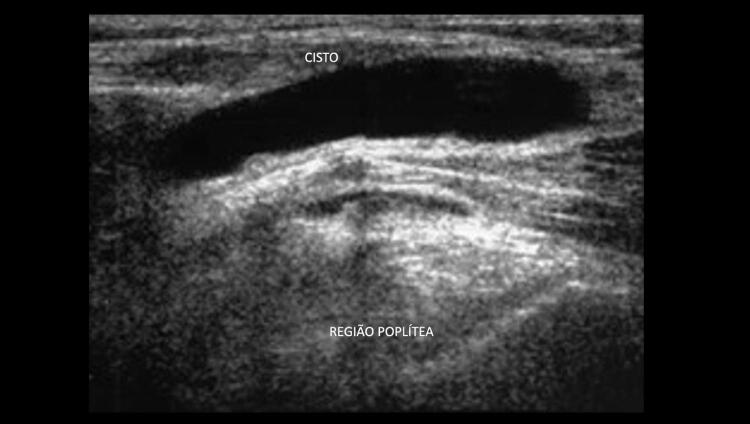
– Imagem de cisto de Baker na região posteromedial do joelho (diagnóstico diferencial da trombose venosa profunda).

Um dos achados adicionais não vasculares mais comuns nos exames venosos é o cisto sinovial poplíteo ou de Baker, mais comumente localizado na face medioposterior da fossa poplítea e que não costuma ser sintomático quanto ao sistema vascular. Entretanto, em raras condições, um cisto íntegro pode ter apresentação posterolateral, com variados graus de compressão venosa, arterial e nervosa, e apresentar sintomas neurovasculares no membro afetado. Quando há rotura do cisto de Baker em direção à panturrilha, pode cursar com Quadro clínico similar à trombose venosa aguda. Apesar da semelhança clínica, é importante ressaltar que a diferenciação entre a condição articular e a vascular deve ser feita em função do tratamento completamente distinto a ser empregado. Algumas características do cisto podem ajudar a diferenciá-lo do evento trombótico. O cisto de Baker comunica-se com a cavidade articular próxima à cabeça medial do músculo gastrocnêmico, e essa é uma característica-chave para o diagnóstico ( Figura 16 ). Apesar de seu conteúdo líquido (portanto anecoico), pode apresentar grumos em seu interior. Habitualmente, apresenta câmara única e ser multiloculado. ^109^

**Figura 17:**
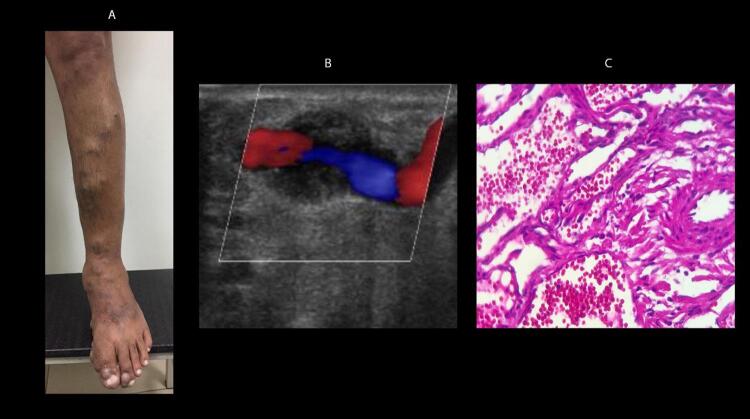
– Hemangioendotelioma venoso. A: aspecto clínico; B: imagem hipoecogênica circundando uma veia superficial à ultrassonografia com mapeamento de fluxo a cores; C: exame anatomopatológico.

Tumores de partes moles, benignos (schwanomas, neuromas histiocitomas fibrosos) ou malignos (sarcomas, osteosarcomas), podem cursar com Quadro semelhante à trombose venosa ou se apresentar como condição compressiva ( Figura 17 ). ^110^

O USV é uma ferramenta bastante acessível e disponível na abordagem inicial da pesquisa de trombose venosa; no entanto, condições não trombóticas podem cursar como diagnóstico diferencial. Em mãos experientes, o USV proporciona a possibilidade de propor diagnósticos alternativos que se apresentam com frequência nos casos suspeitos de obstrução venosa.

## 3.5. Recomendações

1. Recomenda-se a realização do exame completo de ecografia vascular para o diagnóstico de TVP aguda. (classe 1/nível de evidência A).
2. A análise do *Doppler* espectral deve ser feita em ambas as veias femorais comuns, para avaliar simetria. Quando houver assimetria no padrão do fluxo, há a necessidade de estudar as veias intra-abdominais ^91 - 94^ (classe 1/nível de evidência A).
3. Por esse consenso, recomenda-se o uso das seguintes terminologias para as anormalidades ultrassonográficas encontradas na TVP:
  1. Aguda/recente;
  2. Crônica/antiga;
  3. Predominância de alterações agudas;
  4. Predominância de alterações crônicas.

O termo “indeterminado” poderia ser usado nas poucas ocasiões em que, de fato, não foi possível avaliar o tempo do evento.

## 3.6. Como Descrever o Laudo do Exame

**Corpo do laudo:** primeiramente, é fundamental informar a qualidade técnica do exame, em quais condições foi realizado (se em caráter de urgência ou não), se o paciente pode cooperar no posicionamento adequado, se foi detectado edema importante associado ou qualquer fator que impediu uma qualidade técnica adequada.

Descrever separadamente a avaliação das veias, sobretudo quando houver trombose venosa, descrevendo todos os aspectos ultrassonográficos do exame: modo B e *Doppler* (que levam a concluir sobre a presença ou não de trombose), tempo de evolução clínica e se apresenta aspecto agudo, indeterminado ou alterações crônicas pós-trombóticas.

Aspectos a serem descritos no corpo do laudo sobre as veias:

1. Calibre (comparar com a contralateral e/ou com o calibre da artéria adjacente) e aspecto da parede venosa em todos os segmentos avaliados;
2. Compressibilidade parcial ou total, especificando o segmento acometido;
3. Presença ou não de material intraluminal. Quando presente, avaliar ecogenicidade, ocupação total ou parcial da luz da veia e se tem ou não componente móvel;
4. Presença ou não de colaterais;
5. Sobre o fluxo:
*- Color* *Doppler* : presente ou ausente e se ocupa total ou parcialmente a luz da veia;
  - Análise espectral: fluxo pode ser ausente. Quando presente, informar a fasicidade com a respiração ou contínuo, se aumenta (ou não) compressão distal e/ou (ou não) refluxo associado.
6. Descrever a presença de outros possíveis diagnósticos diferenciais, quando houver: cisto de Baker, tumores, sinais sugestivos de linfedema e imagens sugestivas de hematoma.

**Conclusão:** Na conclusão, relatar se há ou não sinais de trombose. Em caso positivo, informar se ela é total ou parcialmente oclusiva e se a(s) veia(s) e os segmentos venosos estão comprometidos. Determinar se a trombose é aguda/recente ou apresenta alterações pós-trombóticas crônicas/antigas. No caso de trombose clinicamente subaguda (2 semanas a 6 meses), recomendam-se as expressões: sinais predominantes de trombose aguda ou predominantes de trombose crônica. Nos casos de tromboses não oclusivas, descrever se apresenta (ou não) refluxo associado. Os aspectos descritos no laudo para o fundamento da conclusão estão resumidos no Quadro 10 .

**Quadro 10:** – Aspectos a serem descritos no laudo e que fundamentam a conclusão

SINAIS AO ULTRASSOM	TEMPO DE EVOLUÇÃO CLÍNICA	GRAU DE COMPRESSIBILIDADE	ECOGENICIDADE DO TROMBO	DIÂMETRO DA VEIA	TIPO DE FLUXO
Trombose aguda / recente	Inferior a 15 dias	Total ou parcialmente incompressível	Hipoecogênico ou ecolucente	Aumentado	Ausente / Fasicidade reduzida ou contínuo
Trombose Crônica / antiga	Acima de 6 meses	Total ou parcialmente incompressível	Hiperecogênico / trabeculações fibróticas residuais	Reduzido ou Normal	Contínuo / ausente / permeado com as alterações residuais
Trombose com predominância de sinais de trombose aguda ou predominância de sinais de trombose crônica, ou excepcionalmente, indeterminada	Clinicamente Subagudo: Entre 2 semanas e 6 meses	Total ou parcialmente incompressível	Isoecogênico / ecogenicidade mista / predominantemente hipoecogênico / predominantemente hiperecogênico	Normal ou pouco modificado	Fasicidade reduzida / Contínuo / ausente

## 4. Ultrassonografia Vascular no Diagnóstico da recorrência de trombose venosa profunda e “síndrome pós-trombótica”

### 4.1. Recorrência de trombose venosa (RTV)

Refere-se à ocorrência de um novo episódio de trombembolismo venoso em indivíduo com história de evento prévio. Em se tratando de trombose de membros inferiores, a recorrência da trombose pode se manifestar por trombo em nova localização (propagação para segmento proximal ou distal ao evento inicial, segmento diferente do mesmo membro, membro diferente) ou em segmento previamente acometido. ^111^ Na pratica médica atual, recomenda-se que o paciente diagnosticado com um primeiro episódio de TVP seja tratado com algum anticoagulante por um período de 3 a 6 meses, ^112^ o que na maioria das vezes resulta na resolução dos sintomas, por conta da recanalização da veia ou surgimento de circulação colateral.

A recorrência de trombose é um evento comum. Estima-se que após a descontinuação do anticoagulante, a taxa de recorrência aumente progressivamente ao longo do tempo, chegando a 40% entre todos os pacientes no período de 10 anos (incidência cumulativa: 7,2% em 6 meses, 11% em 1 ano, 19,6% em 3 anos, 29,1% em 5 anos, 34,3% em 8 anos, e 39,9% em 10 anos). ^113^

O fator de risco mais importante para recorrência é a causa do primeiro episódio da TVP. Em pacientes com TVP provocada por um fator de risco transitório (p. ex., após uma grande cirurgia), o risco anual é de apenas 1%, podendo-se cogitar um tratamento limitado a 3 meses na dependência de outros fatores de risco. ^114^ Quando não há um fator causal principal identificável, ou quando há apenas uma causa mínima transitória (trombose “não provocada”/idiopática), o que ocorre em cerca da metade dos casos de TVP, a prevalência de recorrência aumenta para 5%-10% em 1 ano e até 30% em 5 anos, ^115^ o que justifica a continuidade do tratamento com antagonistas da vitamina K (AVK) ou novos anticoagulantes (NOACS) por 6 meses ou mais, visto que estes reduzem significativamente o risco de recorrência em 80 a 90%. ^116^

Primariamente, a recorrência após o término da anticoagulação depende de dois fatores: primeiro, se a TVP aguda foi tratada efetivamente, incluindo a duração mínima do tratamento anticoagulante; e, segundo, a presença de fatores de risco intrínsecos que, de fato, aumentam a recorrência, notadamente a doença maligna e as trombofilias. ^117^ A duração ideal do tratamento anticoagulante é discutida até hoje, sendo embasada na extensão da trombose (proximal ou distal) e na causa do evento trombótico, quando relacionada a um fator principal predisponente. Adotamos as recomendações do American College of Chest Physicians (ACCP). ^112^

Além da causa precipitante da TVP, outros fatores são importantes para determinar o risco de recorrência. O ultrassonografista deve estar alerta aos fatores clínicos que estão associados a recorrência da trombose, como: TVP não provocada RR 2,3 (IC 95%; 1,8-2,9), obesidade RR 1,6 (IC 95%; 1,1-2,4), sexo masculino RR 2,8 (IC 95%; 1,4-5,7), dímero D positivo RR 2,6 (IC 95%; 1,9-3,5), trombose residual RR 1,5 (IC 95%; 1,1-2,0), trombofilia hereditária RR 1,5 (IC 95%; 1,1-1,9), doença intestinal inflamatória RR 2,5 (IC 95%; 1,4-4,2), anticorpo antifosfolipídio RR 2,4 (IC 95%; 1,3-4,1) e neoplasia maligna. ^117^

A doença maligna é um dos principais fatores de risco tanto para trombose quanto para recorrência de TVP. Pacientes com câncer apresentam risco 2 a 4 vezes maior para RTV, chegando a 4,2 vezes nos indivíduos em quimioterapia, ^118^ procedimento que afeta o endotélio vascular e a cascata de coagulação, o que libera substâncias pró-trombóticas durante a lise do tumor.

Deter conhecimentos sobre a história natural da TVP é importante para determinar a terapêutica, e, no caso da USV, para o seguimento dos pacientes. A recanalização é um processo dinâmico, que depende das forças líticas ou pró-coagulantes, começa na primeira semana após o episódio de TVP e continua por meses, sendo que a maior parte da massa trombótica é reduzida nos primeiros 3 meses. ^119^ Demonstrou-se nos pacientes seguidos com ultrassonografia que 56% apresentaram resolução completa, ^120^ sendo que esta taxa é diferente para as tromboses próximas e distais. Nas tromboses distais, é mais frequente uma recanalização total e sem sequelas como refluxo significativo ou obstrução. Por outro lado, nas tromboses próximas ou nas que envolvem os dois segmentos, a prevalência de refluxo ou obstrução é bem mais significativa. ^121^

A taxa de recanalização guarda relação com a prevalência de refluxo no sistema venoso. Quanto mais rápida a recanalização, maior é a associação com valvas competentes. ^120 , 122^ O refluxo, associado ou não a obstrução venosa decido a recanalização incompleta, pode provocar hipertensão venosa persistente, levando ao aparecimento dos sinais de hipertensão venosa crônica, que são chamados de síndrome pós-trombótica (SPT).

Apesar de vários estudos ressaltarem a importância da trombose venosa residual, que é essencialmente diagnosticada em exames de USV em seguimento dos pacientes com TVP, ^123 - 125^ uma metanálise recente, englobando 14 estudos, mostrou apenas um leve aumento do risco de recorrência (HR,1,5; 95% CI, 1,1-2,0) em pacientes com trombose venosa residual. ^126^

### 4.2. Diagnóstico Ultrassonográfico da Recorrência de Trombose

Não existem parâmetros isolados pela USV que caracterizem a recorrência de trombose, ou modelos clínicos validados que permitam o diagnóstico, que é feito levando-se em conta o reaparecimento dos sintomas e achados clínicos de TVP, mediante a probabilidade em face aos fatores de risco e na presença de achados ultrassonográficos que sugiram a recorrência da trombose.

Os achados comparativos entre o exame da atual suspeita de recorrência com o exame “base”, ao término do tratamento, são a única forma validada para o diagnóstico de RTV. Dessa forma, é de suma importância a existência de exames prévios que descrevam de maneira clara e precisa a extensão do acometimento e o grau de recanalização da trombose prévia.

Os achados ultrassonográficos são similares aos descritos para o diagnóstico da primeira trombose, dificultados pela presença de massa trombótica antiga que pode confundir o diagnóstico. Tais achados estão relacionados no Quadro 9 desse documento.

O diagnóstico pode ser fácil quando a recorrência da trombose ocorrer no membro contralateral ou em um segmento novo, claramente não envolvido na trombose venosa anterior. Quando ocorre em um segmento anteriormente envolvido, um aumento de 2 mm no diâmetro da veia sob compressão do segmento previamente trombosado foi relatado como diagnóstico de recorrência de TVP. ^127 , 128^ Por conta do aumento de 2 mm com a compressão máxima ter um valor preditivo positivo baixo, outros autores propuseram a utilização de um aumento de diâmetro ≥4mm para o diagnóstico de RTV. ^129^ Um estudo recente que revisou 36 artigos publicados sobre o tema demonstra que uma nova veia não compressível ou um aumento no diâmetro de um segmento de veia previamente trombosada ≥4mm são suficientes para confirmar o diagnóstico de recorrência de TVP. O aumento de diâmetro menor que 2mm permite que a recorrência seja descartada, e o aumento entre 2 e 4 mm seria considerado duvidoso. ^130^

A ecogenicidade do trombo, mesmo quando correlacionada com a organização do trombo *in vitro* , torna subjetiva a avaliação *in vivo* . Importante ressaltar que mesmo trombos agudos podem demonstrar diferentes estágios de organização. ^131^

La Gal et al. ^132^ demonstraram que a estratégia diagnóstica de comparação do exame, na atual suspeita de recorrência com um exame “base” ao término do tratamento, pode ser seguramente utilizada no descarte da recorrência de TVP.

Tais considerações reforçam a necessidade de um exame minucioso ao final do tratamento para servir de base comparativa com outros procedimentos subsequentes, descrevendo todos os achados, inclusive o diâmetro residual após compressão máxima, bem como o local em que a medida foi realizada (convém realizar no local de maior massa trombótica residual).

### 4.3. Síndrome Pós-trombótica

A síndrome pós-trombótica (SPT) é uma condição crônica caracterizada por sinais e sintomas que se desenvolvem como consequência de TVP prévia, cuja complicação mais comum surge a longo prazo e acomete em torno de 20-40% dos casos de TVP nos membros inferiores, mesmo na vigência de anticoagulação adequada. ^133^

A SPT causa um significativo impacto na qualidade de vida dos pacientes acometidos, comprometendo e limitando suas atividades diárias e a sua produtividade. Tal impacto pode ser comparado ao câncer e às doenças cardíacas, além de provocar um expressivo aumento de custos ao sistema de saúde. Dados de estudos suecos demonstram custos em torno de 75% maiores que a TVP primária. ^133^

#### 4.3.1. Fisiopatologia

A fisiopatologia da SPT é uma combinação entre fenômenos obstrutivos e o refluxo venoso por insuficiência valvular, que determinam como resultado a hipertensão venosa. O aumento da pressão venosa nos capilares subcutâneos e na microcirculação culmina em incompetência valvular de veias perfurantes. Na maioria dos casos, o processo obstrutivo caracteriza-se por uma recanalização que ocorre nos primeiros 6-12 meses após o evento agudo, o que leva a uma combinação de obstrução parcial e variados graus de refluxo dos segmentos acometidos. Todas as alterações resultam, em última instância, em destruição de válvulas venosas, desenvolvimento de válvulas colaterais em locais em que persiste um grau maior de obstrução e maior tendência de recorrência de episódios agudos. ^133 , 134^

Um sistema de veias safenas competentes pode atuar favoravelmente na drenagem venosa nos casos em que há um significativo componente obstrutivo. Por outro lado, quando há insuficiência de veias safenas preexistentes, o retorno venoso se tornará ainda mais comprometido, podendo a condição clínica destes pacientes se deteriorar mais rapidamente.

O processo inflamatório é o principal fator na SPT e está presente na resolução da trombose. A fibrinólise, a organização do trombo e a neovascularização envolvem a interleucina-6 e a adesão intercelular de mólecula-1, o que causa danos valvares nos primeiros meses após a fase aguda da TVP. Prandoni e Kahn sugerem que a ausência de recanalização neste período é um importante preditor para a SPT. ^133 , 135 , 136^

#### 4.3.2. Quadro Clínico

Apresenta-se com um largo espectro de manifestações, que passam por sinais clínicos leves até sintomas graves como dor crônica no membro acometido (limitante para as atividades diárias), edema intratável e surgimento de úlceras. Outros sinais podem aparecer, como hiperemia, hiperpigmentação, dilatações venosas e lipodermatoesclerose, além de sintomas como dor, sensação de peso, parestesia, prurido e cãibra. Em casos mais avançados, podem ocorrer infecções de pele, do subcutâneo e do sistema linfático.

O grau do componente obstrutivo luminal residual incide diretamente na gravidade da repercussão dessas manifestações clínicas, levando à dor acentuada e à claudicação venosa.

Os fatores de risco que podem contribuir para o desenvolvimento da SPT são:

- Obesidade;
- Veias varicosas;
- Trombose proximal e recorrência;
- Uso inadequado da anticoagulação oral;
- Tempo da resolução da TVP: quanto menor e mais lenta, maior a probabilidade de SPT;
- Idade e gênero (apresentam estudos contraditórios);
- Outros potenciais fatores, ainda não comprovados: malignidade, imobilização, cirurgia, gravidez e trombofilia.

#### 4.3.3. Diagnóstico

Essa condição deve ser diagnosticada após a finalização do tratamento do episódio da TVP, ou seja, não pode ser caracterizada como SPT durante os primeiros três meses. ^133^

O arsenal diagnóstico para SPT deve ser composto por clínica, laboratório, imagem e pletismografia a ar. Não existe um método diagnóstico referencial, laboratorial, de imagem ou fundamentado em testes funcionais para SPT. Entretanto, é fundamental que seja registrada por imagem a presença de obstrução e refluxo venoso, e mesmo de forma não quantitativa deve-se tentar informar qual dos dois componentes fisiopatológicos predomina.

A classificação clínica, etiológica, anatômica, patológica (CEAP) e o escore de severidade clínica venosa (ESCV), utilizados para a insuficiência venosa crônica (IVC), podem ser aplicados de forma apropriada para quantificar com várias medidas a SPT. ^133 , 137 - 139^

A escala de *Brandjes* , as medidas de *Ginsberg* e a escala de *Villalta* foram especificamente desenvolvidas para auxilar no diagnóstico da SPT, mas infelizmente não tem sido adotadas como comparativos em outros estudos. No congresso da Sociedade Internacional de Trombose e Hemostasia de 2008, em Viena, passou-se a recomendar a escala de Villalta para a definição e presença de SPT, através da qual o escore >5 indica o diagnóstico da síndrome. A presença de úlcera torna o escore ainda mais grave ( Quadro 11 ). ^140 , 141^

**Quadro 11:** – Escala de Villalta para síndrome pós-trombótica 141

SINTOMAS	NORMAL	DISCRETO	MODERADO	ACENTUADO
Dor	0	1	2	3
Cãibras	0	1	2	3
Peso	0	1	2	3
Parestesia	0	1	2	3
Prurido	0	1	2	3
Sinais	0	1	2	3
Edema pretibial	0	1	2	3
Pele endurecida	0	1	2	3
Hiperpigmentação	0	1	2	3
Vermelhidão	0	1	2	3
Ectasia venosa	0	1	2	3
Dor durante a compressão da panturrilha	0	1	2	3
Úlcera venosa	0	1	2	3

O método de imagem de escolha é a USV, por conta de baixo custo, disponibilidade em pequenos centros e alta acurácia. Em pacientes com apresentação clínica sugestiva de SPT, mas sem história de TVP, a ultrassonografia é indicada para rastreio de evidências de TVP prévia.

**Figura 18:**
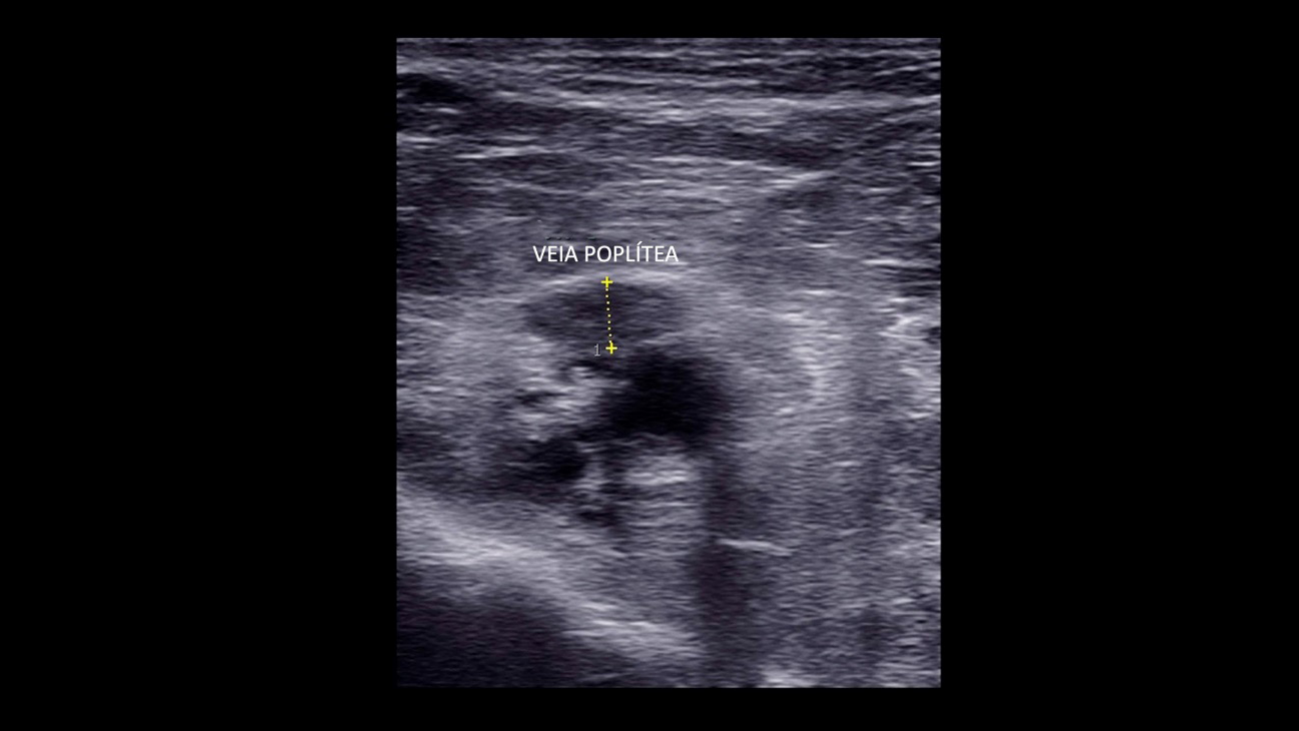
– Imagem ultrassonográfica bidimensional, com medida do diâmetro da veia poplítea após manobra de compressão.

**Figura 19:**
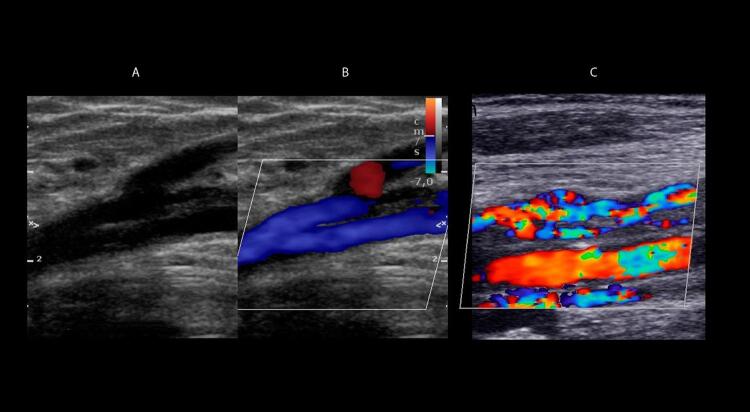
– A: imagem bidimensional em corte longitudinal de veia femoral comum com espessamento parietal e trabeculações/traves no interior do vaso. B: mapeamento de fluxo em cores, evidenciando fluxo ao redor da trabeculação. C: achados semelhantes em veia poplítea.

A USV possibilita informar localização, medidas dos diâmetros da veia após a manobra de compressão ( Figura 18 ), e, de maneira mais subjetiva, o grau de componente obstrutivo luminal nas veias acometidas, danos valvares e qualificação do refluxo ( Figuras 19 e 20 ). Embora pouco utilizada e pouco disponível, a pletismografia a ar mostra-se eficaz na quantificação do refluxo venoso e se presta para o controle evolutivo da recanalização e diagnóstico de retrombose.

**Figura 20:**
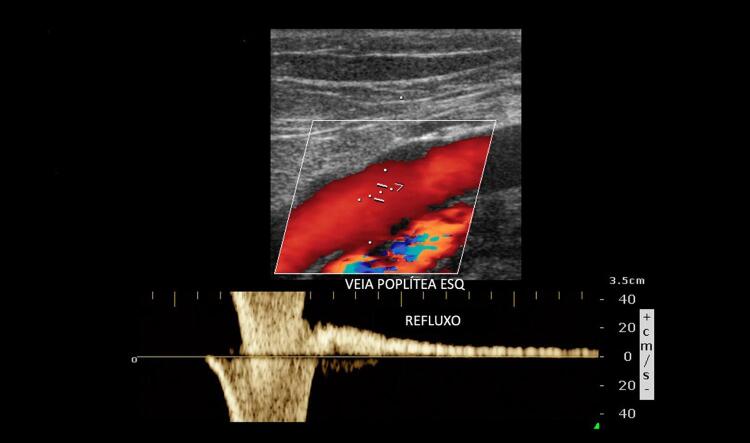
– Refluxo ao mapeamento de fluxo a cores (em vermelho) e ao Doppler pulsado em veia poplítea.

Além de definir o diagnóstico, a USV também se presta ao planejamento da terapêutica. Destaca-se tanto na avaliação do comprometimento do segmento cavoilíaco para abordagem endovascular e reestabelecimento de sua perviedade quanto para o seguimento pós-tratamento, sendo imprescindível no diagnóstico de recorrência da trombose. ^137 , 142^

Em casos que apresentem limitações para a USV, como a presença de edema acentuado e lipodermatoesclerose, que muitas vezes apresenta nódulos focais de fibrocalcificação, recomenda-se a adoção de algumas estratégias para melhor aquisição das imagens.

Mudanças de posição do transdutor e ângulo de insonação do feixe de US, redução na frequência do transdutor, mudança para o modo trapezoidal no caso do transdutor linear são recomendadas, e, caso necessário, efetuar troca de transdutores por aqueles com frequência ainda mais baixa, que possibilitam maior penetração, como é o caso dos transdutores convexos, setoriais e endocavitários.

Pacientes portadores de SPT devem ser examinados em decúbito dorsal e ortostatismo, posição em que é possível visibilizar melhor alterações luminais como espessamentos e trabéculas, além de promover uma pesquisa de refluxo mais eficaz ( Figuras 19A-C e 20 ). As veias do segmento infragenicular podem também ser avaliadas com o paciente sentado e de pés levemente apoiados sobre um tablado, mantendo a musculatura mais relaxada, facilitando a insonação de veias mais profundas.

No segmento cavoilíaco, deve-se não apenas avaliar a perviedade, mas também procurar por sinais de compressão extrínseca, com destaque para a compressão da veia ilíaca comum esquerda em seu trajeto entre a artéria ilíaca comum direta e o corpo vertebral adjacente, usualmente L5.

Os principais achados à ultrassonografia são a redução do diâmetro venoso e as alterações luminais crônicas, como espessamento parietal e trabéculas hiperecoicas, além da insuficiência valvular. Outro achado menos frequente é a presença de fluxo com padrão de fístula arteriovenosa em pontos focais de segmentos venosos acometidos por trombose, sem repercussão em veias e artérias axiais adjacentes e de significado clínico desconhecido. ^142^

## 5. Protocolos de Seguimento com a Ultrassonografia Vascular após a Trombose Venosa Profunda

### 5.1. Introdução

A TVP é um processo dinâmico com períodos de recanalização, progressão e recorrência, e a USV mostra-se uma importante ferramenta para o diagnóstico e o seguimento da TVP. Os aspectos ultrassonográficos têm um papel importante no desfecho dessa doença, possibilitando seu estudo em diferentes estágios que serão descritos a seguir.

### 5.2. Recanalização

**Figura 21:**
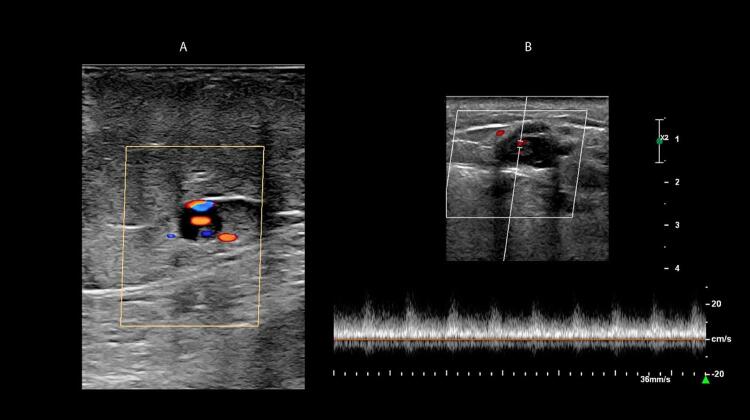
– Processo de neovascularização na recanalização da trombose. A: corte transverso de veia com imagens ecogênicas correspondentes ao trombo no interior do vaso. O mapeamento em cores demonstra fluxo no interior do trombo. B: Doppler pulsado, detectando fluxo no interior do trombo.

A recanalização da trombose venosa é um processo complexo, envolvendo mecanismos de fibrinólise intrínseca e extrínseca, fragmentação periférica, neovascularização e retração do trombo. ^134 , 143 , 144^ Tais dados têm sido confirmados por estudos por meio do uso da USV, que demonstraram a regressão do trombo nos três primeiros meses, podendo ocorrer recanalização completa na metade dos pacientes dentro de 6 a 9 meses após o episódio de TVP. ^8 , 145^ A recanalização parcial e a persistência do segmento ocluído ocorrem em cerca de 20% e 5% dos casos, respectivamente ^146^ ( Figura 21A ).

A observação de imagens de pequenos vasos tortuosos identificados pelo mapeamento de fluxo em cores e *Doppler* pulsado, no interior do trombo e adjacente à parede venosa, sugerem a presença de neovascularização no complexo processo de recanalização ^134 , 143^ ( Figura 21B ).

### 5.3. Recorrência da Trombose

Apesar de a evolução para o processo de recanalização ser importante, a recorrência de eventos trombóticos não é incomum. A taxa de recorrência de trombembolismo sintomático varia entre 5% a 13% dos casos, dependendo do tempo de seguimento realizado com o USV. Entretanto, os eventos trombóticos sem manifestações clínicas que são identificados pelo USV tendem a ser maiores. ^8^

O diagnóstico de recorrência da trombose é bastante dependente do exame ultrassonográfico realizado durante o primeiro episódio. São critérios sugestivos de recorrência:

1. Identificação de novos sítios de trombose;
2. Oclusão de segmentos venosos parcialmente recanalizados registrados em exame prévio;
3. Aumento maior que 4mm no diâmetro de segmentos venosos parcialmente recanalizados registrados em exames prévios ^111 , 130^ ( Figura 22 ).
  **Figura 22:**
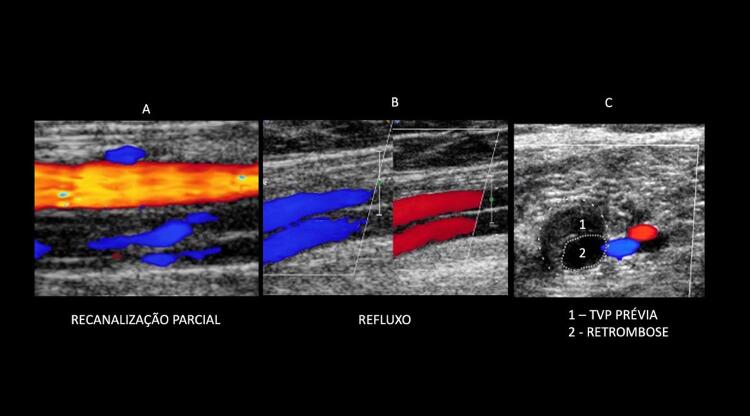
– Alterações ultrassonográficas que podem ser encontradas no seguimento do paciente com trombose venosa profunda. A: corte longitudinal da artéria femoral (em vermelho) e veia femoral (em azul). Presença de recanalização parcial do segmento venoso circundando traves residuais. B: corte longitudinal da veia femoral. Fluxo venoso ascendente normal é detectado em azul pelo mapeamento de fluxo em cores, e refluxo em vermelho, após a manobra de compressão distal. C: corte transversal de veia muscular em panturrilha, com diâmetros aumentados e compressibilidade reduzida. Em 1, observa-se imagem ecogênica referente a TVP prévia, e em 2 observa-se imagem hipoecoica referente ao processo de recorrência atual. Alguns fatores associados à persistência da alteração pós-trombótica, como presença de trombos residuais e/ou insuficiência valvular pós-flebítica, foram considerados preditores de SPT. No entanto, tais achados podem ser utilizados para alterar o planejamento terapêutico ainda são temas controversos na literatura. 148,149

### 5.4. Insuficiência Valvular

O refluxo secundário à TVP, resultante do dano valvar, é descrito em cerca de 33% a 59% dos segmentos venosos acometidos ( >Figura 22B ). Sabe-se que a veia mais frequentemente acometida é a poplítea, seguida da femoral. ^146^ A avaliação do tempo de refluxo com as manobras de compressão distal é considerada anormal quando superior 1.000 ms nas veias femoral comum, femoral e poplítea, e igual ou superior a 500ms nas demais veias. ^147^

Alguns fatores associados à persistência da alteração pós-trombótica, como presença de trombos residuais e/ou insuficiência valvular pós-flebítica, foram considerados preditores de SPT. No entanto, tais achados podem ser utilizados para alterar o planejamento terapêutico ainda são temas controversos na literatura. ^148 , 149^

### 5.5. Discussão

#### 5.5.1. Primeiro USV Negativo

De acordo com recente recomendação da Sociedade Americana de Radiologia e Ultrassonografia, orienta-se o cumprimento completo do protocolo para diagnosticar TVP pela USV. ^80^ Também orienta-se para que seja realizado um exame ultrassonográfico completo entre o período de 5 dias a 1 semana após o primeiro ultrassom completo ou *point of care* negativos nas seguintes situações:

- Quando houver persistência ou piora dos sintomas;
- Em pacientes de alto risco para TVP, cuja etiologia dos sintomas não foi elucidada;
- Depois de exames anteriores tecnicamente prejudicados.

#### 5.5.2. Trombose Proximal x Distal

Discute-se na literatura o protocolo de seguimento com USV para os pacientes em tratamento de TVP proximal, ou seja, nos territórios ilíaco, femoral e poplíteo. Não está claro se os pacientes em uso de medicação anticoagulante adequada obtiveram benefícios com a realização do USV durante o tratamento ou, ainda, se as conclusões de um exame repetido alterariam a estratégia do tratamento estabelecido. Entretanto, demostrou-se que a realização do USV ao final do tratamento é importante para basear avaliações futuras, sendo crucial determinar as veias que foram recanalizadas e quais ainda apresentam alterações pós-trombóticas. ^80 , 123 , 150 - 152^

No que diz respeito à trombose isolada das veias da perna, classificada como axial quando envolve as veias tibiais e/ou fibulares, e musculares quando envolve apenas as veias musculares, em alguns centros a pesquisa da TVP pelo USV em casos suspeitos não inclui seu estudo. Com essa conduta inicial, recomenda-se o seguimento do paciente para um segundo exame no sétimo dia após o surgimento dos sintomas. A indicação desse segundo procedimento serve para avaliar a possibilidade do envolvimento do segmento proximal, caso tenha ocorrido propagação da TVP distal não diagnosticada na fase aguda. Quando o resultado deste segundo exame for normal, afasta-se a possibilidade de trombose e o paciente não é anticoagulado. Nesses centros, o risco de embolia pulmonar estimado ao final de 3 meses é relatado em 0,5% (95% CI, 0,4%-0,9%) dos casos. ^18 , 153 , 154^

Aconselha-se o protocolo de seguimento seriado com USV, dada a baixa taxa de propagação proximal da trombose da veia poplítea, que varia de 3 a 15% na literatura ^155 - 158^ , e apresenta poucos benefícios advindos da anticoagulação. Masuda et al., ^159^ em uma revisão de metanálise, reforçam essa baixa taxa de propagação. Schwartz et al., ^160^ em estudo randomizado, demonstraram não haver diferença estatística significante entre os pacientes tratados com anticoagulação por 10 dias e os indivíduos sem tratamento (3,7% *versus* 3,8%), sendo que a taxa de propagação descrita por esses autores para a veia poplítea foi de 1,9%.

Poucos estudos na literatura versam sobre a recorrência na TVP distal. Masuda et al. ^159^ não encontraram publicações robustas para comparar os pacientes tratados com anticoagulação ou apenas acompanhados por USV. Lagerstedt et al. ^161^ estudaram pacientes com TVP distal sintomática e medicados com varfarina por 5 dias em todos os pacientes. Após esse período, os pacientes foram randomizados para manutenção da varfarina por 3 meses ou suspensão do tratamento. Após 3 meses, a taxa de recorrência da TVP foi de 29% x 0% nos grupos “tratado” *versus* “não tratado”, respectivamente.

Nos casos de TVP distal sintomática, opta-se por tratamento com anticoagulação. A maioria dos autores sugere que seja curto, pelo período máximo de 6 semanas em pacientes com baixo risco. ^160 , 162^ Para os casos de alto risco (câncer, TVP prévia, envolvimento de duas ou mais veias, idade maior que 50 anos e trombofilia) recomenda-se o tratamento por 12 semanas. ^163^

Os centros que utilizam o protocolo com USV completo optam por tratar o paciente independente de a trombose ser proximal ou distal. O risco estimado de embolia pulmonar com essa conduta, ao final de três meses, também é baixo, de 0,6% (95% CI, 0,3%-0,9%). ^101 , 164^

As alterações pós-trombóticas, utilizadas como parâmetros para orientar a duração da anticoagulação, são pouco robustas. São necessárias mais pesquisas nesse campo. ^123 , 165^

Estudo realizado em pacientes com TVP proximal e uso de rivaroxabana avaliou as alterações ultrassonográficas encontradas no primeiro, terceiro, no sexto e no décimo segundo meses, e a taxa de recanalização das veias acometidas. ^166^ Outros autores ^167^ ressaltam a ligação entre a presença de refluxo na veia poplítea, a permanência de traves fibróticas e o desenvolvimento de SPT. Sartori et al. ^168^ acompanharam 172 pacientes com trombose isolada de perna em tratamento com enoxaparina por 6 semanas e avaliaram o grau de recanalização pela USV. O exame foi realizado durante o diagnóstico e no término do tratamento. Aproximadamente metade destes pacientes (49,5%) apresentaram recanalização das veias, e não houve significância estatística entre recanalização e tamanho do trombo ou sítio anatômico de acometimento (veias axiais *versus* musculares).

#### 5.5.3. Recorrência de Trombose Venosa Profunda

Após um episódio inicial de TVP, a recorrência pode ocorrer em cerca de 25% dos pacientes em 5 anos e ser responsável pelo aumento do risco de embolia pulmonar e síndrome pós-trombótica. ^169^ O diagnóstico da TVP recorrente é mais complexo porque, ao contrário da abordagem do primeiro episódio de TVP, em que temos algoritmos validados de probabilidade pré-teste associado ao uso do dímero D e da ultrassonografia vascular (USV), ^19^ tais critérios não estão validados em relação à recorrência. ^170^ O achado da ausência de compressibilidade pela USV de um segmento anteriormente não afetado pode ser considerado diagnóstico. ^152^

Às vezes, o trombo inicial não se resolve completamente, resultando em alterações pós-trombóticas crônicas, o que dificulta o diagnóstico. Nesse caso, o diagnóstico da TVP recorrente em um segmento venoso previamente afetado pode ser feito por meio da observação do aumento no diâmetro do trombo em pelo menos 4 mm quando comparado a um estudo prévio. Entretanto, a acurácia desse achado é controvérsia nos, ^171^ e o diagnóstico depende de um exame prévio de qualidade e com número adequado de imagens para comparação, associado a medidas dos calibres nos diversos segmentos venosos afetados, o que não ocorre na prática clínica diária. Além disso, esse critério não é usado nos episódios prévios de trombose de panturrilha, o que dificulta ainda mais a abordagem dos pacientes.

O acompanhamento dos pacientes com TVP recorrente deve seguir os critérios adotados durante o episódio inicial pela escassez de estudos que avaliem especificamente o seguimento desse grupo. Enfatizamos ainda a importância de um estudo completo no término do tratamento, ou a realização de um exame de acompanhamento após seis meses nos casos de anticoagulação prolongada, incluindo a investigação da presença de alterações pós-trombóticas crônicas com uma avaliação da presença de insuficiência venosa em ortostatismo.

## 5.4. Recomendações

Com base nos dados de literatura e na discussão de especialistas da diretriz, sugerimos:

1. **USV inicial negativo:** realizar exame ultrassonográfico completo entre 5 dias a 1 semana após um primeiro ultrassom completo ou *point of care* negativos nas seguintes situações: (a) persistência ou piora dos sintomas, (b) em pacientes de alto risco para TVP cuja etiologia dos sintomas não foi elucidada, e (c) com exames anteriores tecnicamente prejudicados.
2. **USV inicial com diagnóstico de TVP proximal (território ilíaco, femoral e poplíteo):** repetir o exame ultrassonográfico completo próximo ao término do tratamento para avaliação do grau de recanalização e presença de alterações pós-trombóticas. Ou ainda, em qualquer momento no qual o paciente apresente sintomas no decorrer do tratamento, para avaliação de recorrência ou extensão da trombose inicial.
3. **USV inicial com diagnóstico de TVP distal (veias tibiais, fibulares ou musculares):** repetir o exame ultrassonográfico em 6 semanas ou em 12 semanas do início do Quadro clínico, de acordo com o planejamento terapêutico estipulado.
4. **Pacientes com TVP recorrente:** repetir o exame ultrassonográfico no término do tratamento, ou um exame de acompanhamento após seis meses nos casos de anticoagulação prolongada, ou em qualquer momento na presença de sintomas.

## 6. Diagnóstico do Trombembolismo Pulmonar por Angiotomografia, Angio-RM e Angiografia Pulmonar

### 6.1. Angiotomografia de Tórax (Angio-TC)

O estudo Pioped II ^172^ avaliou o papel da angio-TC das artérias pulmonares associadas a angiotomografia venosa dos membros inferiores (angio-VTC) no diagnóstico do TEP. Os pacientes foram classificados clinicamente quanto à probabilidade do TEP pelo escore de Wells, ^33^ e os resultados pré-teste foram comparados aos resultados pós-teste. Os autores demonstraram que o exame de angio-TC, quando associado ao angio-VTC, possui VPP de 96% em pacientes com alta probabilidade clínica para TEP, e VPN de 97% em pacientes de baixa probabilidade clínica. Em pacientes com probabilidade clínica intermediária, tanto o VPN quanto o VPP foram de 92%, demonstrando a excelente resolutividade do método.

A angio-TC também possibilita a avaliação da aorta, do parênquima pulmonar, da parede torácica e do espaço pleural, com excelente resolução espacial, e a realização de diagnósticos alternativos nos casos de suspeita do TEP, o que pode ocorrer em até 2/3 dos casos, como a dissecção de aorta, o pneumotórax, a pneumonia e o câncer de pulmão, permitindo que o tratamento para cada uma destas situações seja adequadamente instituído. ^173^

Atualmente a angio-TC é o exame de escolha para avaliação de pacientes com suspeita de TEP após estratificação de risco clínico, sendo um exame de angio-TC negativo para TEP em pacientes com baixa probabilidade clínica e um procedimento suficiente para afastar este diagnóstico. Um exame de angio-TC positivo para TEP em paciente com alta probabilidade clínica confirma o diagnóstico.

Visto que a angio-TC é o exame de escolha para o diagnóstico do TEP, trabalhos que tentam caracterizar fatores prognósticos através da avaliação tomográfica do VD vêm surgindo na literatura. ^174 - 177^

Schoepf et al., ^177^ em estudo retrospectivo, demonstraram que o aumento do VD, avaliado pela angio-TC através da relação entre as dimensões do VD e do VE (anormal quando > 0,9), é um fator independente de mortalidade (OR = 5,17; IC 95%), ^1 , 16 , 35 , 63^ o que confirma o potencial uso dessa ferramenta na estratificação de risco dos pacientes com embolia pulmonar aguda.

**Figura 23:**
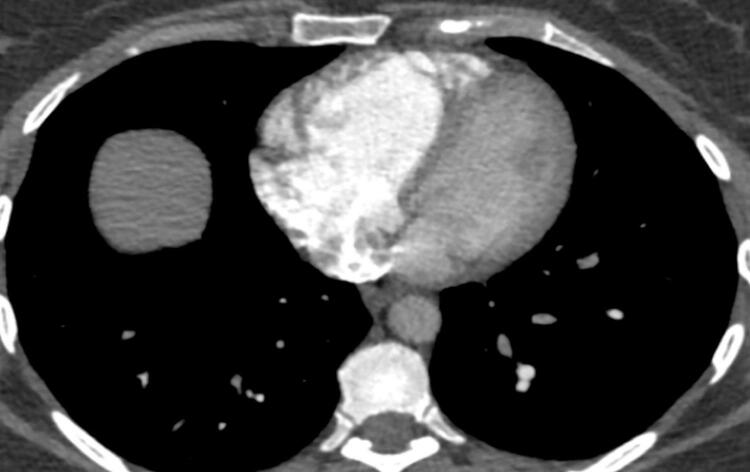
– Angio-TC positiva para TEP agudo. Sinais de disfunção do ventrículo direito; notar aumento das dimensões do VD (relação VD/VE >1) e abaulamento à esquerda do septo interventricular.

Araoz et al. ^175^ avaliaram três achados na angio-TC (abaulamento do septo interventricular, carga embólica e relação VD/VE) para avaliar o risco de óbito precoce, definido como morte durante a internação ou 30 dias após a angio-TC para diagnóstico do TEP ( Figura 23 ). Concluíram que, como preditor de óbito precoce, o abaulamento do septo interventricular poderia apresentar algum valor, com alta especificidade (87% a 88%) e baixa sensibilidade (18% a 21%). Os outros dois fatores avaliados não aumentaram o risco de morte precoce na amostra estudada.

#### 6.1.1. Técnica e Protocolos de Exames

Ao realizar uma angio-TC de artérias pulmonares, o objetivo é alcançar o máximo de opacificação arterial pulmonar, mantendo o mínimo possível de contaminação venosa e artefatos de movimentos, além de reduzir as doses de radiação e contraste.

Os principais parâmetros a serem controlados são:

**a) Protocolos de aquisição das imagens:** dependem do equipamento disponível e de características como tamanho e peso do paciente. Idealmente, deve-se:

- Realizar aquisição em apneia inspiratória, treinando o paciente para evitar manobra de Valsalva ou pausa respiratória;
- Utilizar o *time bolus* ou *bolus tracking* com o ROI no tronco da artéria pulmonar para monitorizar a chegada do meio de contraste na circulação arterial pulmonar e realizar o exame no tempo adequado;
- Adquirir as imagens em direção das bases para os ápices pulmonares, minimizando artefatos de movimentos nas regiões mais importantes, bem como reduzir o artefato decorrente de contraste concentrado na veia cava superior;
- Adquirir e reconstruir as imagens com espessura fina (1mm ou menos) e sem intervalos entre as imagens para permitir reconstruções multiplanares sem perda de resolução espacial;
- O estudo deve abranger todas as estruturas torácicas, o que permite avaliar eventuais diagnósticos diferenciais para a queixa do paciente;
- Baixar Kv, o que permite atenuar de modo significativo o iodo e reduzir substancialmente a dose de radiação. Reduções do Kv provocam aumento no ruído da imagem: a utilização de 100 Kv, ao invés dos tradicionais 120 Kv, deve ser a de preferência, desde que a relação sinal/ruído seja mantida em níveis aceitáveis. Pacientes obesos podem precisar de Kv mais altos;
- Utilizar a reconstrução iterativa, o que permite reduzir as doses de radiação e manter o ruído da imagem aceitável.

**b) Protocolo de injeção do meio de contraste:** dependem do tipo de contraste e do acesso venoso disponíveis. Idealmente, devem ter:

- Acesso venoso antecubital calibroso que permita a injeção do meio de contraste por meio de bomba injetora a uma velocidade mínima de 4 mL/s;
- Utilização de meios de contraste mais concentrados, como 350 mgI/mL.

## 6.2. Tomografia Computadorizada de Dupla Energia (TCDE)

**Figura 24:**
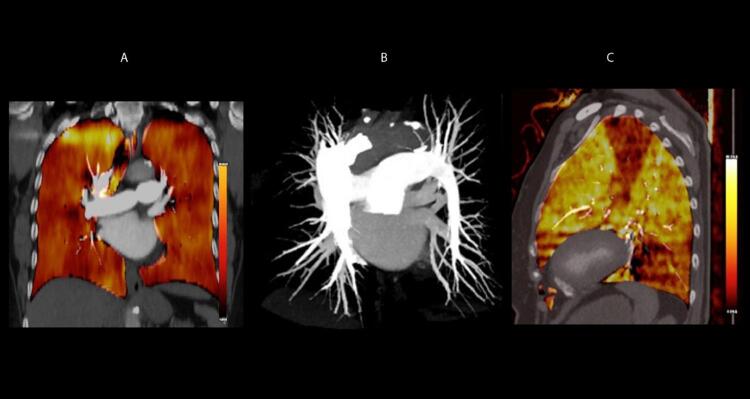
– Angiotomografia computarizada de artérias pulmonares em dupla energia. A: mapa de iodo refletindo a distribuição homogênea do meio de contraste no parênquima pulmonar; B: reconstrução angiográfica MIP. A TCDE consegue aliar a informação funcional com a imagem anatômica angiográfica. C: mapa de iodo de TCDE em outro paciente, demonstrando defeitos de perfusão em cunha nos lobos superior e inferior esquerdos, compatíveis com infartos pulmonares. As imagens angiográficas (não demonstradas) confirmaram o diagnóstico de TEP agudo.

A TCDE é um método capaz de adquirir imagens simultâneas (ou quase) sob duas diferentes energias. ^178^ Na avaliação de TEP, as principais vantagens desse método são ( Figura 24 ):

- Gerar como subproduto o mapa de iodo, que reflita naquele instante a distribuição do meio de contraste pelo parênquima pulmonar. A avaliação conjunta do mapa de iodo com as imagens angiográficas pode sensibilizar a detecção de pequenos trombos em ramos arteriais pulmonares distais; ^179 , 180^ permitindo reconstruções monoenergéticas.
- Por outro lado, reconstruções monoenergéticas a baixas energias (p. ex., 50Kev) aumentam a atenuação do iodo, ainda que tornem a imagem mais ruidosa. Este tipo de reconstrução pode melhorar as imagens angiográficas nos casos de opacificação vascular subótima ou permitir reduções na dose de contraste, p. ex., em pacientes com disfunção renal. ^179 , 180^

## 6.3. Gerações Antigas de Tomógrafos

Realizar uma angio-TC de artérias pulmonares em equipamentos mais antigos pode ser um grande desafio. Entretanto, ajustando-se os parâmetros de aquisição e otimizando o uso do contraste, pode ser necessária a utilização de maiores volumes ou adequar a velocidade de infusão para compensar uma aquisição mais lenta para que seja possível obter imagens com qualidade diagnóstica.

## 6.4. Critérios Diagnósticos

O diagnóstico de TEP agudo pela angio-TC baseia-se na identificação de trombos oclusivos ou não no interior dos ramos arteriais pulmonares. Os critérios diagnósticos de TEP agudo na angio-TC são: ^181^

- Falha de enchimento oclusiva, determinando aumento no calibre do vaso acometido ( Figura 25A );
  **Figura 25:**
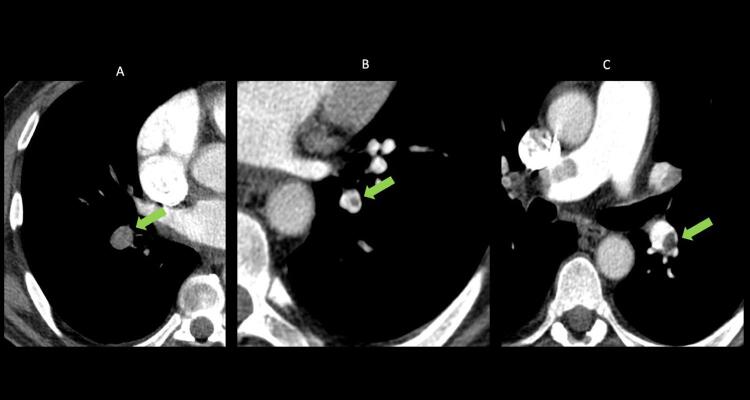
– A: falha de enchimento oclusiva determinando aumento do calibre do vaso; B: falha de enchimento, central, margeada por meio de contraste; C: falha de enchimento, aderida à parede do vaso e formando um ângulo agudo com ela.
- Falha de enchimento não oclusiva, central, margeada por meio de contraste ( Figura 25B );
- Falha de enchimento não oclusiva, aderida à parede do vaso e formando um ângulo agudo com ela ( Figura 25C ).

Trombembolismo pulmonar agudo é a principal causa para falhas de enchimentos em ramos arteriais pulmonares. São eventuais *pitfalls* e diagnósticos diferenciais:

- Artefatos de fluxo e/ou de movimentos;
- Interpretação incorreta da anatomia;
- Vasoconstricção reflexa;
- TEP crônico;
- Êmbolos não trombóticos;
- Alterações congênitas (agenesia e/ou hipoplasia de artérias pulmonares);
- Doenças inflamatórias (vasculites, mediastinite fibrosante);
- Neoplasias (trombos e/ou invasão tumoral ou neoplasias primárias das artérias pulmonares).

**Figura 26:**
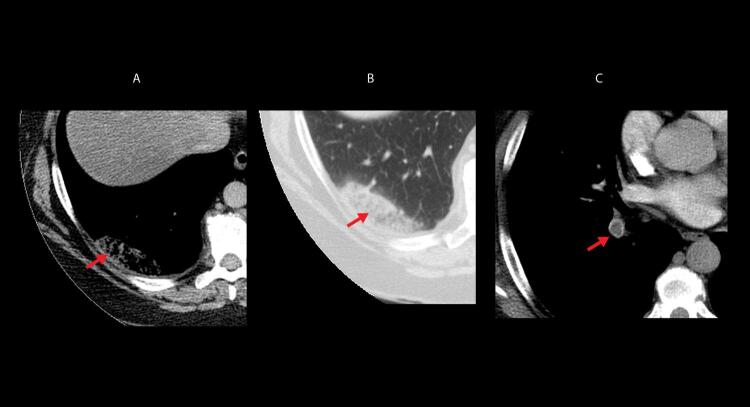
– Infarto pulmonar. A e B: opacidade periférica, com focos hipertransparentes de permeio e sem aerobroncogramas. C: trombo agudo em ramo arterial pulmonar segmentar basal para o lobo inferior direito.

O sinal indireto de TEP agudo na tomografia computadorizada é o infarto pulmonar ( Figura 26 ), que pode ser identificado mesmo nos estudos sem contraste. Nessa circunstância, faz-se necessária a complementação do estudo com técnica angiográfica para confirmar o diagnóstico. A imagem típica do infarto pulmonar é uma opacidade periférica, de base pleural, com focos hipertransparentes de permeio mais bem caracterizadas na janela de mediastino (sinal do miolo de pão) e sem aerobroncogramas. ^12 , 182^

## 6.5. Critérios Prognósticos

**Figura 27:**
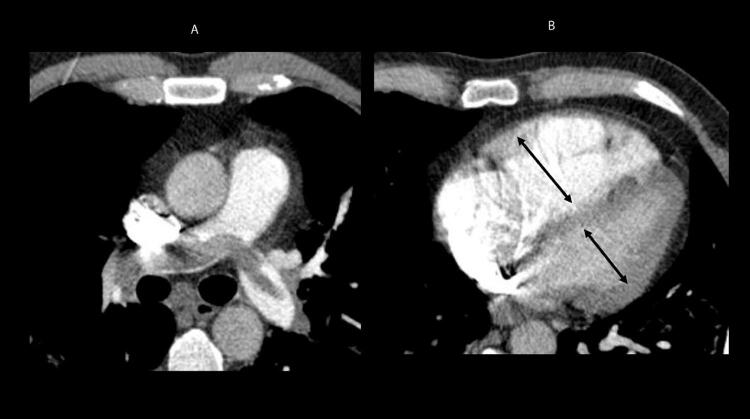
– Angio TC de artérias pulmonares. A: sinais típicos de TEP agudo; B: sinais de sobrecarga das cavidades cardíacas direitas. Notar relação VD/VE >1,0 e o aspecto retificado do septo interventricular.

As alterações que podem ser caracterizadas na angio-TC com valor prognóstico estão relacionadas à sobrecarga aguda das cavidades cardíacas direitas. Ainda que as dilatações das veias cava superior e ázigos e do tronco da artéria pulmonar, bem como as medidas objetivas da função ventricular direita, tenham sido citadas como marcadores prognósticos, o sinal mais importante de disfunção ventricular direita é o aumento das dimensões do ventrículo direito com a relação VD/VE acima de 1,0 no plano axial da tomografia ( Figura 27 ). ^181^

## 6.6. Contraindicação e Situações Especiais

Considerando a importância da angio-TC no diagnóstico do TEP agudo, a única contraindicação absoluta para a realização desse exame é o antecedente de alergia grave ao meio de contraste iodado. Nesses casos, as alternativas diagnósticas podem ser a cintilografia V/Q, a angio-RM, a angio-TC com gadolíneo ou a USG *Doppler* de MMII seriada. Antecedentes de reações alérgicas leves e moderadas podem ser contornadas com o uso de pré-medicações para a dessensibilização.

Existem outras situações especiais nas quais pode ser conveniente adequar o protocolo ou substituir a angio-TC por outros métodos diagnósticos:

### Cenário 1: mulher jovem em idade reprodutiva ou gestante

Nesses casos, para evitar a exposição do tecido mamário à radiação ionizante e, especialmente, nas pacientes com radiografia de tórax normal, as alternativas poderiam ser a cintilografia V/Q (ou apenas a fase de perfusão) ou a USG *Doppler* de MMII seriada.

Quando a opção for pela angio-TC, o protocolo deve ser otimizado, garantindo um acesso venoso e velocidades de injeção adequados para conseguir um exame de alta qualidade diagnóstica e evitar repetições.

### Cenário 2: insuficiência renal crônica em tratamento conservador

Não é uma contraindicação absoluta. Preferencialmente, deve-se antes hidratar o paciente, mas com cautela para evitar a congestão. Nos casos de urgência, a angio-TC pode ser realizada mesmo sem preparo prévio. A cintilografia V/Q e a USG *Doppler* de MMII seriada permanecem como alternativas.

Caso esteja disponível, utilizando tomógrafos de dupla energia e reconstruções monoenergéticas é possível reduzir a dose do meio de contraste iodado e ainda assim garantir a opacificação vascular adequada.

### Cenário 3: Paciente internado em UTI sem condições de transporte

Nesses casos, optar por exames que podem ser executados à beira do leito, como a ecocardiografia e a USG *Doppler* de MMII.”

## 6.7. Angiorressonância Magnética (Angio-RM)

A angio-RM é uma alternativa à angio-TC, que permite a realização de estudo angiográfico direto das artérias pulmonares. ^174 , 183^ Esse método diagnóstico foi avaliado pelo estudo Pioped III, ^183^ que associou a angio-RM de artérias pulmonares à angiorressonância magnética venosa (angio-RMV) dos membros inferiores para o diagnóstico.

A angio-RM apresenta algumas vantagens em relação à angio-TC. As principais são a ausência de radiação e o uso de um meio de contraste (gadolínio) que pode ser utilizado em pacientes com alergia a contraste iodado. ^183^ Além disso, o exame de angio-RM possibilita a realização de outras técnicas de avaliação, como a perfusão pulmonar, a quantificação de fluxo nos grandes vasos e a avaliação da função cardíaca. ^174 , 183^

Atualmente, pacientes com insuficiência renal, sobretudo dialítica, e que até pouco tempo eram um nicho para a ressonância magnética, têm esta indicação revista pela ocorrência de fibrose sistêmica progressiva relacionada ao uso do gadolínio nesses pacientes. ^174^

As principais desvantagens do método são menor resolução espacial, maior custo, maior complexidade, maior tempo de exame, menor disponibilidade e dificuldade de monitorar pacientes graves no interior do equipamento devido ao alto campo magnético. ^174 , 183^

Os resultados do estudo Pioped III(183) mostraram que até 25% das angio-RM foram consideradas tecnicamente inadequadas. Ao incluir esses exames tecnicamente inadequados, o método conseguiu diagnosticar o TEP em 57% dos pacientes. Quando apenas os exames considerados tecnicamente adequados foram analisados separadamente, a angio-RM teve sensibilidade de 78% e especificidade de 99% no diagnóstico do TEP. Quando a angio-RM foi associada à angio-RMV, o método mostrou sensibilidade de 92%, superior a angio-RM isolada, e especificidade de 96% no diagnóstico. Contudo, em até 52% dos pacientes, os exames foram considerados inadequados, o que acarretou um grande problema.

O estudo Pioped III ^183^ concluiu que a utilização da angio-RM para o diagnóstico do TEP deve ser realizada apenas em centros de referência em ressonância magnética e quando outros métodos de avaliação estiverem contraindicados. Atualmente, a principal indicação atual da ressonância magnética é como método alternativo à TC em pacientes com alergia ao contraste iodado. ^174 , 183^

## 6.8. Angiografia Digital com Subtração

Por muito tempo, a angiografia digital foi considerada como método padrão-ouro para o diagnóstico de TEP. O caráter invasivo, as maiores doses de exposição à radiação ionizante e complicações do procedimento com relatos de até 0,5% de mortalidade, 1% de complicações graves e 5% de complicações menores, ^184^ associadas ao avanço tecnológico da angio-TC e que possibilitam uma acurácia semelhante de forma menos invasiva, são fatores determinantes para que a angiografia digital tenha praticamente caído em desuso para o diagnóstico de TEP agudo. Entretanto, o método permanece como uma importante ferramenta diagnóstica na avaliação do TEP crônico, especialmente nos pacientes candidatos ao tratamento por angioplastia com balão.

## 6.9. Recomendações

- A indicação dos métodos de imagem para o diagnóstico de TEP agudo deve ser baseada na condição hemodinâmica do paciente e na avaliação clínica da probabilidade pré-teste, com a aplicação de regras validadas (Wells, Geneva, Perc) associadas ao teste do D-dímero. Tem como objetivo evitar o uso desnecessário dos métodos de imagem (classe I/nível de evidência A);
- A angio-TC é o método de imagem de escolha para o diagnóstico do TEP agudo. Pode ser substituída pela cintilografia V/Q nos casos de alergia grave ao iodo ou em situações especiais como gestantes ou pacientes com insuficiência renal grave. A angio-RM fica reservada aos centros com experiência no método ou nos casos em que a angio-TC e a cintilografia V/Q sejam inapropriadas ou estejam indisponíveis;
- O diagnóstico de TEP agudo deve ser rejeitado nos pacientes com probabilidades baixa ou intermediária e angio-TC negativa (classe I/nível de evidência A);
- O diagnóstico de TEP agudo deve ser aceito nos pacientes com probabilidade intermediária ou alta e angio-TC, demonstrando trombos em ramos segmentares ou proximais (classe I/nível de evidência B);
- Não é recomendada a angio-VTC dos membros inferiores em virtude da alta exposição à radiação ionizante, devendo ser substituída pela USV para a avaliação de TVP (classe III/nível de evidência B);
- Nas pacientes gestantes, tanto a angio-TC quanto a cintilografia V/Q podem ser usadas. Com as técnicas modernas, ambos os métodos causam pouca exposição materna e fetal à radiação ionizante. ^78 , 185^ Nos casos com RX normal, tanto a angio-TC quanto a cintilografia V/Q podem ser utilizadas; por outro lado, nos casos com RX alterada, a recomendação é prosseguir com angio-TC (classe IIa/nível de evidência C);
- O aumento nas dimensões das cavidades cardíacas direitas, com a relação VD/VE > 1,0, está ligado a mortalidade ligada ao TEP agudo 5 vezes maior, ^186^ devendo esta ser considerada como um critério tomográfico de disfunção ventricular direita e marcador de pior prognóstico, mesmo em pacientes clinicamente considerados de baixo risco (classe IIa/nível de evidência B).

Os fluxogramas diagnósticos em pacientes hemodinamicamente estáveis e instáveis, de acordo com a probabilidade clínica de TEP, estão nos Quadros 5 e 6 . No Quadro 12 , estão apontadas as vantagens e desvantagens do uso da angiotomografia pulmonar no diagnóstico do TEP.

**Quadro 12:** – Vantagens e desvantagens do uso da angiotomografia no diagnóstico do TEP

ANGIOTOMOGRAFIA PULMONAR	
VANTAGENS	DESVANTAGENS
Grande disponibilidade	Exposição à radiação ionizante
Excelente acurácia	Necessidade do uso do meio de contraste iodado
Baixo número de estudos inconclusivos	Tendência de uso indiscriminado pela facilidade de acesso
Rápida execução	Sobrediagnóstico – capaz de identificar trombos subsegmentares, cujo significado clínico é incerto
Permite investigar diagnósticos alternativos	

## 7. Cintilografia Pulmonar

### 7.1. Evidências

#### 7.1.1. Introdução

Para efeitos práticos, define-se nesta parte o termo “cintilografia pulmonar” como um exame único contendo diferentes combinações entre cintilografia de perfusão pulmonar e cintilografia de inalação/ventilação pulmonar; ou mesmo o estudo de perfusão isolado. De acordo com as diretrizes internacionais disponíveis da Society of Nuclear Medicine and Molecular Imaging (SNMMI, Estados Unidos), da European Association of Nuclear Medicine (EANM, Europa) e da European Society of Cardiology (ESC, Europa), as principais indicações para o uso da cintilografia pulmonar serão listadas e discutidas a seguir. ^22 , 187 , 190^

**Indicação principal:** determinar a probabilidade de trombembolismo pulmonar (TEP) ^22 , 187 , 196^

**Indicações secundárias:**

- Registrar o grau de resolução de trombembolismo pulmonar no acompanhamento de TEP crônica;
- Quantificar a função pulmonar diferencial antes da cirurgia para ressecção de neoplasias pulmonares; ^197 - 199^
- Avaliar transplantes pulmonares; ^200 , 201^
- Avaliar doença cardíaca ou pulmonar congênita, ou doenças como *shunts* cardíacos, estenose de artéria pulmonar e fístulas arteriovenosas, bem como seu tratamento. ^202^
- Confirmar a presença de fístula broncopleural. ^203 - 205^
- Avaliar distúrbios pulmonares crônicos parenquimatosos como a fibrose cística; ^206 , 207^
- Avaliar TEP como causa de hipertensão pulmonar. ^208^

Considera-se como positivo para TEP: ^189^

- Discordância entre os estudos de ventilação ou inalação, e o estudo de perfusão em pelo menos um segmento ou dois subsegmentos, com hipocaptação presente apenas ou mais extensa na perfusão em contraste com inalação normal ( *mismatch* ), respeitando a estrutura pulmonar vascular (hipoperfusão com padrão em cunha e projetada a partir do centro até a periferia pulmonar).

São considerados negativos para TEP:

- Perfusão normal, mantendo as bordas normais dos pulmões;
- Padrões concordantes entre os estudos de inalação e perfusão de qualquer tamanho, forma ou número;
- Padrões discordantes entre inalação e perfusão sem padrão lobar, segmentar ou subsegmentar.

São considerados não diagnósticos para TEP:

- Anormalidades de inalação/perfusão generalizadas, não típicas de uma doença específica.

#### 7.1.2 Evidências Relacionadas aos Critérios de Interpretação dos Exames

Na avaliação da cintilografia pulmonar para a detecção de TEP, existem evidências científicas suficientes tanto para o uso de critérios bem definidos de interpretação, para os critérios modificados do estudo *Prospective Investigation of Pulmonary Embolism Diagnosis* (Pioped), Pioped II e Pisaped, quanto para interpretação baseada na experiência clínica do médico nuclear/imaginologista (método Gestalt).

Os critérios Pioped modificados mostraram-se mais acurados do que os originais Pioped, ^209 , 210^ e os chamados Pioped II (que usa menos categorias de análise) e Pisaped resultaram em um número menor de estudos “indeterminados” ou “incaracterísticos”. ^211 , 212^ Tanto o Pioped II quanto o Pisaped resultaram em performances equivalentes quando utilizaram radiografias torácicas em relação à interpretação em conjunto com cintilografia de inalação/ventilação pulmonar. ^213^ Não existem, contudo, estudos suficientes replicando esses resultados, inclusive comparações multicêntricas duplo-cegas entre as duas estratégias, o que não suporta a afirmação de que ambos os métodos tem acurácia equivalente.

Recomenda-se, assim, a associação de estudos de inalação/ventilação sempre que possível, principalmente pela elevação da especificidade do estudo combinado em comparação ao estudo comparativo com a radiografia. Em idosos, até 60% de falso-positivos podem resultar da ausência de inalação, pela elevada prevalência de doenças de vias aéreas. ^214^

A análise baseada na experiência do médico nuclear (Gestalt) também pode resultar em interpretação mais acurada do que nos estudos ao usar isoladamente critérios bem definidos. Para que essa interpretação seja a melhor possível, identificou-se que a opinião do médico deve ser informada levando-se em consideração o conhecimento detalhado dos diferentes critérios objetivos relacionados anteriormente. ^209^

No que diz respeito à aquisição de imagens cintilográficas tomográficas (tomografia por emissão de fóton único – Spect), existe considerável literatura suportando o seu uso, inclusive relacionada a estudos acoplados à tomografia computadorizada de baixa dose (Spect/CT de baixa dose), que parecem elevar a acurácia da interpretação. ^215^ O consenso mais recente da EANM (2019) sugere que as aquisições tomográficas (SPECT ou SPECT/CT) sejam sempre feitas por rotina, deixando as aquisições planas para quando não seja mais possível a aquisição tomográfica. ^188^

Ainda que não existam estudos multicêntricos robustos de comparação com imagens de cintilografia plana ou mesmo com a angio-TC, ^216^ ou estudos robustos prospectivos que acompanhem o desfecho dos pacientes, a exemplo da cintilografia plana e da angio-TC, ^190^ nos últimos foi relatada notável confirmação da elevação da acurácia diagnóstica da Spect e da Spect/CT. Existem ainda algumas incertezas sobre a interpretação de Spect e Spect/CT nos moldes do Pioped e derivados, o melhor protocolo Spect para as imagens de inalação/ventilação e qual o melhor protocolo de investigação ideal como um todo (Spect de perfusão e inalação, Spect/CT com ou sem contraste etc). ^213 , 216^ No entanto, sugere-se que sejam valorizados como positivos os achados a partir de um segmento completo de discordância entre a perfusão e a inalação ( *mismatch* ). Achados de um único *mismatch* subsegmentar não devem ser valorizados como suspeitos para TEP. ^166^

Frente aos dados apresentados, convém ressaltar que, independentemente do critério adotado para interpretação, é bem definido que há um prognóstico favorável para os pacientes com baixa probabilidade de TEP nos exames de cintilografia pulmonar, ^191 - 193^ e que um estudo normal de perfusão virtualmente exclui TEP. ^194 - 196^

#### 7.1.3. Acurácia Diagnóstica

Com relação à acurácia dos métodos, deve-se ressaltar a diferença entre os estudos mais antigos, feitos nas décadas de 1980 e 1990, e os mais recentes, realizados com máquinas atualizadas e métodos tomográficos. ^217^ Estudos mais antigos apresentavam acurácia significativamente menor, além de uma taxa de estudos não diagnósticos que se aproximaram de 65% no estudo Pioped original. Atualmente, essa taxa varia de 1% a 4%, porcentagem significativamente menor que a de outros métodos de investigação. ^189 , 209 , 217^

De modo geral, os valores de acurácia diagnóstica dos estudos mais recentes, sobretudo da Spect, igualam-se ou sobrepujam os da angiotomografia computadorizada de tórax (angio-TC), o que por vezes demonstra maior capacidade da cintilografia em detectar TEP subsegmentar no estudo Pioped II. Assim, não existem dados científicos robustos que sustentem a superioridade da angio-TC sobre a cintilografia. ^218^

Dessa forma, a cintilografia na forma de Spect é o exame com menor taxa de resultados inconclusivos (<3%). ^189 , 190^ Contudo, na prática, a angio-TC permanece como o método de escolha, principalmente pela rapidez de sua realização e sua maior disponibilidade, inclusive de 24 horas em regime de plantão, frente aos exames de medicina nuclear. ^176^

As principais diferenças de acurácia entre os estudos planos e Spect se dão pela maior detecção de alterações subsegmentares e nos segmentos mediais próximos ao mediastino, que no segundo caso podem detectar até 53% mais áreas sugestivas de TEP do que os estudos planos. ^219^ Os primeiros valores de sensibilidade (S) e especificidade (E) demonstraram importante variabilidade nos primeiros estudos: S = 67% para imagens planas e S = 93% para Spect em modelo animal (porcos); ^220^ S= 80% e E = 78% para imagens planas; S = 80% e E = 96% para Spect; ^221^ e S = 76% e E = 85% para imagens planas *versus* S = 97% e E = 91% para Spect. ^222^ Portanto, nos estudos iniciais, os valores de acurácia geralmente se elevaram da casa de 70-80% com imagens planas para acima de 90% quando foram usadas imagens Spect.

**Figura 28:**
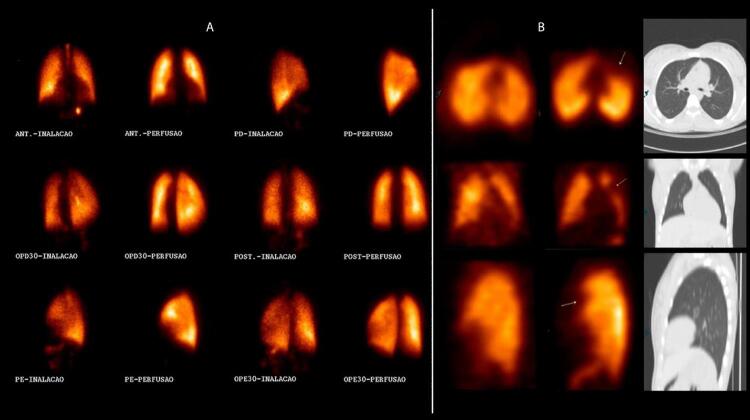
– A: série de imagens exemplificando uma cintilografia pulmonar V/Q Planar, com uso de DTPA/MAA marcados com 99mTc, nas projeções anterior, posterior, laterais e oblíquas, mostrando inalação e perfusão normais. B: série de imagens de cintilografia pulmonar V/Q Spect-CT, com uso de DTPA/MAA marcados com 99mTc da esquerda para direita: primeira coluna, ventilação Spect; segunda coluna, perfusão Spect; e terceira coluna, TC tórax. A imagem de ventilação é normal, já a perfusão evidencia déficit perfusional no segmento inferior do lobo superior esquerdo (seta) e TC normal, configurando TEP.

Estudos mais recentes em mais de 5.000 casos com uso de Spect demonstraram valor preditivo negativo entre 97-99%, S = 96-99% e E = 96-98%, o que dá largo suporte para o seu uso em detrimento das imagens planas. ^222 - 229^ A incorporação do Spect com tomografia computadorizada (Spect/CT) torna o método ainda superior, ao elevar sua especificidade e adicionar a capacidade da tomografia de baixa dose, o que ajuda no diagnóstico diferencial ao identificar enfisema, pneumonia e/ou outras alterações parenquimatosas que causem compressão vascular, e, portanto, possam explicar os defeitos perfusionais, ^215 , 230^ ( Figura 28 )

Nos artigos mais recentes, a comparação com a angio-TC trouxe novas respostas, mas ainda há controvérsias sobre a superioridade de performance de um método sobre o outro. ^231^ Aparentemente, Spect e Spect/CT são superiores e demonstram diagnóstico de TEP com maior acurácia em casos de doenças pulmonares, como comorbidades que podem dificultar o diagnóstico pela angio-TC. ^223 , 232 - 234^ Em uma revisão sistemática e metanálise, Spect foi superior à angio-TC, ^231^ e estudos de comparação direta demonstram que a Spect é superior à angio-TC em análise de curva ROC. ^235^ Também é importante ressaltar quer a Spect possui sensibilidade superior em pacientes com TEP crônica, onde é considerado o exame de referência. ^236^

Conclui-se que ambos os exames apresentam vantagens e desvantagens, sendo a cintilografia superior à angio-TC em casos com outras doenças concomitantes, TEP crônica e quando a angio-TC resulta em exames indeterminados. ^223 , 231 - 234^ Assim, quando ambas as modalidades estão disponíveis, a cintilografia pulmonar com aquisição Spect (ou Spect/CT, preferencialmente) deve ser recomendada, por não possuir contraindicações, apresentar menor taxa de exames indeterminados e pela menor exposição da mama feminina à radiação (ver abaixo). ^237 - 241^

No que diz respeito ao estudo isolado de perfusão pulmonar pela cintilografia, comparações com a angiografia pulmonar como método padrão-ouro demonstraram sensibilidade de 86% e especificidade de 93% para perfusão. Quando os critérios Pisaped foram aplicados na população do estudo Pioped-II, a sensibilidade ponderada do estudo de perfusão foi de 82%, e a especificidade ponderada foi de 96%. ^213^ Estudos de perfusão isolada são recomendados em indivíduos com suspeita de TEP maciça e na gravidez, como comentado abaixo. Utilizar a tomografia computadorizada de baixa dose em substituição a um estudo inalatório eleva a sensibilidade em relação à perfusão isolada ;porém, essa estratégia leva a uma taxa maior de falso-positivos ^215 , 23 , 242^ e eleva a exposição à radiação em gestantes.

#### 7.1.4. Indicações da Cintilografia Pulmonar por Limitações da Angiotomografia Pulmonar

Além do que foi discutido aqui, a cintilografia pulmonar tem sido considerada a escolha preferencial sobre outros métodos em casos de gravidez, considerando-se a menor taxa de exposição à radiação para a mama de mulheres grávidas, além de necessidade de uso em algumas situações de limitação do uso de contraste pela angio-TC. Tais indicações serão detalhadas a seguir.

**a) Exposição à radiação e uso na gravidez:**

Uma vez que estudos de inalação/ventilação e radiografias de tórax são comumente normais em indivíduos jovens, é comum recomendar a omissão dessa parte do estudo em indivíduos sabidamente em risco para efeitos da radiação, como grávidas no primeiro trimestre de gravidez, por conta da provável maior vulnerabilidade do feto aos efeitos da radiação. ^189 , 190^

Na gravidez, de início recomenda-se um protocolo apenas com estudo perfusional e atividade reduzida do radiofármaco (ver seção sobre técnicas de realização do exame). Uma vez normal, essa fase do estudo exclui TEP, deixado à realização da fase de inalação apenas em casos de exceção e dúvida de positividade no dia seguinte ao exame de perfusão. ^243^ Dessa forma, garante-se menos exposição à radiação do que uma angiotomografia pulmonar e ausência de exposição ao contraste iodado. ^189^

O órgão materno critico a ser protegido, neste caso, é a mama, que absorve doses de radiação entre 8,6 a 44 mSv pela angio-TC ^237 , 238 , 244^ e menos de 1 mSv na cintilografia de perfusão pulmonar isolada, sem associação da tomografia ou estudo inalatório. ^237^ A doses para os fetos parecem ser baixas e similares em ambos os métodos.

**b) Outras limitações clínicas da angiotomografia:**

Por conta do uso de contraste iodado intravenoso, a angio-TC pode não ser realizada em uma grande parte dos pacientes graves, como aqueles com insuficiência renal, doentes em estado crítico, que sofreram infarto do miocárdio recente, como necessidade de suporte ventilatório e história de alergia/anafilaxia ao contraste iodado. Nesses casos (além daqueles citados no item A) e no tópico *Acurácia diagnóstica* , a cintilografia pulmonar deve sempre ser indicada, por não possuir contraindicações, pela ausência de complicações relacionadas ao método, pela baixa taxa de inconclusivos e em raros casos de incapacidade de realizar o exame. ^189 , 190 , 223^

#### 7.1.5. Protocolo

**1. Etapa de ventilação/inalação**

**a. Radiofármacos:**

**i. Gases inertes:**

**1.** ^133^ **Xe:**

- Historicamente, é o fármaco utilizado para as etapas de ventilação da cintilografia pulmonar. ^187 , 245 , 246^
- Possui meia-vida de 5 dias e energia (baixa) de 81keV. ^187 , 209^
- No estudo do Pioped I, a técnica de ventilação única é a mais usada. ^187 , 209^
- O ^133^ Xe é inalado durante os primeiros 20 segundos, e uma imagem deve ser obtida na projeção posterior. ^187 , 247^
- Não há disposição deste gás no Brasil.

**2.** ^81m^ **Kr:**

-  É produzido por um gerador de rubídio ( ^81^ Ru), de alto custo. ^187 , 248^
- Tem a energia gama ideal (193 keV) e meia-vida é de 13 segundos. ^187 , 249^ Quando um paciente está respirando ar com ^81m^ Kr em uma frequência respiratória normal, a concentração alveolar regional desse gás fica em um estado estacionário proporcional à ventilação regional. ^187^ Durante a ventilação de ^81m^ Kr no estado estacionário, é possível realizar várias imagens planares ou aquisição Spect. Muito recentemente, a combinação de Spect com TC de baixa dose foi descrita. ^187 , 215^
- O ^81m^ Kr é um gás verdadeiro que não causa artefatos por conta da deposição das vias aéreas centrais. Uma vantagem é que a ventilação e a perfusão podem ser visualizadas simultaneamente, uma vez que a energia gama do ^81m^ K é maior que a do ^99m^ Tc (usado como marcador de perfusão). ^187^
- A baixa exposição à radiação faz com que o ^81m^ Kr seja a melhor escolha para crianças.
- Não há disposição desse gás no Brasil.

**ii. Aerossóis radio marcados:**

1. Dada a indisponibilidade dos gases inertes ^133^ Xe e ^81m^ Kr em território nacional para a etapa de ventilação, geralmente são usados radioaerossóis.
2. Um aerossol é um sistema bifásico relativamente estável no tempo, composto por partículas suspensas no ar. As partículas radiomarcadas podem ser líquidas, sólidas ou uma combinação das duas. A porcentagem de partículas remanescentes no pulmão após a inalação (fração de deposição) depende das propriedades aerodinâmicas das partículas, principalmente de seu tamanho. A fração de deposição é de até 50%, com nanopartículas ultrafinas (diâmetro de 0,02mm), que são depositadas predominantemente na região alveolar por difusão. ^187 , 250^
3. O aerossol radiomarcado mais comumente utilizado é o ^99^ mTc-DTPA, que é eliminado da região alveolar por difusão transepitelial, apresentando meia-vida biológica de 80 minutos em indivíduos saudáveis não tabagistas; 45 minutos em fumantes passivos saudáveis es 24 minutos em tabagistas ativos saudáveis. ^251^
4. Outra opção de aerossol radiomarcado disponível é o technegas, que consiste em partículas extremamente pequenas de grafite sólido, radiomarcadas com ^99^ mTc, geradas a altas temperaturas e ^252 , 253^ que apresentam diâmetro de cerca de 0,005 a 0,2 micrômetros, ^254^ são hidrofóbicase apresentam tendência a se agruparem, razão pela qual devem ser utilizadas até 10 minutos após serem geradas. Ficam suspensas em gás argônio (tendo, portanto, propriedade de gás). Estudos demonstram que a utilização de ^99^ mTc-technegas com ^81m^ Kr geram resultados comparáveis. ^255 - 259^ Ressalta-se, entretanto, a baixa disponibilidade de equipamentos de technegas no Brasil, especialmente em função do alto custo.

**b. Protocolos de imagens:**

i. Como a embolia pulmonar é uma condição ameaçadora da vida, a recomendação é que o diagnóstico seja realizado o mais breve possível, usualmente em protocolos de 1 dia (ventilação e perfusão). Estudos de ventilação pulmonar, que servirão de base para comparação com o devido estudo perfusional, devem ser obtidos antes da aquisição das imagens de perfusão, com a necessidade de atividade radioativa muito baixa. A aquisição sequencial de imagens perfusionais de baixa a moderada atividade radioativa permite imagens comparativas de ótima qualidade, com diagnóstico em pouco tempo.

ii. Sempre que possível, deve-se proceder com aquisições tomográficas, como segue:

1. Ventilação com o devido aerossol radiomarcado, em posição supina, usando dose de 25% em relação ao que será utilizado para o estudo perfusional (usualmente 25 a 30 MBq para ventilação).
2. Em uma *gamma camera* com grande campo de visão, preferencialmente com 2 ou 3 cabeças, usar colimador de propósito geral. Serão obtidas, ao menos, 128 imagens, em matriz de aquisição de 64 x 64 e tempo por imagem de 10s.
3. As imagens de perfusão devem ser obtidas imediatamente após as imagens de ventilação. Para evitar movimentações do paciente, proceder com o acesso venoso periférico utilizado em imagens de perfusão antes do início da fase de ventilação.
4. **Etapa de Perfusão**

**a. Radiofármaco:**

i. O radiofármaco utilizado é o macroagragado de albumina humana marcado com ^99^ mTc ( ^99^ mTc-MAA).

**b. Protocolos de imagens:**

- O paciente deve estar em posição supina, com o acesso venoso previamente instalado (previamente à aquisição de imagens de ventilação – objetivando a menor movimentação possível entre as etapas);
- Administrar, através do acesso venoso, atividade entre 100 e 120 MBq de ^99^ mTc-MAA (número de partículas de MAA de aproximadamente 200.000 a 700.00 em situações clínicas específicas, como hipertensão pulmonar e pesquisa de *shunt* direita/esquerda. O número de partículas deve ser reduzido para um valor entre 100.000 e 150.000).
- Na mesma *gamma camera* em que foram adquiridas as imagens de ventilação, sem qualquer movimentação do paciente, as imagens devem ser adquiridas, como seguem:
- 128 imagens tomográficas (Spect) em matriz 64 x 64, 5 segundos por imagem. ^260^

**3. Apenas em etapa de perfusão:** em pacientes gestantes e casos de suspeita de TE maciço, é indicada a realização da etapa de perfusão, apenas.

#### 7.1.6. Reconstrução de imagens

As imagens devem ser reconstruídas de forma iterativa, utilizando-se OSEM ( *ordered-subset expectation maximization* ), e mediante protocolo sugerido com 8 *subsets* e 2 iterações. ^219 , 260 , 261^

#### 7.1.7. Interpretação

**a)** Nas cintilografias V/Q Spect e V/Q planar, as imagens devem ser interpretadas com base em:

- **i.** Critérios básicos para avaliação de estudos de imagem;
- **ii.** Conhecimento e experiência do médico interpretador, utilizando o princípio “Gestalt”;
- **iii.** Probabilidade pré-teste, de acordo com princípios de interpretação holísticos.

**b)** Para que sejam clinicamente relevantes, é importante que as cintilografias pulmonares sejam descritas como positivas ou negativas em relação à presença de TEP (EP: SIM ou NÃO), não devendo ser baseadas em categorias de probabilidades. ^84^

**c) Critérios básicos para interpretação de cintilografias pulmonares V/Q:**

**i. Ausência de EP é relatada quando houver:**

- **i.1** . Perfusão pulmonar normal;
- **i.2.** Defeitos V/Q sobrepostos ou reversíveis (presença de alteração na ventilação, sem sobreposição perfusional) de qualquer tamanho, forma ou número, e na ausência de *mismatch* verdadeiro;
- **i.3** . *Mismatch* sem padrão lobar, segmentar ou subsegmentar.

**ii. Presença de EP é relatada quando houver:**

- **ii.1** . *Mismatch* V/Q de pelo menos 1 segmento ou 1 subsegmento que siga a anatomia de perfusão pulmonar.
- **iii. O estudo é não diagnóstico quando:**
- **iii.1.** Múltiplas anormalidades V/Q são observadas, não típicas de doenças específicas.

#### 7.1.8. Trombembolismo Pulmonar Crônico

TEP crônico é uma entidade médica diferente do TEP agudo, com surgimento insidioso e que sem tratamento, é progressivo e apresenta mau prognóstico. ^262 , 263^ A mortalidade decorre de hipertensão pulmonar, falência cardíaca direita e arritmias. De modo geral, cintilografias pulmonares V/Q são auxiliares no diagnóstico de hipertensão pulmonar trombembólica crônica, ^208 , 264^ com sensibilidade e especificidade superiores às de tomografias computadorizadas de múltiplos detectores. ^265^

#### 7.1.9. Achados Diagnósticos Adicionais

A realização de cintilografias pulmonares ventilatórias e perfusionais, com aquisição de imagens topográficas (V/Q Spect) têm sido capazes de demonstrar outras situações clínicas que não apenas a embolia pulmonar, como DPOC, insuficiência cardíaca esquerda e pneumonia. Em aproximadamente 39% dos pacientes que se submetem ao procedimento diagnóstico e não apresentam TEP, e em 22% com TEP, algum dos seguintes achados adicionais estará presente. ^223^

- **DPOC:** caracteriza-se por defeitos pareados ventilatórios e perfusionais. Frequentemente, os defeitos ventilatórios são mais pronunciados que os defeitos perfusionais, fenômeno conhecido como *mismatch* reverso. ^266 , 267^ A correlação significativa entre o grau de anormalidades ventilatórias identificadas ao estudo cintilográfico e os resultados de testes de função pulmonar foi descrita. ^268^ A TEP é frequente em pacientes com DPOC, ^269^ sendo responsável por cerca de 10% das mortes de pacientes com DPOC estável. ^270^
- **Insuficiência cardíaca esquerda:** a perfusão sofre redistribuição rumo às regiões superiores dos pulmões. ^271 , 272^ A redistribuição ventilatória é menos extensa que a redistribuição perfusional. O *mismatch* pode ser observado nas regiões dorsais e apresenta padrão difuso não segmental, não devendo ser interpretado como EP.
- **Pneumonia:** apresenta-se com defeitos ventilatórios e perfusionais pareados ( *matched)* , sendo que os primeiros usualmente são maiores, o que causa *mismatch* reverso. ^273 , 274^ Um achado sugestivo de pneumonia é o *stripe sign* , que se refere a manutenção de perfusão na superfície pleural periférica à uma área central de defeito pareado. ^275^ O padrão *stripe sign* é melhor identificado em estudos com aquisições tomográficas.

#### 7.1.10. *Pitfalls* na Interpretação de Estudos V/Q

Assim como em qualquer método diagnóstico, é fundamental que o médico nuclear responsável pelo laudo do referido estudo conheça os possíveis e inerentes erros ao método, como os que seguem:

- Artefatos técnicos podem surgir a partir da manipulação pré-injeção do ^99m^ Tc-MAA. A retirada de sangue de um paciente através de uma seringa que contenha a solução de ^99m^ Tc-MAA pode levar a agregação de partículas, criando os chamados *hotspots* . Defeito semelhante pode surgir decorrente da falha de ressuspensão das partículas de ^99m^ Tc-MAA previamente à administração endovenosa.
- Imagens planares podem subestimar a presença e/ou a extensão das anormalidades perfusionais, decorrentes da sobreposição de áreas normalmente perfundidas. Esse artefato é eliminado através das imagens cintilográficas em cortes tomográficos (Spect).
- Sempre que possível, o uso de technegas é preferível em relação aos aerossóis líquidos em pacientes com DPOC. Raramente, em pacientes com enfisema pulmonar, as partículas de technegas podem ficar presas em bolhas, levando a má interpretação de possível *mismatch.* ^222^
- Defeitos perfusionais não pareados ( *mismatch* ) e que não apresentam um claro padrão segmentar podem ser vistos em pacientes idosos em casos de TEP parcialmente resolvidas, mas não em casos de TEP aguda. Além disso, defeitos perfusionais não pareados ( *mismatch* ) são observados em outras etiologias pulmonares como câncer, linfadenopatia mediastinal, sequelas actínicas, pneumonite, fibrose e insuficiência cardíaca.
- Cintilografias pulmonares de ventilação e perfusão podem falhar na detecção de trombembolismo que causem apenas obstrução parcial vascular, com poucos efeitos hemodinâmicos. Tal alteração apresenta baixo significado clínico. Uma explicação para essa ocorrência é que o êmbolo não oclusivo, geralmente, está associado a outros sinais de TEP em outras regiões, levando ao diagnóstico correto. De qualquer forma, quando a presença de oclusão parcial é identificada (defeito perfusional segmentar evidente, na presença de ventilação normal), o estudo deve ser relatado como positivo para TEP.
- De modo geral, a ausência completa de perfusão unilateral, na presença de ventilação normal ipsolateral e sem qualquer outro *mismatch* no pulmão contralateral, não decorre de TEP. ^276 , 277^ Nesses casos, as tomografias computadorizadas de tórax costumam demonstrar a presença de outras patologias, como tumores ou outros processos mediastinais, anormalidades vasculares pulmonares congênitas ou aneurisma de aorta.

#### 7.1.11. O Futuro da Avaliação de EP por Técnicas de Medicina Nuclear

**Figura 29:**
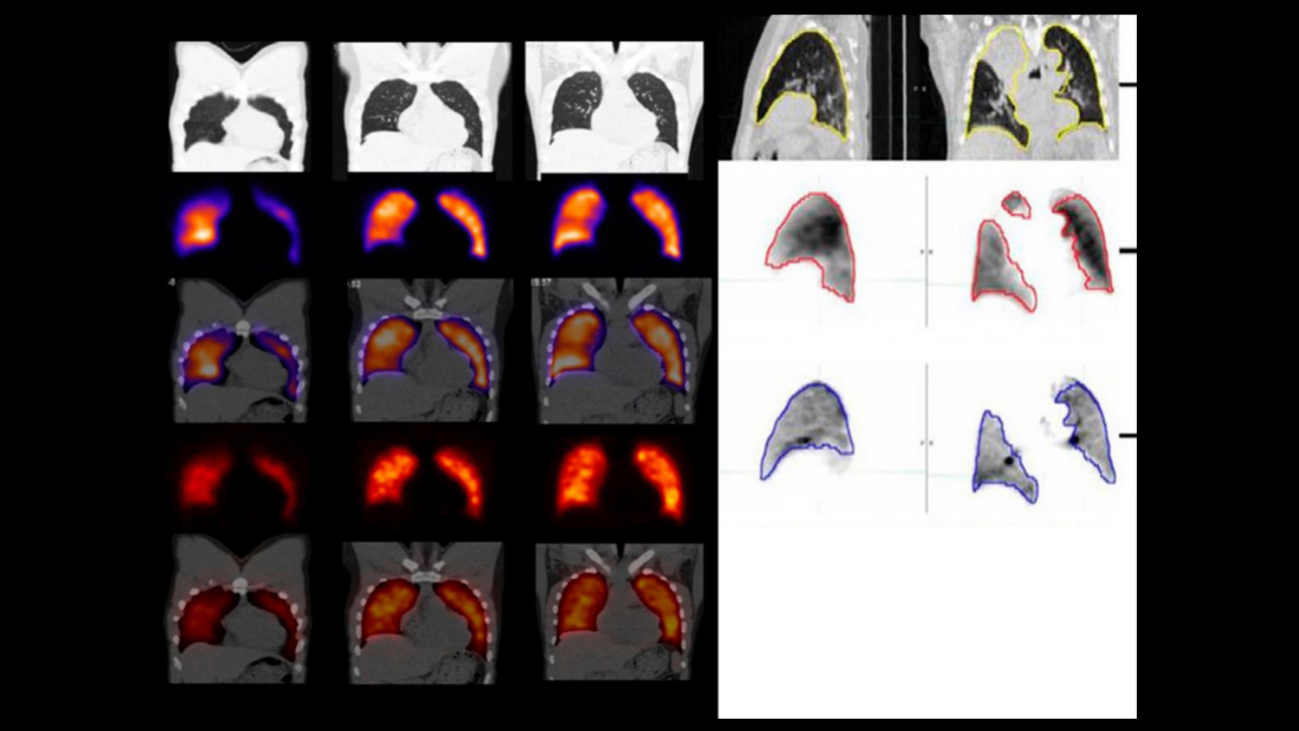
– À esquerda, série de imagens exemplificando uma cintilografia pulmonar V/Q Spect-CT normal, com uso de technegas/MAA marcados com 99mTc, mostrando de cima para baixo: TC baixa dose; ventilação Spect; fusão da ventilação/TC; perfusão Spect; e fusão perfusão/TC. À direita, série de imagens exemplificando um V/Q PET-CT normal, com uso de Galligas/MAA marcados com 68Ga de cima para baixo, cortes sagitais de fusão da ventilação/TC (esquerda) e perfusão/TC (direita). Adaptado de Le Roux Pyet et al.279e Bailey DL et al. 280

Ainda na década de 1970, o ^68^ Ga, um dos primeiros isótopos utilizados na avaliação de perfusão pulmonar, foi apresentado como o futuro da avaliação pulmonar pela medicina nuclear. Trata-se de um isótopo emissor de pósitron; portanto, sua utilização depende de equipamentos de PET e se daria na forma de microesferas marcadas com ^68^ Ga para avaliar a perfusão. Por outro lado, a ventilação seria avaliada de forma semelhante ao que se faz com o technegas: partículas marcadas com ^68^ Ga, o chamado *galligas* ^278^ ( Figura 29 ).

**Figura 30:**
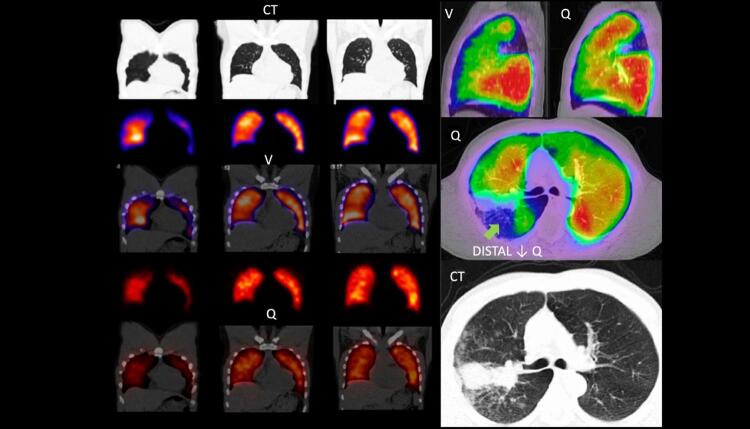
– À esquerda, série de imagens exemplificando uma cintilografia pulmonar V/Q SPECT-CT normal, com uso de technegas/MAA marcados com 99m Tc, mostrando, de cima para baixo: TC baixa dose, ventilação Spect, fusão da ventilação/CT, perfusão Spect e fusão perfusão/CT. À direita, série de imagens exemplificando um V/Q PET-CT, com uso de Galligas/MAA marcados com 68 Ga, de cima para baixo: cortes sagitais de fusão da ventilação/TC (a esquerda) e perfusão/TC (a direita); déficit concordante, imagem de fusão perfusão PET/CT mostrando déficit de perfusão (seta); e imagem de CT apenas, demonstrando tumor obstruindo brônquio, correspondendo ao defeito de ventilação e perfusão no PET.

Uma transição da V/Q Spect para PET-CT teria como vantagens características superiores de imagem intrínsecas da metodologia, como: maior sensibilidade do detector em anel, cobrindo 360 graus simultaneamente; melhor estatística de contagens; maior resolução espacial e temporal; redução do tempo de aquisição em torno de 10 min, podendo chegar a 5min com os novos PET/CT digitais. Outras vantagens seriam a disponibilidade comercial de sistemas de gatilhamento respiratório que permitam a redução de artefatos, especialmente nas bases pulmonares; permitir a possibilidade de múltiplos estudos ventilatórios, com ou sem broncodilatador, no mesmo dia, devido a curta meia-vida do gálio-68; e, claro, manutenção de características como simplicidade de execução, não invasivo, e sem contraindicações ou efeitos colaterais, relacionados ao uso de contraste (alergia e disfunção renal). Do ponto de vista técnico, não implica grandes dificuldades ou investimentos, pois os requisitos de radiofarmácia permanecem relativamente simples, com a utilização de um equipamento de Technegas existente, modificando apenas na síntese do macro agregado de albumina (MAA) e sua consequente marcação com Gálio-68 (cujos geradores são cada vez mais disponíveis, possibilitando uma adoção mais ampla deste isótopo) ao invés do ^99m^ Tc, mantendo uma dose de radiação muito próxima daquela de uma V/Q Spect/CT convencional ^108^ ( Figura 30 ).

Adaptado de Le Roux PY et al. ^279^ e Bailey DL et al. ^280^

Em 2018, Le Roux et al. ^281^ propuseram um protocolo para revisão sistemática, a metanálise da acurácia diagnóstica e os desfechos clínicos da V/Q SPECT, cujos resultados ainda não se encontram publicados e certamente colaborarão com mais evidência na adoção desta técnica.

#### 7.1.12. Algoritmo Clínico para Investigação de Pacientes com Suspeita de TEP

A avaliação da probabilidade clínica de TEP ajuda os médicos a escolherem o teste objetivo mais apropriado para diagnosticar ou excluir o diagnóstico ( Quadro 6 ). A medição do D-dímero (um produto de degradação do coágulo de fibrina reticulado) é amplamente utilizada para investigar pacientes com suspeita de trombembolismo venoso. O ensaio quantitativo do D-dímero, fundamentado em um método rápido de Elisa, apresenta alta sensibilidade (próximo aos 95%) ao trombembolismo venoso. ^22 , 23^ No entanto, o teste apresenta uma baixa especificidade (40%), porque o D-dímero pode ser aumentado em várias condições além do trombembolismo venoso, como infarto agudo do miocárdio, acidente vascular cerebral, inflamação, câncer ativo e gravidez. A especificidade cai com a idade e, em idosos, pode alcançar apenas 10%. ^120 , 214^ Consequentemente, um teste quantitativo negativo do D-dímero tem um alto valor preditivo negativo para o trombembolismo venoso. Os resultados dos estudos revelam que o risco de desenvolver TEP em pacientes com baixa probabilidade clínica, que não são tratados após um teste de D-dímero negativo é < 1% em três meses após a avaliação inicial. ^282^ Por outro lado, o baixo valor preditivo de um teste D-dímero quantitativo positivo não modifica a probabilidade pré-teste (clínica), sendo, portanto, clinicamente inútil. Evidencias recentes, no entanto, sugerem que níveis muito altos do D-dímero estão associados a um aumento de quatro vezes na probabilidade de TEP, ^30^ sendo importante na avaliação da carga da doença trombembólica e podendo ter significado prognóstico. ^27 , 29^

As estratégias diagnósticas variam com a estabilidade hemodinâmica dos pacientes:

**a) Pacientes estáveis:**

Com base no exposto, quando a probabilidade clínica de TEP for baixa e o D-dímero quantitativo for negativo, o diagnóstico é improvável e não são necessárias investigações adicionais ( Quadro 5 ). Quando a probabilidade clínica de TEP for baixa e o D-dímero quantitativo for positivo, poderão ser necessárias investigações adicionais para uma variedade de diagnósticos, incluído o TEP, sobretudo quando o nível do D-dímero estiver marcadamente elevado. Quando a probabilidade clínica for diferente de baixa, parece mais apropriado pular o teste do D-dímero e encaminhar o paciente diretamente para a técnica de imagem apropriada ( Quadro 5 ). Pode ser V/Q Spect ou angio-TC, dependendo da disponibilidade local, do conhecimento médico e da condição clínica do paciente. O V/Q Spect praticamente não tem contraindicações e produz uma carga de radiação substancialmente menor do que a angio-TC. ^22^

**b) Pacientes hemodicamente instáveis:**

Quando o paciente apresentar hipotensão grave ou choque cardiogênico ( Quadro 6 ), o ecocardiograma transtorácico pode permanecer como a técnica de imagem de primeira linha, já que possibilita detectar dilatação do coração direito e hipocinesia. Em raras circunstâncias, pode visualizar embolia dentro das cavidades cardíacas direitas ou na artéria pulmonar principal. A cintilografia pulmonar por perfusão é uma opção alternativa à angio-TC, pois pode mostrar rapidamente vários defeitos de perfusão segmentar ou lobar típicos da TEP aguda. ^120^ Quando houver suspeita de dissecção aguda da aorta torácica por conta de dor no peito, a angio-TC possibilita a avaliação desse diagnóstico diferencial.

Dada a necessidade de rapidez no diagnóstico e no tratamento desses pacientes, a estratégia empregada em uma instituição especifica deve ser adaptada à situação clinica específica e as circunstâncias locais. Quando o exame inicial sugerir uma ação maciça de TEP, outras iniciativas devem ser adaptadas à situação clínica, podendo ser ministrada terapia trombembolística.

#### 7.1.13. Algoritmo Diagnóstico

Quando suspeito, o TEP deve ser confirmado ou refutado para evitar os riscos de super e subtratamento, o que requer testes de imagem. Apenas as técnicas ótimas são recomendadas, ou seja, a angio-TC e o V/Q Spect. A modalidade de imagem usada dependerá da disponibilidade. ^120^ Ainda que a angio-TC esteja mais prontamente disponível, ela é contraindicada em um número substancial de pacientes, conforme mostrado no estudo Pioped II. ^217^ Nos dias atuais, o V/Q Spect raramente está disponível 24 horas/dia, 7 dias por semana. Consequentemente, esses dois métodos devem estar prontos para uso pelo menos em centros hospitalares terciários, visto que ambos são cruciais para algoritmos adequados no diagnóstico de TEP. Em cada centro, o algoritmo aplicado para o diagnóstico de TEP deve estar fundamentado em condições locais e, principalmente, na disponibilidade de V/Q Spect e angio-TC.

O V/Q SPECT, quando disponível, oferece vantagens consideráveis sobre as outras técnicas de imagem para o diagnóstico de TEP, como alta sensibilidade e especificidade para o diagnóstico de TEP, menor carga de radiação previsível e adequação ao acompanhamento de pacientes com TEP e sua história natural.

Os fluxogramas diagnósticos em pacientes hemodinamicamente estáveis e instáveis, de acordo com a probabilidade clínica de TEP, estão nos Quadros 5 e 6 . No Quadro 13 , estão apontadas as vantagens e desvantagens do uso da cintilografia pulmonar no diagnóstico do TEP.

**Quadro 13:** – Vantagens e desvantagens do uso da cintilografia pulmonar no diagnóstico do TEP

CINTILOGRAFIA PULMONAR	
VANTAGENS	DESVANTAGENS
Baixa exposição à radiação	Disponibilidade limitada
Pode ser realizada por gestantes	Não permite identificar com absoluta segurança a artéria ocluída
O radiofármaco utilizado não prejudica função renal	
Alta acurácia	
Procedimento coberto pelo SUS e pelo rol da ANS	
Permite avaliar TEP crônico	
Permite avaliar resolução de TEP agudo	

#### 7.1.14. Conclusões

- Cintilografia pulmonar V/Q é fortemente recomendada no diagnóstico de TEP, apresentando grande acurácia mesmo na presença de DPOC e pneumonia;
- Technegas é superior ao DTPA em pacientes com DPOC;
- Quando disponível, ^81m^ Kr é vantajoso;
- A dose de radiação deve ser reduzida ao máximo possível, sem prejuízo clínico na imagem (princípio Alara). De modo geral, devem ser usados 30MBq de ^99m^ Tc-aerosol para ventilação e 100-120 MBq de ^99m^ Tc-MAA para perfusão;
- Em pacientes gestantes, recomenda-se apenas a etapa de perfusão;
- A interpretação deve ser feita de forma holística; a interpretação probabilística é obsoleta;
- Critério fundamental para o diagnóstico de TEP é a presença de *mismatch* em mais de 1 subsegmento.
