# Supplementary material for: Diretriz Conjunta sobre Tromboembolismo Venoso – 2022
Source: Arq Bras Cardiol. 2022 Apr 7;118(4):797–857. [Article in Portuguese] doi: 10.36660/abc.20220213 (PMC9007000; doi:10.36660/abc.20220213)
Supplement: Translation

# Translation: Joint Guideline on Venous Thromboembolism – 2022

**Authors:** Ana Cristina Lopes Albricker, Cláudia Maria Vilas Freire, Simone Nascimento dos Santos, Monica Luiza de Alcantara, Mohamed Hassan Saleh, Armando Luis Cantisano, José Aldo Ribeiro Teodoro, Carmen Lucia Lascasas Porto, Salomon Israel do Amaral, Orlando Carlos Gloria Veloso, Ana Cláudia Gomes Pereira Petisco, Fanilda Souto Barros, Márcio Vinícius Lins de Barros, Adriano José de Souza, Marcone Lima Sobreira, Robson Barbosa de Miranda, Domingos de Moraes, Carlos Gustavo Yuji Verrastro, Alexandre Dias Mançano, Ronaldo de Souza Leão Lima, Valdair Francisco Muglia, Cristina Sebastião Matushita, Rafael Willain Lopes, Artur Martins Novaes Coutinho, Diego Bromfman Pianta, Alair Augusto Sarmet Moreira Damas dos Santos, Bruno de Lima Naves, Marcelo Luiz Campos Vieira, Carlos Eduardo Rochitte

**Affiliations:** 1 Brasil Centro Universitário de Belo Horizonte (UniBH), Belo Horizonte, MG – Brasil; 2 Brasil Universidade Federal de Minas Gerais (UFMG), Belo Horizonte, MG – Brasil; 3 Brasil Eccos Diagnóstico Cardiovascular Avançado, Brasília, DF – Brasil; 4 Brasil Hospital Quinta D’Or, Rede D’Or São Luiz, Rio de Janeiro, RJ – Brasil; 5 Brasil Instituto Dante Pazzanese de Cardiologia, São Paulo, SP – Brasil; 6 Brasil Hospital Barra D’Or, Rio de Janeiro, RJ – Brasil; 7 Brasil Ecovitall, Rio de Janeiro, RJ – Brasil; 8 Brasil PRENOTO Clínica Médica e Diagnóstica, Ribeirão Preto, SP – Brasil; 9 Brasil Universidade do Estado do Rio de Janeiro (UERJ), Rio de Janeiro, RJ – Brasil; 10 Brasil Hospital Samaritano, Rio de Janeiro, RJ – Brasil; 11 Brasil UnitedHealth Group (UHG), Rio de Janeiro, RJ – Brasil; 12 Brasil Angiolab Vitória, Laboratório Vascular, Rio de Janeiro, RJ – Brasil; 13 Brasil Rede Mater Dei de Saúde, Belo Horizonte, MG – Brasil; 14 Brasil Ecocenter Medicina Diagnóstica , Belo Horizonte, MG – Brasil; 15 Brasil Hospital das Clínicas da Faculdade de Medicina de Botucatu (FMB), Universidade Estadual Paulista (UNESP), Botucatu, SP – Brasil; 16 Brasil Clínica Fluxo de Cirurgia Vascular, São Bernardo do Campo, SP – Brasil; 17 Brasil Universidade Federal do Rio de Janeiro, Rio de Janeiro, RJ – Brasil; 18 Brasil Universidade Federal de São Paulo (UNIFESP), São Paulo, SP – Brasil; 19 Brasil RA Sabin Medicina Diagnóstica, Brasília, DF – Brasil; 20 Brasil Universidade Federal do Rio de Janeiro (UFRJ), Rio de Janeiro, RJ – Brasil; 21 Brasil Universidade de São Paulo (USP), Ribeirão Preto, SP – Brasil; 22 Brasil Instituto do Cérebro do Rio Grande do Sul (InsCer) da Pontifícia Universidade Católica do Rio Grande do Sul (PUCRS), Porto Alegre, RS – Brasil; 23 Brasil Medicina Nuclear Joinville São Marcos Diagnósticos, Joinville, SC – Brasil; 24 Brasil Hospital do Coração (Hcor), São Paulo, SP – Brasil; 25 Brasil Hospital das Clínicas da Faculdade de Medicina da Universidade de São Paulo, São Paulo, SP – Brasil; 26 Brasil Universidade Federal Fluminense (UFF), Niterói, RJ – Brasil; 27 Brasil Hospital Madre Teresa, Belo Horizonte, MG – Brasil; 28 Brasil Hospital Israelita Albert Einstein,São Paulo, SP – Brasil; 29 Brasil Instituto do Coração (InCor) da Faculdade de Medicina da Universidade de São Paulo (FMUSP),São Paulo, SP – Brasil; 30 Brasil DASA-ALTA, São Paulo, SP – Brasil

**Table:** 

Joint Guideline on Venous Thromboembolism – 2022	
The report below lists declarations of interest as reported to the SBC by the experts during the period of the development of these statement, 2021/2022.	
Expert	Type of relationship with industry
Adriano José de Souza	Nothing to be declared
Alair Augusto Sarmet Moreira Damas dos Santos	Nothing to be declared
Alexandre Dias Mançano	Nothing to be declared
Ana Cláudia Gomes Pereira Petisco	Nothing to be declared
Ana Cristina Lopes Albricker	Other relationships Funding of continuing medical education activities, including travel, accommodation and registration in conferences and courses, from the brazilian or international pharmaceutical, orthosis, prosthesis, equipment and implants industry: - Bayer: course on cardiovascular risk at INCOR.
Armando Luis Cantisano	Nothing to be declared
Artur Martins Novaes Coutinho	Nothing to be declared
Bruno de Lima Naves	Financial declaration A - Economically relevant payments of any kind made to (i) you, (ii) your spouse/partner or any other person living with you, (iii) any legal person in which any of these is either a direct or indirect controlling owner, business partner, shareholder or participant; any payments received for lectures, lessons, training instruction, compensation, fees paid for participation in advisory boards, investigative boards or other committees, etc. From the brazilian or international pharmaceutical, orthosis, prosthesis, equipment and implants industry: - Bayer: Xarelto. Other relationships Funding of continuing medical education activities, including travel, accommodation and registration in conferences and courses, from the brazilian or international pharmaceutical, orthosis, prosthesis, equipment and implants industry: - Bayer: Xarelto; Apsen: Dobeven; Servier: Daflon.
Carlos Eduardo Rochitte	Nothing to be declared
Carlos Gustavo Yuji Verrastro	Nothing to be declared
Carmen Lucia Lascasas Porto	Financial declaration A - Economically relevant payments of any kind made to (i) you, (ii) your spouse/partner or any other person living with you, (iii) any legal person in which any of these is either a direct or indirect controlling owner, business partner, shareholder or participant; any payments received for lectures, lessons, training instruction, compensation, fees paid for participation in advisory boards, investigative boards or other committees, etc. From the brazilian or international pharmaceutical, orthosis, prosthesis, equipment and implants industry: - Bayer: Xarelto; Pfizer: Eliquis; Boehringer Ingelhem: Pradaxa Phase III Studies. B - Research funding under your direct/personal responsibility (directed to the department or institution) from the brazilian or international pharmaceutical, orthosis, prosthesis, equipment and implants industry: - Bayer: Xarelto; Pfizer: Eliquis; Boehringer Ingelhem: Pradaxa Phase III Studies. Other relationships Funding of continuing medical education activities, including travel, accommodation and registration in conferences and courses, from the brazilian or international pharmaceutical, orthosis, prosthesis, equipment and implants industry: - Bayer: research.
Cláudia Maria Vilas Freire	Nothing to be declared
Cristina Sebastião Matushita	Financial declaration A - Economically relevant payments of any kind made to (i) you, (ii) your spouse/partner or any other person living with you, (iii) any legal person in which any of these is either a direct or indirect controlling owner, business partner, shareholder or participant; any payments received for lectures, lessons, training instruction, compensation, fees paid for participation in advisory boards, investigative boards or other committees, etc. From the brazilian or international pharmaceutical, orthosis, prosthesis, equipment and implants industry: - GE HealthCare: aula PET-CT PSMA e Aplication médica da CZT; R2/IBF: aula PET-CT 18-PSMA/1007; AMB: aula sobre Medicina Nuclear.
Diego Bromfman Pianta	Financial declaration A - Economically relevant payments of any kind made to (i) you, (ii) your spouse/partner or any other person living with you, (iii) any legal person in which any of these is either a direct or indirect controlling owner, business partner, shareholder or participant; any payments received for lectures, lessons, training instruction, compensation, fees paid for participation in advisory boards, investigative boards or other committees, etc. From the brazilian or international pharmaceutical, orthosis, prosthesis, equipment and implants industry: - GE Healthcare: PET-CT PSMA class in prostate cancer; R2/IBF: 18F-PSMA PET-CT class in prostate cancer.
Domingos de Morais	Nothing to be declared
Fanilda Souto Barros	Nothing to be declared
José Aldo Ribeiro Teodoro	Nothing to be declared
Marcelo Luiz Campos Vieira	Nothing to be declared
Márcio Vinícius Lins de Barros	Nothing to be declared
Marcone Lima Sobreira	Financial declaration A - Economically relevant payments of any kind made to (i) you, (ii) your spouse/partner or any other person living with you, (iii) any legal person in which any of these is either a direct or indirect controlling owner, business partner, shareholder or participant; any payments received for lectures, lessons, training instruction, compensation, fees paid for participation in advisory boards, investigative boards or other committees, etc. From the brazilian or international pharmaceutical, orthosis, prosthesis, equipment and implants industry: - Sanofi:Enoxaparina and VTE; Bayer: Rivaroxabana and VTE; Pfizer: Apixabana and VTE. B - Research funding under your direct/personal responsibility (directed to the department or institution) from the brazilian or international pharmaceutical, orthosis, prosthesis, equipment and implants industry: - Pfizer: Apixaban and Peripheral arterial disease; Venosan: Ready Wrap is chronic venous disease; Bayer: Rivaroxaban and VTE. Other relationships Funding of continuing medical education activities, including travel, accommodation and registration in conferences and courses, from the brazilian or international pharmaceutical, orthosis, prosthesis, equipment and implants industry: - Sanofi: VTE.
Mohamed Hassan Saleh	Nothing to be declared
Monica Luiza de Alcantara	Nothing to be declared
Orlando Carlos Gloria Veloso	Nothing to be declared
Rafael Willain Lopes	Nothing to be declared
Robson Barbosa de Miranda	Financial declaration A - Economically relevant payments of any kind made to (i) you, (ii) your spouse/partner or any other person living with you, (iii) any legal person in which any of these is either a direct or indirect controlling owner, business partner, shareholder or participant; any payments received for lectures, lessons, training instruction, compensation, fees paid for participation in advisory boards, investigative boards or other committees, etc. From the brazilian or international pharmaceutical, orthosis, prosthesis, equipment and implants industry: - Mindray Brasil: Diagnostic equipment. Other relationships Any economically relevant equity interest in companies in the healthcare or education industry or in any companies competing with or supplying to SBC: - Teaching Area: Vascular Ultrasound Course with Doppler.
Ronaldo de Souza Leão Lima	Nothing to be declared
Salomon Israel do Amaral	Nothing to be declared
Simone Nascimento dos Santos	Financial declaration A - Economically relevant payments of any kind made to (i) you, (ii) your spouse/partner or any other person living with you, (iii) any legal person in which any of these is either a direct or indirect controlling owner, business partner, shareholder or participant; any payments received for lectures, lessons, training instruction, compensation, fees paid for participation in advisory boards, investigative boards or other committees, etc. From the brazilian or international pharmaceutical, orthosis, prosthesis, equipment and implants industry: - Jonhson and Abbott: Intracardiac echo.
Valdair Francisco Muglia	Nothing to be declared

Content

Introduction 801

1. Pathophysiology and Clinical and Laboratory Diagnosis of Venous Thromboembolism 802

1.1. Introduction 802

1.2. Pathophysiology 802

1.3. Clinical and laboratory diagnosis 802

**1.3.1. Measurement of D-dimer** 803

2. Echocardiographic Changes in Pulmonary Embolism 805

2.1. Introduction 805

2.2. Echocardiography in Low-risk Pulmonary Embolism (PE) 805

2.3. Echocardiography in High-risk Pulmonary Embolism (PE) 805

2.4. Recommendations 815

3. Intravascular Ultrasound in Deep Vein Thrombosis Diagnosis 815

3.1. Ultrasound Signs of Vein Thrombosis: B-mode and Doppler 815

**3.1.1. Imaging Methodology – Technical Aspects** 815

3.2. IVUS Protocols in DVT 815

3.3. Test Protocols 815

**3.3.1. Complete DuplexVUS** 815

**3.3.2. Complete Lower Extremity Venous Compression US** 823

**3.3.3. Extended Compression US (Three-point)** 823

**3.3.4. Two-point Compression US** 823

3.4. Differential Diagnosis 826

3.5. Recommendations 826

3.6. How to Write Imaging Test Reports 828

4. Vascular ultrasound in the diagnosis of recurrent deep vein thrombosis and post-thrombotic syndrome 828

4.1. Recurrent venous thrombosis 828

4.2. Ultrasonic Diagnosis of Recurrent Thrombosis 829

4.3. Post-thrombotic Syndrome 829

**4.3.1. Pathophysiology** 830

**4.3.2. Clinical Manifestations** 830

**4.3.3. Diagnosis** 830

5. Follow-up Protocols with Intravascular Ultrasound after Deep Vein Thrombosis 833

5.1. Introduction 833

5.2. Recanalization 833

5.3. Recurrent Thrombosis 833

5.4. Valve Insufficiency 833

5.5. Discussion 833

**5.5.1. Initial Negative VUS** 833

**5.5.2. Proximal vs. Distal Thrombosis** 833

**5.5.3. Recurrent Deep Vein Thrombosis** 835

5.4. Recommendations 835

6.1. CT angiography 835

**6.1.1. Imaging Technique and Protocols** 836

6.2. Dual-energy computed tomography 837

6.3. Older Generations of CT Scanners 837

6.4. Diagnostic Criteria 837

6.5. Prognostic Criteria 837

6.6. Contraindications and Special Situations 837

6.7. MR Angiography 840

6.8. Digital Subtraction Angiography 840

6.9. Recommendations 840

7. Lung scintigraphy 841

7.1. Evidence 841

**7.1.1. Introduction** 841

**7.1.2. Evidence Regarding Imaging Interpretation Criteria** 841

**7.1.3. Diagnostic Accuracy** 842

**7.1.4. Indications for Lung Scintigraphy Due to Limitations of CT Pulmonary Angiography** 843

**7.1.5. Protocol** 843

**7.1.6. Image Reconstruction** 844

**7.1.7. Interpretation** 844

**7.1.8. Chronic Pulmonary Embolism** 845

**7.1.9. Additional Diagnostic Findings** 845

**7.1.10. Pitfalls in the Interpretation of V/Q Studies** 845

**7.1.11. The future of Nuclear Medicine Evaluation of PE** 845

**7.1.12. Clinical Algorithm for the Investigation of Patients with Suspected PE** 846

**7.1.13. Diagnostic Algorithm** 847

**7.1.14. Conclusions** 848

References 848

## Introduction

Venous thromboembolism (VTE) manifests as pulmonary embolism (PE) and/or deep vein thrombosis (DVT), being part of the same disease spectrum and presenting the same risk factors. It is the third most common cause of acute cardiovascular syndrome worldwide, being potentially life-threatening.^1 , 2^

In Brazil, according to data from the Ministry of Health collected between 2010 and 2021, the number of hospitalizations for VTE exceeded 520,000, with a total of more than 67,000 deaths from 2010 to 2019.^3^

It has a high mortality rate. Approximately 34% of affected patients die suddenly or within a few hours of the first manifestation, that is, even before receiving any type of treatment.

Almost two-thirds of the cases of VTE manifest as DVT alone, mostly as proximal DVT of the lower extremities, and one-third as PE.^2^

In the literature, several studies have associated the presence of VTE with climate variation. In Brazil, Ohki et al.^4^reported a higher incidence of VTE in the southern states, where temperatures are lower. These states have a mean incidence of VTE of 2.86 cases per 100,000 population.^4^

This clinical syndrome exponentially increases with age, even with the implementation of prevention strategies.^5^Women are most commonly affected in their youth, especially in the postpartum period. During pregnancy, tobacco use, thrombophilia and/or a history of VTE increase the risk in this group of patients. Other conditions, such as prolonged immobility, obesity, cancer, major surgery with prolonged anesthesia, multiple trauma, lower-extremity varicose veins, hormone replacement therapy, and cardiovascular diseases, are considered at risk for VTE, although it has been recognized that there is variation in risk prediction between these causes.^2^

Ethnicity is considered a risk factor for VTE. The incidence of VTE is significantly higher among White and African American people and lower among Hispanic and Asian people. Overall, approximately 25%-50% of patients presenting with a first episode of VTE have an idiopathic condition, without an easily identifiable risk factor. Early mortality in VTE is strongly associated with pulmonary involvement, in the form of PE, in addition to advanced age, cancer and underlying cardiovascular disease.^2^

The recurrence rate is relevant, and non-fatal VTE may have sequelae such as pulmonary hypertension in cases of chronic PE and post-thrombotic syndrome in DVT. Notably, there has been a reduction in deaths due to better diagnostic and therapeutic management after the publication of international position statements and guidelines on the topic.^4^

Diagnosis of VTE may not be straightforward. Therefore, it is necessary to focus on the diagnostic approach to PE and DVT, as failure to correctly diagnose the condition can be fatal or lead to permanent comorbidities.

In this context, the Department of Cardiovascular Imaging (DIC/SBC), the Brazilian College of Radiology (CBR), the Brazilian Society of Angiology and Vascular Surgery (SBACV), and the Brazilian Society of Nuclear Medicine (SBMN) jointly developed this document. The purpose is to address clinical and diagnostic aspects aiming to create a single recommendation for the 4 societies, which should serve as a source of information for Brazilian physicians and as a standard for the clinical, laboratory, and imaging diagnosis of VTE. The prophylactic and therapeutic approach to VTE is not within the scope of this document.

This recommendation was developed based on guidelines, on population-based studies and case reports published in the literature, and on the expertise of the participating members. Each work group compiled the data, drafted the writings, and then submitted the revised text for final adjustments. The most controversial issues were discussed at a meeting attended by the majority of the members. Corrections were made to the original text if necessary.

This recommendation is aimed at clinicians or surgeons and intends to provide the best information on the diagnostic approach to VTE, offering data on the accuracy of diagnostic tools used in daily practice. For imaging physicians, a detailed description of the imaging protocols can help them make a more accurate diagnosis, thus contributing to a better clinical management of suspected or confirmed cases.

## 1. Pathophysiology and Clinical and Laboratory Diagnosis of Venous Thromboembolism

### 1.1. Introduction

The spectrum of venous thromboembolism (VTE) comprises deep vein thrombosis (DVT) and pulmonary embolism (PE). Thromboembolic disease is the third most common acute cardiovascular disease, after ischemic heart syndromes and stroke. There is a broad spectrum of clinical manifestations for these diseases, from clinically silent to massive embolism, leading to death.^5^About one-third of all cases of PE are fatal, and cancer is one of the many disease states associated with an increased risk of thromboembolic disease. Approximately two-thirds of the cases of VTE are DVT, 85%-90% occurring in the lower extremities, and one-third are PE.^6 , 7^In this context, we must always remember that VTE is a serious but preventable disease.

### 1.2. Pathophysiology

#### A) Deep vein thrombosis (DVT):

DVT is a common and potentially fatal condition, with PE as the main complication. It can be considered the first cause of avoidable in-hospital morbidity and mortality. In addition, it can also lead to post-thrombotic syndrome (PTS) as a chronic complication, which has an important socioeconomic impact. These complications can occur despite proper diagnosis and therapy, but early diagnosis and prompt initiation of the correct treatment can reduce their potential deleterious effects.

DVT can occur in both inpatients and outpatients. The symptoms are nonspecific, varying from a completely asymptomatic to a catastrophic condition, such as in phlegmasia cerulea dolens (PCD). PCD is characterized by complete occlusion or massive subocclusion of the limb draining veins (iliofemoral system) and collaterals, which may extend to the microcirculation. Venous hypertension reaches a level that can obstruct arterial inflow.^7 , 8^The high hydrostatic pressure and interstitial fluid sequestration leads to the formation of hardened edema in the entire limb, which can accumulate 3 to 5 liters in volume and lead to the collapse of arterioles, skin blisters due to tissue ischemia, purpura/ecchymosis, cyanosis, loss of distal pulses, circulatory collapse, and hypovolemic shock.^9 , 10^

Importantly, the diagnosis of DVT begins with the clinical history. Attention should be paid to any past medical history that may potentiate or predispose the patient to an increased risk of developing venous thromboembolic phenomena, as postulated by Virchow:

**- BLOOD FLOW CHANGES (stasis):** age, immobility ≥3 days or physical inactivity such as in the postoperative period, stroke, knee or hip fracture, major general surgery (>45 min, the risk rises with increasing procedure duration), major trauma, spinal cord injury with limb paresis, immobilization of extremities with casts or orthoses, long-haul travel in a confined space, pregnancy, congestive heart failure, varicose veins, COPD, and burns, among others.^11^
**- ENDOTHELIAL DAMAGE (trauma):** advanced age (after 40 years of age, the risk doubles with each decade), smoking, known thromboembolic history, trauma, surgery, and central venous catheters, among others.^11 , 12^
**- HYPERCOAGULABILITY:** cancer and/or their treatment, inherited or acquired thrombophilia, obesity, use of estrogen-containing oral contraceptives and hormone replacement therapy, pregnancy, smoking, acute infectious diseases, nephrotic syndrome, and inflammatory bowel disease, among others.^9 , 11 , 13 , 14^

## 1.3. Clinical and laboratory diagnosis

Once the epidemiological risk for DVT has been identified, and it is mandatory, physical examination should be performed. The clinical diagnosis of DVT has low sensitivity and specificity, as only 20%-40% of patients with suggestive clinical features have the disease confirmed. The most common signs and symptoms of DVT are pain and edema. Despite the low accuracy of clinical diagnosis, it is important to note that, at first, any asymmetric swelling in the lower extremities should be considered until the diagnostic evaluation is completed.

However, it should be also taken into account that, in a considerable number of cases (20%-50%), the patient may present with extensive DVT (even proximal) without any suggestive symptoms. Clinical suspicion is essential for the diagnosis in these cases, but one should keep in mind that the clinical diagnosis does not have satisfactory sensitivity/specificity. Therefore, the final diagnosis must be confirmed by complementary tests. Vascular Doppler ultrasound is the method of choice to confirm DVT due to its high accuracy, being easy to perform and harmless with good reproducibility. However, the availability of this diagnostic modality is limited in smaller medical centers and outpatient clinics as well as during night and weekend shifts.^15^

DVT is classified as proximal when it involves the femoral and/or popliteal veins, with or without the involvement of other leg veins, and as distal when it involves the deep infrapatellar veins.^16 , 17^Proximal DVT has the greatest potential for embolism. Other classifications that can determine the severity of DVT are based on the degree of thrombus extension. More extensive thrombi usually have greater consequences because they carry venous hypertension and the degree of partial and complete luminal obstruction. The degree of luminal narrowing, as well as its location and extension, will determine the clinical severity and prognosis of venous thrombosis.

In 1997, in an attempt to simplify the diagnostic approach to these patients, Wells et al.^18^developed a clinical prediction model to classify patients for DVT risk. The method proved to be feasible and useful in several studies when combined with complementary noninvasive tests. The same group implemented modifications in 2003, facilitating understanding for clinicians.^6^Several specialty and consensus societies recommend the use of clinical prediction models to estimate the probability of a DVT diagnosis before performing a vascular ultrasound (VUS) examination.^19^Among these tools, the most used one is the combination of Wells score and serum D-dimer levels.^7^( Chart 1 ).

**Chart 1:** – Wells clinical model for predicting deep vein thrombosis (DVT) 6

Clinical Findings	Score
Active cancer OR cancer treated within the previous 6 months.	1
Paresis, paralysis, or recent immobilization of the lower extremities.	1
Recently bedridden for 3 days OR major surgery within the previous 4 weeks.	1
Localized tenderness along the distribution of the deep venous system.	1
Entire leg swollen.	1
Calf swelling at least 3 cm larger than that on the contralateral side (measured 10 cm below the tibial tuberosity).	1
Pitting edema (positive Godet sign) confined to the symptomatic leg.	1
Collateral superficial veins (nonvaricose).	1
Previously documented DVT.	1
Presence of more likely differential diagnosis: lymphedema, cellulitis, joint changes, superficial thrombophlebitis, muscle rupture, Baker’s cyst.	-2

- Simplified score: ≥ 2 DVT likely; ≤1 DVT unlikely
- The diagnostic accuracy of Wells score depends on the population assessed (outpatient x inpatient), extent of DVT (proximal x distal), and degree of probability (low x moderate x high), being more accurate in outpatients with proximal DVT who have a high probability score (>2). Silveira et al., in a population-based study, obtained an overall diagnostic accuracy of 0.56 (AUC – area under the curve) for inpatients. In outpatients, the effectiveness of the score can range from 11.9% to 79.5%.^6 , 20 , 21^

### 1.3.1. Measurement of D-dimer

Measurement of D-dimer (a degradation product of cross-linked fibrin clot) is widely used in the investigation of patients with suspected VTE.^22 , 23^The quantitative D-dimer assay, based on a rapid ELISA test, has high diagnostic sensitivity (close to 95%). However, the assay has low specificity (40%) because D-dimer levels may be increased in several conditions other than VTE, such as in acute myocardial infarction, stroke, inflammation, active cancer, and pregnancy. Specificity also decreases with age, and it can be as low as 10% in older adults. In a systematic review, the use of an adjusted cutoff value for patients over 50 years of age in order to rule out the presence of DVT (patient age in years [above 50] x 10 in µg/L) appeared to be as safe as the standard cutoff value, which was pointed out as a recommendation in the latest 2019 European guideline.^24 , 25^

Consequently, a negative quantitative D-dimer test has a high negative predictive value for VTE. The results of the studies reveal that the risk of development of PE in patients with low clinical probability who are not treated after a negative D-dimer test is <1% within 3 months of the initial evaluation. However, because of its low predictive value, a positive quantitative D-dimer test does not modify the pretest (clinical) probability and is therefore clinically useless. A highly sensitive negative D-dimer test combined with a low pretest probability can exclude DVT.^26^

D-dimer levels can also vary depending on the number of veins involved, thrombus extension and volume. More extensive proximal thromboses have higher D-dimer levels than those confined to the calf veins, which may be an important factor when assessing the burden of thromboembolic disease.^22 , 27 - 29^Recent evidence also suggests that very high D-dimer levels are associated with a 4-fold increase in the probability of PE.^22 , 30^However, when the clinical probability is high, regardless of D-dimer levels, VUS is mandatory to confirm or rule out the presence of DVT^31^( Chart 2 ).

**Chart 2:** – Diagnostic flowChart for the study of deep vein thrombosis (DVT)

### B) Pulmonary embolism (PE):

PE is defined as the obstruction of one or more pulmonary arteries. In most cases, it is caused by blood clots that, most often, arise from DVT of the lower extremities and reach the pulmonary arteries.

Clinical signs of PE are nonspecific, such as dyspnea, chest pain, hemoptysis, and syncope or presyncope. In some cases, it may be asymptomatic and discovered incidentally. Syncope appears to be present in approximately 17% of cases and is associated with a high prevalence of hemodynamic instability and right ventricular failure. PE with hemodynamic instability is infrequent, but it may be suggestive of massive impairment of the pulmonary circulation.

If PE is suspected, it must be confirmed or ruled out to avoid the risk of overtreatment or undertreatment. The Wells or Geneva clinical risk scores are often used to classify the pretest probability.

The diagnostic accuracy of Wells scores for the diagnosis of PE was established by a meta-analysis of 11 studies, in which sensitivity ranged from 63.8% to 79.3% and specificity, from 48.8% to 90%, with an AUC of 0.778. For the Geneva score, however, sensitivity ranged from 55.3% to 73.6% and specificity, from 48.8% to 90.0%, with an AUC of 0.693.^32^

These scores, when combined with the measurement of D-dimer, as described earlier, have important implications for the diagnostic workup and for the indication of imaging tests ( Charts 3 and 4 ).

**Chart 3:** – Wells clinical model for predicting pulmonary embolism 33

VARIABLES	SIMPLIFIED SCORE
Clinical signs of DVT	3
Heart rate > 100 bpm	1.5
Immobilization or recent surgery	1.5
Previous DVT or PE	1.5
Hemoptysis	1
Cancer	1
Alternative diagnosis less likely than PE	3
PE likely > 4 and PE unlikely ≤ 4	

**Chart 4:** – Revised Geneva clinical model for predicting pulmonary embolism (PE). Adapted from Le Gal et al. 34

VARIABLES	CLINICAL POINTS EVALUATED	
	ORIGINAL	SIMPLIFIED
Previous PE or DVT	3	1
HEART RATE		
75 – 94 bpm	3	1
≥ 95 bpm	5	2
Surgery or fracture ≤ 1 month	2	1
Hemoptysis	2	1
Active cancer	2	1
Unilateral lower-limb pain	3	1
Pain on lower-limb deep venous palpation and unilateral edema	4	1
Age > 65 years	1	1
**CLINICAL PROBABILITY**		
**3-level score**		
Low	0 - 3	0 - 1
Intermediate	4 - 10	2 - 4
High	≥ 11	≥ 5
**2-level score**		
Unlikely	0 - 5	0 - 2
Likely	≥ 6	≥ 3

In the assessment of the clinical probability of PE, diagnostic strategies will depend on the patient’s hemodynamic stability. In addition to clinical features and D-dimer, complementary techniques recommended for the diagnosis of PE include computed tomography (CT) pulmonary angiography, V/Q SPECT scintigraphy, and echocardiography ( Charts 5 and 6 ). Each of these techniques and the role of echocardiography in the diagnostic flowchart, when PE has hemodynamic repercussions, hypotension, or shock, will be discussed later.

**Chart 5:** – Diagnostic flowChart for the assessment of suspected pulmonary embolism (PE) without hemodynamic repercussions

**Chart 6:** – Diagnostic algorithm for acute pulmonary embolism (PE) in hemodynamically unstable patients

## 2. Echocardiographic Changes in Pulmonary Embolism

### 2.1. Introduction

The usefulness of echocardiography (transthoracic and/or transesophageal) in acute pulmonary embolism (PE) lies in the investigation of right ventricular (RV) pressure overload (due to increased pulmonary vascular resistance, with consequent increase in RV afterload) and in functional assessment of the RV. In most cases, patients with thromboembolic episodes without hemodynamic repercussions have normal test results. In hemodynamically unstable patients, echocardiography plays an important supporting role in the stages of diagnosis and noninvasive monitoring of treatment response.

### 2.2. Echocardiography in Low-risk Pulmonary Embolism (PE)

Echocardiography is not a mandatory test in the diagnostic routine for hemodynamically stable patients with suspected PE in diagnostic algorithms.^1^The negative predictive value of echocardiography ranges from 40%-50%, and a normal result does not exclude PE.^35 - 37^Despite not contributing to the diagnosis, it represents an important tool for prognostic discrimination: the absence of changes in RV size or function indicate a good prognosis.^38 - 41^The test is also important to define differential diagnoses of acute dyspnea.

In the study of the RV, with its unique crescent shape, there are technical difficulties inherent in the asymmetric geometry of the cavity. However, the technique for a comprehensive examination was standardized in 2015 in an updated joint document from the American Society of Echocardiography and the European Association of Cardiovascular Imaging.^42^The general recommendations define the essential windows and views that provide the images required to obtain all the necessary data for quantification:

- Left parasternal, long- and short-axis;
- Apical 4-chamber;
- RV-focused apical 4-chamber;
- Left parasternal RV inflow;
- Subcostal.

Chart 7 provides the recommendations for measurement of right cavity dimensions and the parameters for RV systolic function.

**Chart 7:** – Recommendations for measurement of right cavity dimensions and the parameters for right ventricular (RV) systolic function.

LINEAR MEASUREMENTS OF THE RIGHT VENTRICULAR (RV) INFLOW TRACT	
	**RVd1 =** basal RV linear diameter (maximal transversal dimension in the basal one-third of RV inflow at end-diastole in the RV-focused view). NV = 33 ± 4 mm25-41 **RVd2 =** mid-cavity RV linear diameter (transversal RV dimension in the middle third of RV inflow, approximately halfway between the maximal basal diameter and the apex, at the level of papillary muscles at end-diastole. NV = 27 ± 4 mm^19-35^
**LINEAR MEASUREMENTS OF THE RIGHT VENTRICULAR (RV) OUTFLOW TRACT**	
****	**A)** Proximal RV outflow diameter (parasternal long-axis view) = linear dimension measured from the anterior RV wall to the interventricular septal-aortic junction at end-diastole. NV = 25 ± 2.5 mm^20-30^
****	**B)** Proximal RV outflow diameter (parasternal short-axis view) = linear dimension measured from the anterior RV wall to the aortic valve at end-diastole. NV = 28 ± 3.5 mm^21-35^
****	**C)** Distal RV outflow diameter (parasternal short-axis view) = linear transversal dimension measured just proximal to the pulmonary valve at end-diastole. NV = 22 ± 2.5 mm^17-27^
**LINEAR MEASUREMENTS OF RV WALL THICKNESS**	
****	Linear measurement of RV wall thickness (either by one-dimensional or 2D echocardiography) is performed at end-diastole, below the tricuspid annulus at a distance approximating the length of anterior tricuspid leaflet (fully open and parallel to the RV free wall). NV = 3 ± 1 mm^1-5^
**MEASUREMENTS OF RV VOLUME BY 3D ECHOCARDIOGRAPHY**	
****	RV size should be measured by conventional 2D echocardiography using multiple acoustic windows. However, in laboratories with experience in 3D echocardiography, 3D measurement of RV volumes is recommended. NV for RV EDV: Men 61 ± 13 mL/m^235-87^ Women 53 ± 10.5 mL/m^232-74^ NV for RV ESV: Men 27 ± 8.5 mL/m^210-44^ Women 22 ± 7 mL/m^28-36^
**RV GLOBAL SYSTOLIC FUNCTION ASSESSED BY FRACTIONAL AREA CHANGE (FAC)**	
	RV FAC (%) = 100 X (EDA – ESA)/EDA RV-focused apical 4-chamber view. Manual tracing of RV endocardial border from the lateral tricuspid annulus along the free wall to the apex and back to medial tricuspid annulus, along the interventricular septum at end-systole (ESA) and at end-diastole (EDA). Trabeculations, papillary muscles, and moderator band are included in the cavity area measurement. NV = 49 ± 7% (>35%)
**RV GLOBAL SYSTOLIC FUNCTION ASSESSED BY 3D ECHOCARDIOGRAPHY EJECTION FRACTION**	
****	Fractional RV volume change by 3D echocardiography [RV EF (%) = 100 X (EDV-ESV)/EDV]. When performed correctly, it correlates with ejection fraction calculated by cardiac magnetic resonance. In experienced laboratories, 3D measurement of RV ejection fraction is recommended. NV = 58 ± 6.5% (>45%)
**RV LONGITUDINAL SYSTOLIC FUNCTION ASSESSED BY TAPSE**	
****	Tricuspid annular longitudinal excursion measured by M-mode between end-diastole and peak systole. RV-focused apical 4-chamber view for proper alignment of M-mode cursor with the direction of RV longitudinal excursion (lateral tricuspid annulus). NV = 24 ± 3.5 mm (>17mm)
**RV LONGITUDINAL SYSTOLIC FUNCTION ASSESSED BY PULSED TISSUE DOPPLER S WAVE**	
****	Peak systolic velocity of tricuspid annulus assessed by pulsed-wave tissue Doppler imaging (cm/s), obtained from the RV-focused apical 4-chamber view. It is important to keep the basal segment and the annulus aligned with the Doppler cursor to avoid velocity underestimation. NV = 14.1 ± 2.3 cm/s (>9.5 cm/s)
**RV LONGITUDINAL SYSTOLIC FUNCTION ASSESSED BY GLOBAL LONGITUDINAL STRAIN**	
	RV longitudinal strain should be measured in the RV-focused apical 4-chamber view. The panel demonstrates RV global longitudinal strain of the 6 segments (3 free wall and 3 septal segments); the values are averaged. RV longitudinal strain is often measured by software not dedicated to that chamber. Currently, there are software versions dedicated to the RV, which should be preferred as they provide data on RV free wall strain and on the strain that incorporates the interventricular septum. NV: 29 ± 4.5 (20% in absolute value)
**RIGHT ATRIAL (RA) 2D VOLUMETRIC MEASUREMENTS**	
	The recommended parameter to assess RA size is RA volume, calculated using single-plane disk summation (2D) techniques in a dedicated apical 4-chamber view. NV: Men 25 ± 7 mL/cm^2 18-32^ Women 21 ± 6 mL/cm^2 15-27^
**MEASUREMENT OF THE INFERIOR VENA CAVA (IVC) DIMENSIONS**	
	In the subcostal view with the patient in the supine position at 1-2 cm from the junction with the RA (perpendicular to the long axis). The “collapsibility index (CI)” (% decrease in IVC diameter during inspiration) correlates with RA pressure. IVC ≤ 21 mm and CI > 50% = normal RA pressure (3 mm Hg, ranging from 0-5 mm Hg). IVC ≤ 21mm and CI < 50% = intermediate RA pressure (8 mm Hg, ranging from 5-10 mm Hg). zIVC > 21 mm and CI < 50% = high RA pressure (15 mm Hg, ranging from 10-20 mm Hg).


*TSVD: trato de saída do VD; ASC: área de superfície corporal; VD: ventrículo direito; AD: átrio direito; VCI: veia cava inferior; VN: valor de normalidade.*

### 2.3. Echocardiography in High-risk Pulmonary Embolism (PE)

Echocardiography is mandatory for all hemodynamically unstable patients with clinically suspected PE, as it can reliably detect changes in the right cavities that reflect a sudden and severe increase in pulmonary vascular resistance (RV afterload), responsible for dysfunction and hypotension. In these patients, normal test results definitively rule out the hypothesis of massive PE.^39 , 43^Conversely, the detection of changes corresponding to RV pressure overload and dysfunction (in the absence of other obvious causes of differential diagnosis) allows the diagnosis of massive PE and emergency reperfusion therapy, even if chest CT angiography or ventilation and perfusion scintigraphy cannot be performed.^44^

The only pathognomonic echocardiographic finding of PE is the presence of mobile thrombi in the right cavities and/or pulmonary trunk or branch arteries ( Figure 1 ). This finding is associated with high early mortality^45 - 49^and occurs in only 4% of overall cases of PE, reaching an 18% prevalence in patients with PE in intensive care units.^50 , 51^

**Figure 1:** – A-C: direct visualization of thrombus in the right cavities and left pulmonary artery. Thr: thrombus; Ao: aorta; LP: left pulmonary artery; RP: right pulmonary artery.

Key changes related to severe acute PE^52^are:

- RV dilatation;
- RV dysfunction;
- Pulmonary pressure overload. It occurs in 30%-40% of patients with PE and indicates worse prognosis.^53 - 55^

When ventricular dilatation ( Figure 2 ) is associated with the 60/60 sign – combination of pulmonary ejection acceleration time of <60 ms and tricuspid valve peak systolic gradient <60 mm Hg –, or with McConnell sign – hypokinesia of basal and mid segments of the RV free wall and normokinesia of the apical segment ( Figure 3 ) –, the positive predictive value for massive PE is high.^56^However, the 60/60 sign is present in only 12%, and McConnell sign in 20% of unselected patients. Another sign of increased RV afterload is reduced pulmonary acceleration time and the presence of mid-systolic deceleration ( Figure 4 ). Signs of RV pressure overload help to differentiate hypokinesia or akinesia of the free wall due to PE from that caused by acute right ventricular infarction (which may mimic McConnell sign).^52 , 57^

**Figure 2:** – Right ventricular (RV) pressure overload and/or dysfunction A: straightening of the interventricular septum with RV dilatation in the parasternal cross-sectional view; B: RV dilatation assessed in the apical view by the RV:LV ratio at end-diastole (>0.6 mild; 1-2 important; >2 severe); C: equivalent to RV:LV ratio assessed by CT angiography. RV: right ventricle; LV: left ventricle; RA: right atrium; LA: left atrium.

**Figure 3:** – McConnell sign. Presence of right ventricular dilatation, free-wall hypokinesia, and preserved apical contractility. A: diastole; B: systole; C: longitudinal strain of the right ventricle (RV); left ventricle (LV); right atrium (RA); and left atrium (LA).

**Figure 4:** – Hemodynamic profile of right ventricular outflow tract (RVOT) caused by increased RV afterload: reduction in acceleration time and presence of mid-systolic deceleration (notch – white arrow).

Patients with PE may also have reduced tricuspid annular plane systolic excursion (TAPSE).^58 , 59^Tissue Doppler and parietal strain of the RV have low sensitivity as isolated findings in patients with acute PE.^60 , 61^In patients with suspected acute PE but with increased RV free wall thickness or tricuspid valve regurgitant jet velocities above >3.8 m/s or tricuspid valve peak systolic gradient >60 mm Hg, we should include differential diagnosis with chronic thromboembolic pulmonary hypertension.^38 , 62^

The 3D echocardiography can be used in laboratories with experience in the technique for an evolutionary analysis of patients with massive PE treated with thrombolysis. Figures 5 and 6 illustrate a case of massive PE, with significant hemodynamic repercussions, treated with a thrombolytic agent, progressing to an improvement in RV ejection fraction and a significant reduction in right cavity volumes.

**Figure 5:** – Follow-up3D echocardiographic analysis of the right ventricle (RV), with measurement of RV end-diastolic volume (EDV), end-systolic volume (ESV), and ejection fraction (EF). Patient with submassive pulmonary embolism at 3 time points: A: pre-thrombolysis (RV EDV = 165 mL, RV ESV = 115 mL, RV EF = 30%); B: day 2 after thrombolysis (RV EDV = 137 mL, RV ESV = 82 mL, RV EF = 40%); C: day 5 after thrombolysis (RV EDV = 113 mL, RV ESV = 62 mL, RV EF = 47.8%).

**Figure 6:** – Figure 6 – Follow-up images analysis of the right ventricular (RV) free-wall longitudinal strain in absolute values and at 3 time points: A: pre-thrombolysis; B: day 2 after thrombolysis; C: day 5 after thrombolysis with normalized strain values.

In the beginning of 2020, the new coronavirus pandemic (COVID-19) added a crucial new role for echocardiography. So far, findings reported in the literature indicate that the virus causes a significant increase in the risk of venous thrombosis (translated by increased D-dimer) and, consequently, in the occurrence of PE. Conversely, it seems to cause local thrombosis in pulmonary microcirculation vessels, leading to RV pressure overload. Both factors can result in RV dilatation and dysfunction, measured by various parameters, as described in Chart 8 . These patients develop acute respiratory failure requiring invasive positive pressure ventilation, which also contributes to increased RV afterload. In these patients, echocardiographic changes are very similar (RV dilatation and dysfunction with pulmonary hypertension), and it is not always possible to differentiate the predominant cause. In a recent multicenter study of 870 patients hospitalized for COVID-19, RV free-wall strain was an independent prognostic marker of mortality without the analysis of other parameters such as TAPSE and S’ of the tricuspid annulus.^63^In addition to RV strain, RV dilatation was a prognostic marker of patients with the severe form of the disease.^64^In conclusion, echocardiography plays a key role in this scenario, not only the comprehensive but also the problem-focused echocardiogram (point of care), as recently addressed by the position statement of the Department of Cardiovascular Imaging of the Brazilian Society of Cardiology (DIC/SBC).^65^

**Chart 8:** – Summary of echocardiographic changes in acute pulmonary embolism (PE)

PARAMETERS	ECHOCARDIOGRAPHIC CHANGE	IMAGE
Dimensions of the right ventricular cavity	2DE: basal linear diameter > 4.2 cm and longitudinal, > 8.1 cm. 3DE: end-diastolic volume > 87 mL/m2 in men and 74 mL/m2 in women; end-systolic volume > 44 mL/m2 in men and 36 mL/m2 in women. RV/LV dimension ratio > 1.0	
RV systolic function	Free-wall hypokinesia or akinesia. FAC < 35%. TAPSE < 17 mm. Tissue Doppler: S’ wave < 9.5 cm/s. Free-wall longitudinal strain above -20% (<20% in absolute values). 3DE: ejection fraction < 45%.	
Interventricular septum	Straightening or leftward shift due to RV pressure overload.	
Pulmonary trunk diameter	Dilatation (above 3.0 cm diameter).	
Tricuspid regurgitation	Increased tricuspid regurgitation due to increased pulmonary artery systolic pressure.	
“60/60 sign”	Pulmonary ejection acceleration time < 60 ms with mid-systolic notch + tricuspid valve peak systolic gradient < 60 mm Hg.	
“McConnell sign”	Hypokinesia or akinesia of basal and mid segments of the RV free wall and preserved apical contractility.	
Inferior vena cava	“Collapsibility index” <50% during inspiration.	
Intracavitary thrombus	Single pathognomonic finding (present in right cavities and/or pulmonary trunk or branch arteries in only 4% of cases).	

### 2.4. Recommendations

1. Bedside echocardiography is a mandatory test for all hemodynamically unstable patients with clinically suspected PE.^39 , 43 , 44^(grade of recommendation: I/level of evidence: C).
2. Echocardiography is not mandatory in the diagnostic routine for hemodynamically stable patients with suspected PE in diagnostic algorithms.^1 , 35 - 37^However, it represents an important tool for discriminating and defining differential diagnoses.^38 - 41^(grade of recommendation: IIa/level of evidence: B).

## 3. Intravascular Ultrasound in Deep Vein Thrombosis Diagnosis

### 3.1. Ultrasound Signs of Vein Thrombosis: B-mode and Doppler

If not properly diagnosed and treated, deep vein thrombosis (DVT) can lead to pulmonary embolism (PE), a potentially fatal condition.^66^Thrombus formation often occurs in regions of slow flow within the venous system, such as the cusp of a valve, spreading along the vein lumen, which is completely or partially filled by the thrombus. During the acute phase, the thrombi induce an inflammatory response on the adjacent venous wall. This is known as thrombophlebitis, and symptoms include cramp and local pain.^67^Many factors can trigger venous thrombosis. In the case of venous catheters, wall trauma induces the inflammatory response that leads to thrombosis within the vein lumen.^68 , 69^However, clinical signs of DVT can often be nonspecific; therefore, medical history, physical examination, and awareness of the main factors associated with the thrombotic process are essential.^16 , 70^

Risk factors for venous thrombosis can be grouped in clinical prediction models. The Wells score is a well-established method used to assess the clinical probability of a DVT diagnosis. Considering that only 50% of clinical DVT diagnoses may be correct, supplementary imaging is required to confirm or rule out diagnosis.^70^The evaluation of patients with suspected DVT usually involves stratification, clinical score, D-dimer measurement, and intravascular ultrasound (VUS).^6^

#### 3.1.1. Imaging Methodology – Technical Aspects

VUS is the method of choice for suspected DVT, providing information on venous anatomy and function. In addition, it is noninvasive, does not use nephrotoxic contrast, and is reproducible and inexpensive, although it is operator- and device-dependent.^71^Therefore, real-time B-mode ultrasound (US) images of the vein wall and lumen are obtained with transducer compression maneuvers and color flow Doppler (CFD), pulsed Doppler, or power Doppler.^72^

As recently stated by the Department of Cardiovascular Imaging,^73^the imaging technique to study DVT includes the following items:

#### a) Patient’s position:

For imaging of the lower extremities, the patient should be placed in a comfortable supine position with the torso and head elevated up to 30°, lying close to the edge of the bed, on the same side of the operator, with the hip slightly abducted and the knee slightly flexed. For the common and superficial femoral veins and the posterior tibial vein, the patient should keep the leg externally rotated and the knee slightly flexed. For the popliteal vein and peroneal veins, the patient should be placed in a prone or lateral decubitus position, resting on the contralateral limb with the knee slightly flexed. In case of any clinical condition, the patient may remain seated at the edge of the bed with the legs hanging down.^74^For the peroneal, muscular, and posterior tibial veins of the calf, the patient may sometimes be placed in the supine position, with the leg bent and the foot resting on the bed. Conversely, imaging study of iliac veins and the vena cava is conducted with the patient in the supine position. Prior preparation is required to reduce intestinal gases. In case of suspected post-thrombotic syndrome, if possible, the patient should also be examined in the upright position. For imaging of the upper extremities, the patient should be placed in the supine position, with the limb stretched alongside and slightly away from the body.

#### b) US equipment adjustment

Devices that produce high-quality images in B-mode and that are equipped with spectral Doppler and color Doppler should be used. The choice of transducer is primarily based on the relationship between the transducer’s frequency and the location of the object to be studied. High-frequency transducers produce high-quality images but have low tissue penetration. On the other hand, low-frequency transducers penetrate deeper, but produce lower quality images. Linear transducers with frequencies between 4 and 10 MHz are often used to investigate upper- or lower-extremity deep veins, whereas linear transducers with frequencies between 7 and 13 MHz are used to investigate upper- or lower-extremity superficial veins. For evaluation of swollen lower limbs and patients with obesity (ie, with greatly increased diameters), iliac veins, and the inferior vena cava, a multi-frequency convex transducer (between 1 and 5 MHz) may be necessary, as it has lower wave frequencies and deeper US wave penetration. Presets should be properly calibrated for imaging of the deep and superficial venous systems, with adjusted focal zone and gray scale, so that the lumen is dark in the absence of stasis or thrombosis. Spectral or color Doppler gain should be adjusted to a low velocity scale suitable for evaluating venous flow. For deep vein evaluation, VUS devices must be adjusted with proper focus and line density for two-dimensional imaging. The structures are evaluated in cross-sectional and longitudinal views.^73^In general, veins are easily compressible by the transducer.^71^

#### c) Imaging sequence:

**B-mode:** the transducer is initially positioned below the inguinal ligament, allowing visualization of the saphenofemoral junction. Longitudinal and cross-sectional scanning of the entire lower-extremity venous system is then performed. In the cross-sectional view, transducer compression must be applied especially along the common femoral, femoral, and popliteal veins with a distance of approximately 2 to 3 cm between each compression. The use ofVUS as a diagnostic technique for DVT was first described by Talbot in 1982, and the method has been improved over the years.^75^A normal vein without thrombosis shows complete lumen collapse during transducer compression of the walls ( Figures 7 and 8 ).

**Figure 7:** – A-E: Venous compressibility evaluation by the ultrasound transducer in the investigation of venous thrombosis. A: artery and vein without transducer compression. B: normal vein with maximum compression. C: dilated and noncompressible vein with acute/fresh thrombus. D: normal- to small-caliber vein, slightly compressible by transducer maneuvers and consistent with findings of chronic/old venous thrombosis. E: recurrent thrombosis.

**Figure 8:** – Cross-sectional view of the common femoral artery and vein for venous compressibility evaluation.

The figures show maximum common femoral vein compressibility in B-mode (top) and color flow Doppler (bottom). A = artery, V = vein.

**Doppler:** This technique is intended for recording venous flow velocities, which are low under normal pulsed Doppler conditions. In the presence of venous thrombosis, velocities may be further reduced, requiring scale adjustment. Lower-extremity venous flow is spontaneous and phasic in response to breathing and variations in intra-abdominal pressure ( Figure 9 ). During inspiration, velocities decrease due to increases in intra-abdominal pressure; conversely, distal compression maneuver (eg, calf compression) shows a flow peak in the patent interrogated vein. In some cases, proper flow detection is only possible by power Doppler, which can detect very low velocity flows despite not showing flow direction.

**Figure 9:** – Pulsed Doppler of the common femoral vein showing normal phasic flow in response to breathing movements, consistent with the absence of occlusive thrombosis.

#### d) Changes in B-mode, spectral Doppler, and color Doppler in the diagnosis of recent/acute DVT:

US diagnosis of recent/acute DVT is based on total or partial changes in the affected vein under transducer compression, whereas vein dilation is based on the presence of intraluminal thrombotic material. Changes in vein compressibility, even if a very recent episode of DVT has occurred, may be the most important finding for venous thrombosis diagnosis. The presence of a thrombus can only be ruled out if the vein lumen is obliterated after compression. Otherwise, the lumen may be partially filled with thrombi. During the compression procedure, the adjacent artery should be observed.

In most cases, recently thrombosed veins are distended and have larger diameters than the adjacent arteries. This distension is also important in the differentiation between acute and chronic thrombi. Occlusive thrombus is characterized by vein dilation and absence of detectable flow by pulsed Doppler, CFD, and power Doppler ( Figure 10 A and B ). Vein dilation has been described as an accurate parameter for identifying acute DVT.^76^An acute thrombus is composed primarily of a dense fibrin mesh that persists for approximately 5 to 7 days. During this phase, the thrombus is more echolucent or has intermediate echogenicity, appearing less bright than adjacent tissues. Diagnosing acute/fresh thrombus is important, given that anticoagulation and fibrinolytic therapies are more effective during this phase. Venous thrombosis is generally considered acute within the first 2 weeks of onset.^77^A fresh clot often forms at the end of an acute thrombus and floats freely inside the vein. Caution is needed during manipulation to prevent it from moving ( Figure 10 A-C ). Besides venous compressibility, venous wall appearance (DVT is often characterized by thin and smooth walls), vein lumen size, valve functionality, and the presence of collateral circulation should also be evaluated.

**Figura 10:** – Trombose oclusiva e não oclusiva de aspecto agudo ao ultrassom. A: corte transversal da veia femoral comum; B: veias fibulares evidenciando dilatação e incompressibilidade sem evidências de fluxo ao mapeamento de fluxo em cores e ao Power Doppler; C: corte longitudinal da veia femoral comum com e sem mapeamento de fluxo em cores evidenciando trombo flutuante de aspecto recente e não oclusivo.

Regarding flow, it may be reduced or absent depending on the degree of partial or complete obstruction of the lumen, with absence of respiratory variation. However, a partially obstructed lumen may not cause changes in Doppler’s flow signs.

#### e) Changes in B-mode and color Doppler in the diagnosis of clinically subacute and chronic/old DVT^11^:

In general, clinical evaluation of DVT considers the thrombus to be acute within the first 2 weeks of onset, subacute between 2 weeks and 6 months, and chronic after 6 months of onset.^72 , 78^Although the thrombus becomes more echogenic as DVT progresses, this change is variable and, most often, the age of the thrombus cannot be accurately estimated solely by echogenicity. For this reason, recently formed hypoechoic or anechoic thrombi are consistent with a diagnosis of acute thrombosis. Isoechoic or hyperechoic intraluminal images, however, make it more difficult to accurately estimate the age of the thrombus. During this period, retraction or thrombolysis may occur with a less distended vein; hence the importance of serial imaging. After retraction or thrombolysis, flow may not be necessarily restored to normal, and the vein walls become thick ( Figure 11 ).^71 , 72 , 79^Thus, VUS cannot always determine the age of a thrombus solely by its appearance or echogenicity.^71 , 72 , 79^

**Figure 11:** – Chronic post-thrombotic changes. A: B-mode image showing no dilation of the femoral vein. Echogenic luminal image consistent with chronic post-thrombotic changes; B: power Doppler image showing reduced luminal flow; C: B-mode image showing posterior tibial veins with thick walls; D: power Doppler image showing partial luminal narrowing of posterior tibial veins.

If the thrombus does not heal completely after acute thrombosis, it will become infiltrated with fibroblasts, organize, and reendothelialize. Fibrosis produces scarring, wall thickening, and synechiae that can cause partial vessel obstruction and persist for years. This residual material is no longer called thrombus. Thickening of the venous wall is a common finding, the caliber may be reduced and, depending on the venous caliber, the flow can be altered. In some cases, post-thrombotic fibrotic scars appear as the condition progresses. They are echogenic and sometimes have calcifications that produce acoustic shadows, causing plaque-like images along the venous wall. The Society of Radiologists in Ultrasound recommends that the term “chronic post-thrombotic changes” should be used to describe the changes that persist on US after acute DVT. Therefore, US abnormalities associated with DVT should be classified into “acute venous thrombosis, chronic post-thrombotic changes, or indeterminate changes.” The last one should be used when findings are uncertain and the age of the thrombus cannot be determined only by VUS, as with clinically subacute DVT. According to these recommendations, exceptionally, some cases may be classified as subacute thrombosis on the US report if:

1. There is a previous US showing acute DVT;
2. There is a new study showing that the appearance of the thrombus has changed.^80^

These classifications are supported by a recent SBACV publication.^81^However, in an effort to prevent the term “indeterminate changes” from creating confusion as to whether clinicians should start treatment or not, the publication recommends that the vascular sonographer should report image features in detail, analyze the clinical information regarding time of occurrence, and preferably refer to these changes as “predominance of acute or recent changes” or “predominance of chronic or old changes.” Thus, the term “indeterminate changes” would only be used when the time of the event cannot be determined.

DVT can affect several venous segments alone or together, as in plantar vein thrombosis, an uncommon diagnosis. It should be suspected in the presence of acute pain and/or edema of the foot. Recently observed US features of plantar DVT are venous dilation and luminal thrombus with reduced vein compressibility ( Figure 12 ).

**Figure 12:** – Color flow Doppler image showing venous thrombosis of noncompressible plantar veins without flow.

US features that may be present in acute/recent and chronic/old DVT are shown in Chart 9 , adapted from Gornik HL and Sharma AM.^72^In some cases, there is overlap of features, which should be reported as of “indeterminate age.”

**Chart 9:** – Ultrasound features of deep vein thrombosis

Feature	Acute/recent	Predominance of acute thrombus or chronic thrombus	Chronic/old post-thrombotic changes
Thrombus echogenicity	Hypoechoic or isoechoic	Variable (more echoic than acute DVT)	Hypoechoic
Presence of mobile thrombus	May be present	Generally absent	Absent
Attachment of thrombus to vein wall	Loosely attached	Firmly attached	Hyperechoic fibrous tissue attached to the vein wall or lumen
Venous wall appearance	Variable	Variable	Venous wall thickening and scarring Calcium deposits may be seen
Vein lumen	Dilated	Retracting to normal size	Smaller than normal size
Compressibility	Slightly compressible or deformable	More compressible than acute DVT	Partially compressible
Compressibility	Generally absent	May be present	May be present
Venous valve function	Generally competent	May be incompetent	Generally incompetent

Impaired respiratory phasicity and reduced flow velocity in a venous vessel of normal appearance, with a present and compressible flow, may be associated with the thrombotic involvement of a venous segment proximal to the vessel being evaluated ( Figure 13 ).

**Figure 13:** – Left external iliac vein thrombosis: flow comparison between the left and right common femoral veins. A: color Doppler image showing no flow in the dilated left external iliac vein, with lumen filled with a thrombus of recent appearance; B: spectral Doppler image showing continuous flow in the common left femoral vein secondary to thrombosis of the ipsilateral external iliac vein; C: normal phasic flow in the contralateral common femoral vein.

## 3.2. IVUS Protocols in DVT

IVUS has been used since the 1960s for the diagnosis of venous thrombosis, and the method has been improved over the years by the results of imaging studies of venous blood flow measurement and venous compressibility. As previously stated, venous compressibility evaluation combined with color and spectral Doppler of flow is the recommended approach for the detection of venous clots. This is partly due to availability, ease of implementation in a variety of clinical settings, and accuracy.

IVUS has been the reference method for diagnosis since the first accuracy studies of symptomatic and asymptomatic patients with suspected above- and below-knee DVT.^82^Lower extremity venous compression US has largely replaced venography in DVT diagnosis, as it has a sensitivity above 90% and a specificity of approximately 95% for the diagnosis of proximal thrombosis. For distal thrombosis, the specificity is the same (above 90%), but the sensitivity is reduced to approximately 65%. Conversely, duplex US (a combination of B-mode and Doppler of all veins) is the first-line imaging test for DVT diagnosis, with a sensitivity of 96% and a specificity of 98% to 100%. However, duplex US is time-consuming, requires patient transport to a diagnostic imaging center, and the immediate availability of an imaging specialist.^38 , 83 , 84^In addition, to obtain an accurate diagnosis, the operator must be familiar with the method’s indications and limitations. Only 20% to 30% of imaging tests of suspected DVT show abnormal findings, and 90% of patients with fatal PE are asymptomatic for DVT.^16 , 18^Test limitations and, therefore, accuracy are associated with obesity, mobility restrictions, major edema, and/or presence of wounds, orthopedic devices, bandages/plasters, etc. Relevant clinical information and focused physical examination are essential to assist in the interpretation of IVUS results during DVT investigation, and adequate protocol training is imperative. Despite these limitations, the easy handling, harmless nature, and wide availability of portable devices make this tool extremely applicable in daily practice. In addition, advances in electronic miniaturization have resulted in the creation of pocket-sized US scanning devices equipped with linear array transducers, further disseminating the use of compression VUS for DVT diagnosis.^85^

Regardless of the test protocol of choice, the examination room should be comfortably warm to prevent venospasm. To evaluate the inferior vena cava (IVC), the patient should be placed in a reverse Trendelenburg position.^71^The Society for Vascular Ultrasound recommends an examination time of approximately 75 minutes for a complete bilateral examination: 40 to 60 minutes for the actual imaging test and 15 minutes for clinical data analysis, consultation of previous imaging tests, and patient and room preparation.^86^

## 3.3. Test Protocols

With increased availability of VUS devices, which usually have excellent resolution, the training of nonexperts has become very frequent and gained increased acceptance, especially in the emergency and urgent care departments and in distant locations. Thus, imaging tests for suspected DVT performed by critical care and emergency physicians and last-year radiology/US/VUS residents is a current reality. The accuracy of emergency physician-performed US was assessed in a systematic review and meta-analysis of 16 studies with 2,379 patients. The prevalence of DVT was 23% (7.4%-47.3%), with a mean sensitivity of 96.1% (95%CI: 90.6%-98.5%) and a specificity of 96.8% (95%CI: 94.6%-98.1%). However, the studies included were very heterogeneous, the training process of emergency physicians was poorly described, and most of the studies had short follow-up periods. These data were not reproduced in a prospective study published in same year with a much smaller sample size, indicating the need for strict training protocols for these professionals, given that only the first three tests performed by nonexperts were supervised.^84 , 87^Many of the protocols performed by nonexperts have been developed for settings where experts are not available or to reduce the number of VUS performed after normal laboratory working hours. Thus, a single simple imaging protocol is not enough to definitively rule out DVT. In addition to proper supervised training, pretest DVT probability analysis is essential, that is, the use of clinical PE/DVT probability scores and awareness of the lower accuracy of VUS in the diagnosis of asymptomatic below-knee DVT. Studies on the topic did not report any major disadvantage for patients, and well-structured protocols with Wells’ clinical probability criteria helped improve test accuracy and proper referral of patients without easy access to imaging experts.^82 , 88 - 90^

During VUS investigation of venous thrombosis, the parameters previously described should be analyzed, ie, the combination of grayscale components (B-mode) with compression transducer maneuvers and CFD and spectral Doppler evaluation. Imaging test protocols use the following parameters to reach diagnosis: vein compressibility (the most important), vein caliber (diameter), US features of vein wall and lumen, and CFD and spectral Doppler evaluation.

There are some well-established complete and focused (point-of-care) DVT imaging protocols. They are chosen according to device availability at the facility where the patient is being examined, time of patient arrival, and whether there is an expert available to perform VUS.

DVT imaging protocols ( Figures 14 and 15 ):

**Figure 14:** – Whole-leg vascular ultrasound protocol for deep vein thrombosis diagnosis. A: schematic representation of compression points throughout the above-knee venous system and color flow Doppler and spectral Doppler of the femoral segment; B: schematic representation of compression and color flow Doppler and pulsed Doppler of the popliteal segment; C: schematic representation of compression and color flow Doppler of the distal segment.

**Figure 15:** – A: complete lower extremity venous compression ultrasound protocol; B: three-point extended compression ultrasound protocol; C: two-point extended compression ultrasound protocol.

1. Whole-leg VUS – Figure 14A-C .
2. Complete lower extremity venous compression US – Figure 15A .
3. Three-point extended compression US – Figure 15B .
4. Two-point extended compression US – Figure 15C .

### 3.3.1. Complete DuplexVUS

The Society of Radiologists in Ultrasound and the Brazilian Society of Angiology and Vascular Surgery currently recommend (grade of recommendation: I; level of evidence: A) complete duplex US for the diagnosis of acute DVT. In addition to being a more complete patient approach, examining the infrapopliteal segment allows the diagnosis of other musculoskeletal diseases that are part of the differential diagnosis of DVT, such as Baker’s cyst, hematomas, and muscle diseases.^80 , 81^The complete protocol involves using all US resources to diagnose thrombosis: evaluation of compressibility, caliber, vein wall and lumen appearance on US, and color and spectral Doppler of all veins from below the inguinal ligament to ankle in all segments (femoral, popliteal, posterior tibial, and peroneal veins) ( Figure 14A-C ). In general, the investigation of venous thrombosis in the anterior tibial veins is not included in this protocol due to the rarity of the condition. It is only investigated when there are signs/symptoms. Evaluation of the superficial and muscular venous systems should be conducted especially in symptomatic regions.

Duplex ultrasound includes compression at 2-cm intervals, CFD analysis of vessel filling (using necessary scale adjustments), observation of flow phasicity, and spectral Doppler analysis of femoral and popliteal veins morphology. If complete testing is performed only in the symptomatic limb, spectral Doppler evaluation should be performed in both common femoral veins to assess symmetry. In case of asymmetric flow pattern, the intra-abdominal veins should be investigated (grade of recommendation: I; level of evidence: A).^91 - 94^

### 3.3.2. Complete Lower Extremity Venous Compression US

In 1982, Steve Talbot described the compression technique that would become the standard diagnostic method for DVT.^75^This protocol involves only the evaluation of compressibility in all veins below the inguinal segment to the ankle in all segments ( Figure 15A ). As previously stated, loss of vein compressibility is the most reliable indicator of thrombus.

### 3.3.3. Extended Compression US (Three-point)

This protocol involves only the evaluation of compressibility from the common femoral vein to the popliteal vein, where all leg veins converge. It is also called the three-point protocol, as it assesses the compressibility of all proximal veins of the investigated lower extremity ( Figure 15B ). The sensitivity found in this protocol was significantly higher than that in the two-point compression protocol (90.57% vs. 82.76%), although specificity was the same (98.52%).^95^A diagnostic accuracy study of the three-point protocol performed by emergency physicians found very similar results (91.7% accuracy; 95% CI, 85%-95.6%).^96^A recent meta-analysis of 17 studies from 16 original articles compared three-point vs. two-point point-of-care US (POCUS) protocols. Overall, the two-point POCUS had similar pooled sensitivity (0.91; 95% CI, 0.68-0.98; P = 0.86) and specificity (0.98; 95% CI, 0.96-0.99; P = 0,60) to the three-point POCUS (sensitivity 0.90; 95% CI, 0.83-0.95 and specificity 0.95; 95% CI, 0.83-0.99). The false-negative rates of two-point (4.0%) and three-point POCUS (4.1%) were almost similar. Meta-regression analysis showed that high sensitivity and specificity tended to be associated with the initial POCUS performer and separate POCUS training for DVT^97^(grade of recommendation: II; level of evidence: A).

### 3.3.4. Two-point Compression US

In this protocol, compressibility is evaluated at only two sites: the common femoral vein, 1-2 cm above and below the saphenofemoral junction (groin); and the popliteal vein, up to where the leg veins converge ( Figure 15C ).

In general, emergency physicians are able to perform this protocol after training, with good reproducibility. There are several methods for performing this imaging protocol in the emergency setting. Most training programs include theoretical lectures on imaging technique, mock training, and observation of a number of different tests. However, some studies mention training programs that last only 10 minutes or less than 2 hours. This significant difference between protocols hinders comparisons between studies and, consequently, affects accuracy.^95 - 100^Emergency physicians can achieve a level of competence equivalent to that of experts, but substantial training and practice are required to achieve and maintain this performance. To reach this level of excellence, they need regular training in US imaging.

A study comparing complete two-point procedures performed by experienced emergency physicians showed that 362 of 2,451 patients had DVT. Two-point US would not have been able to diagnose 23 patients with proximal DVT (6.2%).^100^A randomized controlled trial with more than 2,000 patients comparing whole-leg vs. serial two-point US plus D-dimer measurement showed that the two strategies are equivalent for the management of symptomatic outpatients with DVT. However, in the latter, the physicians were VUS experts, which indicates that this testing protocol should be performed by well-trained physicians and follow clinical probability scores to be accurate. In most cases, if symptoms persist or if D-dimer is elevated, compression US must be repeated.^101^

Finally, the recommended complete imaging protocol is the one intended for DVT investigation because it scans the entire deep vein leg system, and it should be performed by experts. In addition, it requires dedicated high-grade devices and patient transport from the emergency department/ward to the imaging center.

Conversely, two- and three-point compression US protocols are simple, fast, do not require experts or sophisticated devices, and can be performed at the bedside. These protocols are suitable for emergency departments and hospital wards, especially after working hours and on weekends. Together with pretest probability evaluation, they should be able to provide an initial assessment of patients with suspected DVT.

Two-point compression US protocols are an extremely important tool for critically ill patients in certain settings, such as the current coronavirus disease 2019 (COVID-19) pandemic. There is a relationship between COVID-19 and a prothrombotic state, which favors the occurrence of thromboembolic phenomena. Thus, in case of a clinical possibility of DVT, performing a quick, focused US of the suspected limb should be considered when the diagnosis has an impact on patient management.^102^

## 3.4. Differential Diagnosis

The symptoms triggered by a thrombotic event are common to several other acute or chronic conditions, such as ruptured synovial cysts, chronic venous insufficiency, hematomas, and muscle tears.^103^In other cases, venous thrombosis may occur due to extrinsic compression by vascular abnormalities, such as aneurysms and pseudoaneurysms, or extravascular conditions, such as hematomas, abscesses, synovial cysts, lymphadenomegaly, and neural and hematological tumors.^104^Complete US imaging of the limb provides the ability to identify associated clinical conditions and differentiate them from nonthrombotic abnormalities. Conversely, a swollen limb associated with stasis or decreased respiratory variability in the femoral vein may indicate extrinsic compression in the pelvic region. Lymphadenomegaly, uterine fibroids, retroperitoneal tumors, and May-Thurner syndrome are among possible causes.^105^In this case, the sonographer should draw attention to these findings and suggest them as possible causes on the report.

Lymphadenomegaly, especially in the inguinal region due to lymphoproliferative disease, may lead to clinical manifestations of marked limb edema due to a possible compressive venous component or insufficient regional lymphatic drainage. Although easy to identify, occasionally the tumor mass can be so large that it hides the vein, making it difficult to characterize the flow or even the presence or absence of thrombus.^106^

Pseudoaneurysms, despite having well-defined color VUS features, can develop variable echogenicity in B-mode when thrombosed, depending on the volume and time of occlusion. When massive, they can also lead to venous compression and be associated with other conditions, such as blunt trauma or bone tumors, hindering vein identification.^107^

A common differential diagnosis of DVT on US is muscle hematoma, especially when located in the calf muscles. Clinical presentation is very similar to venous occlusion and is not always clear. Trauma or sudden pain are often mentioned in medical history. Su et al.^108^have described features suggestive of hematoma: mixed echogenicity, absence of anechoic regions, perilesional hyperechogenicity (especially enhancement in the underlying tissue), and a relationship between the longitudinal and transverse axis of lesions > 2.

One of the most common additional nonvascular findings on venous imaging is the popliteal synovial cyst, or Baker’s cyst. It is most commonly located in the posteromedial surface of the popliteal fossa and most often does not trigger symptoms in the vascular system. However, in rare conditions, an intact cyst can present posterolaterally, with varying degrees of venous, arterial, and nervous compression, thus triggering neurovascular symptoms in the affected limb. In case of rupture towards the calf, Baker’s cyst may mimic the main features of acute venous thrombosis. Despite clinical similarity, differentiation between articular and vascular cysts must be made because each condition requires a completely different treatment approach. Some cyst features may help differentiate it from thrombotic events. Baker’s cyst communicates with the joint cavity close to the medial head of the gastrocnemius muscle, which is a key diagnostic feature ( Figure 16 ). Despite its liquid content (therefore anechoic), it may have clots inside. Baker’s cysts are usually single and multilocular.^109^

**Figure 16:** – Baker’s cyst in the posteromedial region of the knee (differential diagnosis of deep vein thrombosis).

Soft tissue tumors, benign tumors (schwannomas, fibrous histiocytoma, neuromas), or malignancies (sarcomas, osteosarcoma) may mimic the clinical features of venous thrombosis or present as a compressive condition ( Figure 17 ).^110^

**Figure 17:** – Venous hemangioendothelioma. A: clinical appearance; B: hypoechoic image surrounding a superficial vein on color flow Doppler; C: histopathologic examination.

VUS is a very accessible and widely available tool for the initial investigation of venous thrombosis; however, nonthrombotic conditions should be considered in the differential diagnosis. In experienced hands, IVUS allows alternative diagnoses of conditions that often occur in cases of suspected venous obstruction.

## 3.5. Recommendations

1. Complete duplex US should be used for the diagnosis of acute DVT (grade of recommendation: I; level of evidence: A).
2. Spectral Doppler of both common femoral veins should be performed to evaluate symmetry. In case of asymmetric flow pattern, the intra-abdominal veins should be investigated^91 - 94^(grade of recommendation: I; level of evidence: A).
3. Based on the consensus, the following terminology should be used to describe US abnormalities associated with DVT:
  1. Acute/recent;
  2. Chronic/old;
  3. Predominance of acute changes;
  4. Predominance of chronic changes.

The term “indeterminate” might be used only in the few occasions that the time of the event cannot be determined.

## 3.6. How to Write Imaging Test Reports

**Findings:** first, it is fundamental to describe technical quality, the conditions in which the test was performed (if urgent or not), whether the patient was able to stay in the proper position, and if any major edema or other factors that could prevent proper technical quality were detected.

Vein evaluation should be described separately, especially if there is venous thrombosis. All imaging features must be described: B-mode and Doppler (which can lead to the conclusion on the presence or absence of thrombosis), time of clinical onset, and whether the thrombus is acute, indeterminate, or has chronic post-thrombotic changes.

1. Vein features to be included in the findings:
2. Vein caliber (compare with the contralateral vein and/or with the diameter of the adjacent artery) and wall appearance in all evaluated segments;
3. Total or partial compressibility and specification of the affected segment;
4. Presence or absence of intraluminal material. If present, evaluate echogenicity, complete or partial luminal obstruction, and presence or absence of a mobile component;
5. Presence or absence of collaterals;
  - Regarding flow:
  - Color Doppler: describe if present or absent and if it causes partial or complete luminal obstruction;
  - Spectral imaging: flow may be absent. If present, describe whether the flow is continuous or phasic in response to breathing and whether it increases (or not) distal compression and/or associated reflux.
6. If applicable, include possible differential diagnoses: Baker’s cyst, tumors, signs suggestive of lymphedema, and images suggestive of hematoma.

**Conclusions:** Report if there are signs of thrombosis or not. If so, specify whether it is totally or partially occlusive and whether the vein(s) and venous segments are affected. Specify whether it is acute/recent or there are chronic/old post-thrombotic changes. In cases of clinically subacute thrombosis (between 2 weeks and 6 months), the following terminology should be used: predominant signs of acute thrombosis or predominant signs of chronic thrombosis. In cases of nonocclusive thrombosis, specify whether there is associated flow or not. Features that should be reported to support the conclusions are summarized in Chart 10 .

**Chart 10:** – Ultrasound features to be reported that support the conclusions

ULTRASOUND SIGNS	TIME OF CLINICAL ONSET	DEGREE OF COMPRESSIBILITY	THROMBUS ECHOGENICITY	VEIN DIAMETER	TYPE OF FLOW
Acute/recent thrombosis	Less than 15 days	Total or partial noncompressibility	Hypoechoic or echolucent	Increased	Absent/continuous or reduced phasicity
Chronic/old thrombosis	More than 6 months	Total or partial noncompressibility	Hyperechoic/residual fibrotic trabeculations	Reduced or normal	Continuous/absent/with residual changes
Predominant signs of acute thrombosis, predominant signs of chronic thrombosis or, as an exception, indeterminate	Clinically subacute: Between 2 weeks and 6 months	Total or partial noncompressibility	Isoechoic/mixed echogenicity/mostly hypoechoic/mostly hyperechoic	Normal or slightly changed	Reduced/continuous/absent phasicity

## 4. Vascular ultrasound in the diagnosis of recurrent deep vein thrombosis and post-thrombotic syndrome

### 4.1. Recurrent venous thrombosis

This refers to a new episode of venous thromboembolism (VTE) in an individual with a previous history of this type of event. In cases of lower-extremity thrombosis, recurrence may manifest as a thrombus in a new location (propagation into a segment proximal or distal to the initial event, different segment of the same extremity, different extremity) or in a previously affected segment.^111^In current medical practice, the recommendation is that a patient diagnosed with a first episode of DVT be treated with an anticoagulant for 3 to 6 months,^112^which most often results in resolution of symptoms due to recanalization of the vein or emergence of collateral circulation.

Recurrent thrombosis is a common event. After anticoagulant discontinuation, the recurrence rate is estimated to progressively increase over time, reaching 40% among all patients within 10 years (cumulative incidence: 7.2% within 6 months, 11% within 1 year, 19.6% within 3 years, 29.1% within 5 years, 34.3% within 8 years, and 39.9% within 10 years).^113^

The most important risk factor for recurrence is the cause of the first episode of DVT. In patients with DVT caused by a transient risk factor (eg, after major surgery), the annual risk is only 1%, and a 3-month treatment may be considered depending on other risk factors.^114^When there is no identifiable main causal factor, or when there is only a transient minimal cause (“unprovoked”/idiopathic thrombosis), which occurs in approximately half of DVT cases, the prevalence of recurrence increases to 5%-10% at 1 year and up to 30% at 5 years,^115^which warrants treatment continuation with vitamin K antagonists (VKAs) or non-vitamin K oral anticoagulants (NOACS) for 6 months or over, as these significantly reduce the risk of recurrence by 80%-90%.^116^

Recurrence after stopping anticoagulation primarily depends on two factors: first, whether acute DVT has been effectively treated, including minimum duration of anticoagulant treatment; and second, the presence of intrinsic risk factors that increase recurrence, notably malignant disease and thrombophilias.^117^Optimal duration of anticoagulant treatment remains under discussion and is based on the extent of thrombosis (proximal or distal veins) and on the cause of the thrombotic event, when related to a main predisposing factor. We follow the recommendations of the American College of Chest Physicians (ACCP).^112^

In addition to the precipitating cause of DVT, other factors should be considered to determine the risk of recurrence. The sonographer should be attentive to clinical factors that are associated with recurrent thrombosis, such as: unprovoked DVT (RR, 2.3; 95% CI, 1.8-2.9), obesity (RR, 1.6; 95% CI, 1.1-2.4), male sex (RR, 2.8; 95% CI, 1.4-5.7), positive D-dimer test (RR, 2.6; 95% CI, 1.9-3.5), residual thrombosis (RR, 1.5; 95% CI, 1.1-2.0), hereditary thrombophilia (RR, 1.5; 95% CI, 1.1-1.9), inflammatory bowel disease (RR, 2.5; 95% CI, 1.4-4.2), antiphospholipid antibody (RR, 2.4; 95% CI, 1.3-4.1), and malignancy.^117^

Malignant disease is a major risk factor for both thrombosis and recurrent DVT. The risk for recurrent DVT is 2- to 4-fold higher in patients with cancer, reaching 4.2-fold in those undergoing chemotherapy.^118^This treatment affects the vascular endothelium and the coagulation cascade, thereby releasing prothrombotic substances during tumor lysis.

Knowing the natural history of DVT is important to choose the appropriate therapy and, in the case of vascular ultrasound, to conduct patient follow-up. Recanalization is a dynamic process that depends on lytic or procoagulant forces; it starts in the first week after an episode of DVT and continues for months, with most of the thrombotic mass being reduced in the first 3 months.^119^Complete resolution has been demonstrated in 56% of patients undergoing ultrasound scanning during follow-up,^120^and this rate is different for proximal and distal thromboses. In distal thrombosis, complete recanalization is more frequent and there are no sequelae such as significant reflux or obstruction. Conversely, the prevalence of reflux or obstruction in proximal thrombosis or thrombosis involving the two segments is much more significant.^121^

The recanalization rate is related to the prevalence of reflux in the venous system. The faster the recanalization, the greater the association with competent valves.^120 , 122^Reflux, associated or not with venous obstruction due to incomplete recanalization, may cause persistent venous hypertension, leading to the appearance of signs of chronic venous hypertension. These are known as post-thrombotic syndrome (PTS).

Although several studies have highlighted the importance of residual venous thrombosis, which is usually diagnosed by VUS examinations in the follow-up of patients with DVT,^123 - 125^a recent meta-analysis of 14 studies showed only a mild increase in the risk of recurrence (HR, 1.5; 95% CI, 1.1-2.0) in patients with residual venous thrombosis.^126^

### 4.2. Ultrasonic Diagnosis of Recurrent Thrombosis

There are no isolated VUS parameters that characterize recurrent thrombosis, or validated clinical models that allow its diagnosis. The diagnosis is based on reappearance of symptoms and clinical findings of DVT, considering the probability associated with risk factors and the presence of ultrasound findings suggestive of recurrent thrombosis.

Comparative findings of an examination of currently suspected recurrence versus a baseline examination at the end of treatment are the only validated method for the diagnosis of recurrent DVT. Therefore, the existence of previous examinations that clearly and accurately describe the extent of involvement and the degree of recanalization of the previous thrombosis is key.

Ultrasound findings are similar to those described for the diagnosis of the first thrombosis, and the presence of an old thrombotic mass may hinder the diagnosis of recurrence. Such findings are listed in Chart 9 of this document.

The diagnosis may be easier when recurrent thrombosis occurs in the contralateral extremity or in a new segment that was clearly not involved in the previous venous thrombosis. When it occurs in a previously involved segment, an increase in vein diameter by 2 mm under compression of the previously thrombosed segment has been reported as a criterion for the diagnosis of recurrent DVT.^127 , 128^Because an increase of 2 mm at maximum compression has a low positive predictive value, other authors have proposed the use of an increase in vein diameter ≥4 mm for the diagnosis of recurrent DVT.^129^A recent study that reviewed 36 published articles demonstrated that a new noncompressible vein or an increase in the diameter of a previously thrombosed vein segment ≥4 mm is sufficient to confirm the diagnosis of recurrent DVT. An increase in vein diameter of less than 2 mm allows recurrence to be ruled out, and an increase between 2 and 4 mm is considered unclear.^130^

Thrombus echogenicity, even when correlated with in vitro thrombus organization, makes the in vivo evaluation subjective. Importantly, even acute thrombi may have different stages of organization.^131^

La Gal et al.^132^demonstrated that a diagnostic strategy comparing an examination of currently suspected recurrence with a baseline examination at the end of treatment can be safely used to rule out recurrent DVT.

Such considerations reinforce the need for a thorough examination at the end of treatment to be used for comparison with subsequent procedures. All findings should be described, including residual diameter after maximum compression and site of measurement (it should be performed at the site of greatest residual thrombotic mass).

### 4.3. Post-thrombotic Syndrome

Post-thrombotic syndrome (PTS) is a chronic condition characterized by signs and symptoms that develop as a consequence of previous DVT. The most common complication appears in the long term and affects 20%-40% of cases of lower-extremity DVT, even in the presence of adequate anticoagulation.^133^

PTS has a significant impact on the quality of life of patients, compromising and limiting their daily activities and productivity. This impact can be compared to that of cancer and heart disease, in addition to causing a significant cost increase to the health care system. Data from Swedish studies showed costs about 75% higher than those of primary DVT.^133^

#### 4.3.1. Pathophysiology

The pathophysiology of PTS is a combination of obstructive phenomena and venous reflux due to valve insufficiency, which result in venous hypertension. Increased venous pressure in subcutaneous capillaries and microcirculation culminates in valve incompetence in perforating veins. In most cases, the obstructive process is characterized by recanalization occurring within 6-12 months of the acute event, which leads to a combination of partial obstruction and varying degrees of reflux in the affected segments. All changes ultimately result in destruction of venous valves, development of collateral valves in sites where a greater degree of obstruction persists, and a greater tendency to recurrence of acute episodes.^133 , 134^

A system of competent saphenous veins may act favorably in venous drainage when there is a significant obstructive component. Conversely, when there is insufficiency of preexisting saphenous veins, venous return will be even more compromised, and the clinical status of these patients may deteriorate more quickly.

The inflammatory process is the main factor in PTS, being present in the resolution of thrombosis. Fibrinolysis, thrombus organization, and neovascularization involve interleukin-6 and intercellular adhesion molecule-1, which causes valve damage within the first months of the acute phase of DVT. Prandoni and Kahn suggest that the absence of recanalization in this period is an important predictor of PTS.^133 , 135 , 136^

#### 4.3.2. Clinical Manifestations

PTS includes a wide spectrum of manifestations ranging from mild clinical signs to severe symptoms such as chronic pain in the affected limb (limiting daily activities), intractable edema, and ulcers. Other signs may appear, such as hyperemia, hyperpigmentation, venous dilations, and lipodermatosclerosis, in addition to symptoms such as pain, heaviness, paresthesia, pruritus, and cramps. In more advanced cases, cutaneous, subcutaneous, and lymphatic infections may occur.

The degree of residual luminal obstruction is directly related to the severity of repercussion of these clinical manifestations, leading to severe pain and venous claudication.

The following risk factors may contribute to the development of PTS:

- Obesity;
- Varicose veins;
- Proximal thrombosis and recurrence;
- Inadequate use of oral anticoagulant;
- Time: the slower the resolution of DVT, the greater the probability of PTS;
- Age and gender (there are contradictory studies);
- Other potential but not yet proven factors: malignancy, immobilization, surgery, pregnancy, and thrombophilia.

#### 4.3.3. Diagnosis

This condition should be diagnosed after the end of treatment of the DVT episode, ie, PTS cannot be characterized before the first three months.^133^

The diagnostic strategy for PTS should consist of clinical examination, laboratory tests, imaging studies, and air plethysmography. There is no reference laboratory, imaging, or functional test for the diagnosis of PTS. However, the presence of venous obstruction and reflux must be recorded by an imaging test, and an attempt should be made to inform which of the two pathophysiological components predominates, even if nonquantitatively.

The clinical, etiological, anatomical, and pathological (CEAP) classification and the venous clinical severity score (VCSS) used for chronic venous insufficiency (CVI) can be appropriately applied to quantify PTS with different measures.^133 , 137 - 139^

The Brandjes scale, the Ginsberg measure, and the Villalta scale were specifically developed to assist in the diagnosis of PTS, but unfortunately they have not been adopted comparatively in other studies. At the 2008 meeting of the International Society on Thrombosis and Haemostasis, held in Vienna, the Villalta scale was recommended for defining the presence of PTS, with a score >5 indicating the diagnosis. The presence of ulcer makes the score even more severe ( Chart 11 ).^140 , 141^

**Chart 11:** – Villalta scale for post-thrombotic syndrome 141

SYMPTOMS	NONE	MILD	MODERATE	SEVERE
Pain	0	1	2	3
Cramps	0	1	2	3
Heaviness	0	1	2	3
Paresthesia	0	1	2	3
Pruritus	0	1	2	3
Signs	0	1	2	3
Pretibial hematoma	0	1	2	3
Skin induration	0	1	2	3
Hyperpigmentation	0	1	2	3
Redness	0	1	2	3
Venous ectasia	0	1	2	3
Pain on calf compresssion	0	1	2	3
Venous ulcer	0	1	2	3

VUS is the imaging method of choice because of its low cost, availability in small centers, and high accuracy. In patients with a clinical presentation suggestive of PTS but no history of DVT, ultrasound is indicated to screen for evidence of previous DVT.

VUS provides the location, the measurement of vein diameters after the compression maneuver ( Figure 18 ), and, more subjectively, the degree of luminal obstruction in the affected veins, valve damage, and reflux characterization ( Figures 19 and 20 ). Although rarely used and infrequently available, air plethysmography is effective in quantifying venous reflux and is useful for the control of recanalization and diagnosis of rethrombosis.

**Figure 18:** – Two-dimensional ultrasound image with measurement of popliteal vein diameter after a compression maneuver.

**Figure 19:** – A: two-dimensional longitudinal image of the common femoral vein with parietal thickening and trabeculations/fibrous tissue inside the vessel. B: color flow Doppler showing flow around the trabeculation. C: similar findings in the popliteal vein.

**Figure 20:** – Reflux in the popliteal vein on color flow Doppler (red) and pulsed Doppler.

In addition to defining the diagnosis,VUS can be used for therapeutic planning. The test is important in the evaluation of the involvement of the iliocaval segment for endovascular approach and restoration of patency as well as in post-treatment follow-up, having a key role in the diagnosis of recurrent thrombosis.^137 , 142^

When there are barriers to the use of VUS, such as the presence of severe edema and lipodermatosclerosis, which often includes focal fibrocalcific nodules, some strategies should be adopted for improving image acquisition.

Modification of transducer position and angle of insonation of the ultrasound beam, reduction in transducer frequency, and use of trapezoid mode with linear transducers are recommended; if necessary, the transducer should be replaced with lower-frequency devices that enable greater penetration, such as convex, sector, and endocavitary transducers.

Patients with PTS should be examined in the supine and upright positions, which allows a better visualization of luminal changes, such as thickening and trabeculae, and promotes a more effective investigation of reflux ( Figures 19A-C and 20 ). Below-knee veins can also be evaluated with the patient seated, feet resting lightly on a platform, and muscles more relaxed, which facilitates the insonation of deeper veins.

In the iliocaval segment, not only should the physician assess patency but also look for signs of extrinsic compression, especially compression of the left common iliac vein between the direct common iliac artery and the adjacent vertebral body, usually L5.

The main ultrasound findings are reduced vein diameters and chronic luminal changes, such as parietal thickening and hyperechoic trabeculae, in addition to valve insufficiency. A less frequent finding is the presence of flow with an arteriovenous fistula pattern in focal points of venous segments affected by thrombosis, without repercussions in adjacent axial veins and arteries and of unknown clinical significance.^142^

## 5. Follow-up Protocols with Intravascular Ultrasound after Deep Vein Thrombosis

### 5.1. Introduction

DVT is a dynamic process with periods of recanalization, progression, and recurrence, and VUS is an important tool for the diagnosis and follow-up of DVT. Ultrasound findings play an important role in the outcome of this disease and are useful at different stages, which will be described below.

### 5.2. Recanalization

Recanalization of venous thrombosis is complex and involves mechanisms of intrinsic and extrinsic fibrinolysis, peripheral fragmentation, neovascularization, and thrombus retraction.^134 , 143 , 144^Such data have been confirmed by studies using VUS, which demonstrated thrombus regression within the first three months and complete recanalization in approximately half of patients within 6 to 9 months of the episode of DVT.^8 , 145^Partial recanalization and persistently occluded segments occur in approximately 20% and 5% of cases, respectively^146^( Figure 21A ).

**Figure 21:** – Process of neovascularization in recanalized thrombosis. A: cross-sectional view of a vein with echogenic images corresponding to the thrombus inside the vessel. Color Doppler demonstrates the presence of flow within the thrombus. B: Pulsed wave Doppler revealing the presence of flow within the thrombus.

Images of small tortuous vessels inside the thrombus and adjacent to the venous wall, detected on color flow Doppler and pulsed wave Doppler, suggest the presence of neovascularization in the complex process of recanalization^134 , 143^( Figure 21B ).

### 5.3. Recurrent Thrombosis

Although recanalization is an important step in the process, recurrent thrombotic events are not uncommon. The recurrence rate of symptomatic thromboembolism ranges from 5% to 13%, depending on the duration of patient follow-up with VUS. However, VUS-detected thrombotic events without clinical manifestations tend to be greater.^8^

The diagnosis of recurrent thrombosis is highly dependent on the ultrasound examination performed during the first episode. The following criteria are suggestive of recurrence:

1. Identification of new sites of thrombosis;
2. Occlusion of partially recanalized venous segments recorded in a previous examination;
3. An increase greater than 4 mm in the diameter of partially recanalized venous segments recorded in previous examinations^111 , 130^( Figure 22 ).
  **Figure 22:** – Ultrasound changes that may be found in the follow-up of patients with deep vein thrombosis. A: longitudinal view of the femoral artery (red) and femoral vein (blue). Presence of partial recanalization of the venous segment surrounding residual fibrosis. B: longitudinal view of the femoral vein. Normal ascending venous flow is detected in blue by color flow Doppler, and reflux is shown in red after distal compression maneuver. C: cross-sectional view of the calf muscle vein, with increased diameters and reduced compressibility. In 1, an echogenic image refers to a previous DVT, and in 2, a hypoechoic image refers to a current recurrence.

### 5.4. Valve Insufficiency

Reflux secondary to DVT, resulting from valve damage, is described in approximately 33% to 59% of the affected venous segments ( Figure 22B ). The popliteal vein is known to be the most frequently affected vein, followed by the femoral vein.^146^Reflux evaluation with distal compression maneuvers is considered abnormal when time is greater than 1000 ms in the common femoral, femoral, and popliteal veins, and equal to or greater than 500 ms in other veins.^147^

A: longitudinal view of the femoral artery (red) and femoral vein (blue). Presence of partial recanalization of the venous segment surrounding residual fibrosis. B: longitudinal view of the femoral vein. Normal ascending venous flow is detected in blue by color flow Doppler, and reflux is shown in red after distal compression maneuver. C: cross-sectional view of the calf muscle vein, with increased diameters and reduced compressibility. In 1, an echogenic image refers to a previous DVT, and in 2, a hypoechoic image refers to a current recurrence.

Some factors associated with persistent post-thrombotic changes, such as the presence of residual thrombi and/or post-phlebitic valve insufficiency, were considered predictors of PTS. However, whether such findings can be used to change a therapeutic plan remains a controversial topic in the literature.^148 , 149^

### 5.5. Discussion

#### 5.5.1. Initial Negative VUS

The US Society of Radiologists in Ultrasound has recently recommended full compliance with the VUS protocol for diagnosing DVT.^80^They also recommend that a comprehensive ultrasound examination be performed within 5 days to 1 week of an initial negative comprehensive or point-of-care ultrasound in the following situations:

- Persistent or worsening symptoms;
- Patients at high risk for DVT whose etiology of symptoms has not been elucidated;
- Technically compromised previous examinations.

#### 5.5.2. Proximal vs. Distal Thrombosis

There has been a debate regarding follow-up protocols with VUS for patients undergoing treatment for proximal DVT, ie, in the iliac, femoral, and popliteal territories. It is not clear whether patients on adequate anticoagulant medication would benefit from VUS during treatment, or whether the conclusions of a repeated examination would change the established treatment strategy. However, VUS performed at the end of treatment has been shown to be an important basis for future evaluations, being crucial for detecting which veins were recanalized and which veins still have post-thrombotic changes.^80 , 123 , 150 - 152^

Regarding isolated leg vein thrombosis, classified as axial when it involves the tibial and/or peroneal veins, and as muscular when it involves the muscular veins only, some centers do not include its study in the investigation of DVT with VUS. With this initial approach, patients should undergo a second examination on the seventh day after the onset of symptoms. The second procedure is indicated for assessing the possibility of involvement of the proximal segment, if there has been propagation of distal DVT not diagnosed in the acute phase. When the results of the second examination are normal, the possibility of thrombosis is ruled out and the patient is not anticoagulated. In such centers, the estimated risk of pulmonary embolism (PE) at the end of 3 months is 0.5% (95% CI, 0.4%-0.9%).^18 , 153 , 154^

A serial follow-up protocol with VUS is recommended given the low rate of proximal propagation of popliteal vein thrombosis, which ranges from 3 to 15%,^155 - 158^with limited benefits from anticoagulation. A meta-analysis conducted by Masuda et al.^159^reinforced the low rate of propagation. In a randomized study, Schwartz et al.^160^demonstrated no statistically significant differences between patients treated with anticoagulation for 10 days and individuals without treatment (3.7% vs. 3.8%). The propagation rate described by the authors for the popliteal vein was 1.9%.

Few studies have addressed recurrence in distal DVT. Masuda et al.^159^found no robust publications to compare patients treated with anticoagulation or followed-up with VUS. Lagerstedt et al.^161^studied patients with symptomatic distal DVT and treated with warfarin for 5 days. Patients were then allocated to warfarin maintenance for 3 months or treatment discontinuation. After 3 months, the recurrence rate of DVT was 29% and 0% in the “treated” versus “untreated” groups, respectively.

Anticoagulation treatment is chosen for cases of symptomatic distal DVT. Most authors suggest a short duration, up to 6 weeks, in low-risk patients.^160 , 162^In high-risk cases (cancer, previous DVT, involvement of two or more veins, age over 50 years, and thrombophilia), they recommend a 12-week treatment.^163^

Centers using a comprehensive VUS protocol choose to treat patients regardless of venous thrombosis being proximal or distal. The estimated risk of PE with this approach, at the end of 3 months, is also low (0.6%; 95% CI, 0.3%-0.9%).^101 , 164^

Evidence regarding post-thrombotic changes used as a parameter to guide the duration of anticoagulation is not robust. Additional research is needed in this field.^123 , 165^

A study on patients with proximal DVT treated with rivaroxaban evaluated ultrasound changes found at 1, 3, 6, and 12 months as well as recanalization rates in the affected veins.^166^Other authors^167^have highlighted the link between the presence of reflux in the popliteal vein, the permanence of fibrotic tissue, and the development of PTS. Sartori et al.^168^followed-up 172 patients with isolated leg vein thrombosis treated with enoxaparin for 6 weeks and assessed the degree of recanalization with IVUS. The examination was performed during diagnosis and at the end of treatment. Approximately half of patients (49.5%) had recanalized veins, and there was no statistical significance between recanalization and thrombus size or anatomical site involved (axial vs. muscular veins).

#### 5.5.3. Recurrent Deep Vein Thrombosis

After an initial episode of DVT, recurrence may occur in approximately 25% of patients within 5 years and account for an increased risk of PE and PTS.^169^The diagnosis of recurrent DVT is more complex because, different from the approach to the initial episode of DVT, which includes validated algorithms of pretest probability combined with the use of D-dimer and VUS,^19^such criteria are not validated for recurrence.^170^An VUS finding of non-compressibility of a previously unaffected segment can be considered diagnostic.^152^

At times, an initial thrombus will not resolve completely, which results in chronic post-thrombotic changes and may hamper the diagnosis. In this case, the diagnosis of recurrent DVT in a previously affected venous segment can be made by detecting an increase in thrombus diameter of at least 4 mm compared with a previous study. However, the accuracy of this finding is controversial,^171^and the diagnosis depends on a previous good-quality examination with an adequate number of images for comparison, together with caliber measurements in the different affected venous segments; however, this is not observed in daily clinical practice. Also, this criterion is not used in previous episodes of calf vein thrombosis, which makes patient approach even more difficult.

The follow-up of patients with recurrent DVT should follow the criteria used during the initial episode, as there is a lack of studies addressing the follow-up of this specific group. We also highlight the importance of a comprehensive study at the end of treatment, or a follow-up examination after 6 months in cases of prolonged anticoagulation, including the investigation of chronic post-thrombotic changes with an assessment of venous insufficiency with the patient in the upright position.

## 5.4. Recommendations

Based on medical literature data and guideline expert discussions, we suggest the following:

1. **Negative initial VUS:** perform a comprehensive ultrasound examination within 5 days to 1 week of an initial negative comprehensive or point-of-care ultrasound in the following situations: (a) persistent or worsening symptoms, (b) patients at high risk for DVT whose etiology of symptoms has not been elucidated, and (c) technically compromised previous examinations.
2. **Initial VUS with diagnosis of proximal DVT (iliac, femoral, and popliteal territories):** repeat comprehensive ultrasound examination towards the end of treatment to assess the degree of recanalization and presence of post-thrombotic changes. Or, at any time the patient presents with symptoms during treatment, for evaluation of recurrence or extent of the initial thrombosis.
3. **Initial VUS with diagnosis of distal DVT (tibial, peroneal, or muscular veins):** repeat ultrasound examination within 6 weeks or 12 weeks of the onset of clinical manifestations, according to the established therapeutic plan.
4. **Patients with recurrent DVT:** repeat ultrasound examination at the end of treatment, or a follow-up examination after 6 months in cases of prolonged anticoagulation, or at any time in the presence of symptoms.

## 6. Diagnosis of Pulmonary Embolism by CT Angiography, MR Angiography, and Pulmonary Angiography

### 6.1. CT angiography

The PIOPED II study^172^evaluated the role of CT pulmonary angiography combined with lower-extremity CT venography in the diagnosis of PE. Patients were clinically classified according to the probability of PE using the Wells score,^33^and pretest results were compared with posttest results. The authors demonstrated that CT angiography combined with CT venography has a positive predictive value (PPV) of 96% in patients with high clinical probability of PE, and a negative predictive value (NPV) of 97% in patients with low clinical probability. In patients with moderate clinical probability, both NPV and PPV were 92%, demonstrating an excellent case-solving capacity.

CT angiography also allows the assessment of the aorta, lung parenchyma, chest wall, and pleural space, with excellent spatial resolution, and the making of alternative diagnoses in cases of suspected PE, which may occur in up to 2/3, such as aortic dissection, pneumothorax, pneumonia, and lung cancer. Thus, the treatment for each of these conditions can be properly initiated.^173^

CT angiography is currently the method of choice for evaluating patients with suspected PE after clinical risk stratification. A negative CT angiogram in patients with low clinical probability is sufficient to rule out this diagnosis. A positive CT angiogram in patients with high clinical probability confirms the diagnosis of PE.

As CT angiography is the gold standard for the diagnosis of PE, studies that attempt to characterize prognostic factors using CT for evaluation of the RV have emerged.^174 - 177^

In a retrospective study, Schoepf et al.^177^demonstrated that an increased RV, assessed by CT angiography using the ratio between the dimensions of the RV and the LV (abnormal when > 0.9), is an independent mortality factor (OR = 5.17; 95% CI),^1 , 16 , 35 , 63^which confirms the potential use of this tool in the risk stratification of patients with acute PE.

Araoz et al.^175^selected three types of CT angiography findings (ventricular septal bowing, embolic burden, and RV/LV ratio) to assess the risk of short-term death, defined as in-hospital death or death within 30 days of CT angiography due to PE ( Figure 23 ). They concluded that ventricular septal bowing may have some clinical value as a predictor of short-term death, with high specificity (87%-88%) and low sensitivity (18%-21%). The other factors did not increase the risk of short-term death in the study sample.

**Figure 23:** – Positive CT angiography for acute PE. Signs of RV dysfunction; note increased RV dimensions (RV/LV ratio >1) and ventricular septum bowing leftward.

#### 6.1.1. Imaging Technique and Protocols

In CT pulmonary angiography, the aim is to achieve maximum pulmonary arterial opacification while keeping venous contamination and motion artifacts at a minimum, in addition to reducing radiation and contrast doses.

The main parameters to be observed are:

**a) Imaging protocols:** depend on available equipment and information such as patient’s height and weight. Ideally, they should include:

- Acquisition performed with the patient holding the breath (apnea) without exercising a Valsalva maneuver or respiratory pause;
- Use of test bolus or bolus tracking with an ROI on the pulmonary trunk to monitor the arrival of the contrast material in the pulmonary arterial circulation and to perform the examination at the appropriate time;
- Imaging from the lung base to the apex, which minimizes motion artifacts in the most important regions and reduces the artifact resulting from concentrated contrast in the superior vena cava;
- Acquisition and reconstruction of images with thin slice thickness (1 mm or less) and without gaps between images to allow multiplanar reconstructions with no loss of spatial resolution;
- Comprehensive study of all thoracic structures, which allows the assessment of possible differential diagnoses for the patient’s complaint;
- Use of lower kV, which significantly attenuates iodine and substantially reduces the radiation dose. This reduction causes an increase in image noise: the use of 100 kV instead of traditional 120 kV should be preferred, as long as the signal-to-noise ratio is kept at acceptable levels. Obese patients may need higher kV;
- Use of iterative reconstruction, which reduces radiation doses and keeps image noise at acceptable levels.

**b) Contrast material injection protocols:** depend on the available type of contrast material and venous access. Ideally, they should include:

- Large-bore antecubital vein access that allows the injection of contrast material with an injection pump at a minimum speed of 4 mL/s;
- Use of more concentrated contrast materials, such as 350 mg I/mL.

## 6.2. Dual-energy computed tomography

Dual-energy CT is able to acquire images simultaneously (or nearly) at two different energy levels.^178^The main advantages of this method for evaluation of PE are the following ( Figure 24 ):

**Figure 24:** – Dual-energy computed tomography pulmonary angiography. A: iodine-enhanced map reflecting the homogeneous distribution of contrast material within the lung parenchyma; B: MIP reconstruction in CT angiography. Dual-energy CT combines functional information with anatomical angiographic images. C: iodine-enhanced map of dual-energy CT in another patient, showing wedge-shaped perfusion defects in the left upper and lower lobes, compatible with pulmonary infarctions. Angiographic images (not shown) confirmed the diagnosis of acute PE.

- It generates an iodine-enhanced map as a by-product, which reflects at that time the distribution of contrast material across the lung parenchyma. A joint evaluation of the iodine-enhanced map with angiographic images can sensitize the detection of small thrombi in distal pulmonary branch arteries,^179 , 180^allowing monoenergetic reconstructions.
- Conversely, monoenergetic reconstructions at low energy levels (eg, 50 keV) increase iodine attenuation, but they also increase image noise. This type of reconstruction improves angiographic images in cases of suboptimal vascular opacification or allows reductions in contrast dose, eg, in patients with renal dysfunction.^179 , 180^

## 6.3. Older Generations of CT Scanners

Performing CT pulmonary angiography using older scanners can be a major challenge. However, after adjustments to imaging parameters and optimization of contrast use, using larger volumes or adjusting the injection rate may be required to compensate for a slower acquisition and to obtain good-quality diagnostic images.

## 6.4. Diagnostic Criteria

The diagnosis of acute PE with CT angiography is based on the identification of occlusive or nonocclusive thrombi within the pulmonary branch arteries. The diagnostic criteria for acute PE on CT angiography are^181^:

- Occlusive filling defect causing an increase in caliber of the affected vessel ( Figure 25A );
  **Figure 25:** – A: occlusive filling defect causing an increase in vessel caliber; B: contrast-edged, central filling defect; C: filling defect adhering to the vessel wall and forming an acute angle with it.
- Contrast-edged, central, nonocclusive filling defect ( Figure 25B );
- Nonocclusive filling defect adhering to the vessel wall and forming an acute angle with it ( Figure 25C ).

Acute PE is the main cause of filling defects in pulmonary branch arteries. Possible pitfalls and differential diagnoses include:

- Flow and/or motion artifacts;
- Incorrect anatomical interpretation;
- Reflex vasoconstriction;
- Chronic PE;
- Nonthrombotic emboli;
- Congenital anomalies (pulmonary artery agenesis and/or hypoplasia);
- Inflammatory diseases (vasculitis, fibrosing mediastinitis);
- Neoplasms (thrombi and/or tumor invasion or primary neoplasms of the pulmonary arteries).

An indirect sign of acute PE on CT is pulmonary infarction ( Figure 26 ), which can be identified even on noncontrast studies. In this situation, the study should be complemented with an angiogram to confirm the diagnosis. The typical image of pulmonary infarction is a peripheral opacity with a pleural base and central foci of attenuation that are better characterized with mediastinal window settings (vessel sign) and without air bronchograms.^12 , 182^

**Figure 26:** – Pulmonary infarction. A and B: peripheral opacity with central foci of attenuation and no air bronchograms. C: acute thrombus in the basal segmental pulmonary artery to the right lower lobe.

## 6.5. Prognostic Criteria

The changes with prognostic value that can be characterized on CT angiography are related to an acute overload of the right heart chambers. Although dilations in the superior vena cava and azygos veins and in the pulmonary trunk as well as objective measurements of RV function have been cited as prognostic markers, the most important sign of RV dysfunction is an increase in RV dimensions with an RV/LV ratio greater than 1.0 in an axial CT plane ( Figure 27 ).^181^

**Figure 27:** – CT pulmonary angiography. A: typical signs of acute PE; B: signs of overload of the right heart chambers. Note RV/LV ratio >1.0 and straightening of the interventricular septum.

## 6.6. Contraindications and Special Situations

As CT angiography is an important tool in the diagnosis of acute PE, the only absolute contraindication is a history of severe allergy to iodinated contrast material. In such cases, alternative diagnostic methods include V/Q scintigraphy, MR angiography, gadolinium-enhanced CT angiography, or serial Doppler ultrasound of the lower extremities. A history of mild-to-moderate allergic reactions can be approached with the use of premedication for desensitization.

There are other special situations that may require adaptation of the protocol or replacement of CT angiography with other diagnostic methods:

### Situation 1: young women of reproductive age or pregnant women

To avoid exposing the breast tissue to ionizing radiation, especially in patients with a normal chest radiograph, V/Q scintigraphy (or the perfusion-only study) or serial Doppler ultrasound of the lower extremities can be alternatives.

When CT angiography is used, the protocol should be optimized by ensuring adequate venous access and injection rates to achieve high diagnostic quality and avoid repetitions.

### Situation 2: chronic renal insufficiency under conservative treatment

This is not an absolute contraindication. The patient should preferably be hydrated before the examination, but cautiously to avoid congestion. In urgent cases, CT angiography can be performed even without prior preparation. V/Q scintigraphy and serial Doppler ultrasound of the lower extremities remain as alternatives.

If available, dual-energy CT scanners and monoenergetic reconstructions can be used to reduce the dose of iodinated contrast material and still ensure adequate vascular opacification.

### Situation 3: ICU patients who cannot be transported

This case requires examinations that can be performed at the bedside, such as echocardiography and Doppler ultrasound of the lower extremities.

## 6.7. MR Angiography

MR angiography is an alternative to CT angiography, allowing a direct study of the pulmonary arteries.^174 , 183^This diagnostic method was evaluated in the PIOPED III study,^183^which combined MR pulmonary angiography with lower-extremity MR venography for diagnosis.

MR angiography has some advantages over CT angiography. The main benefits are the absence of radiation and the employment of a contrast material (gadolinium) that can be used in patients with iodinated contrast allergy.^183^Also, MR angiography allows the performance of other techniques, such as lung perfusion, great vessel flow quantification, and cardiac function assessment.^174 , 183^

Currently, the indication of MR imaging for patients with renal insufficiency, especially those receiving dialysis, has been revised because of the occurrence of progressive systemic fibrosis related to the use of gadolinium.^174^

The main disadvantages of MR angiography are lower spatial resolution, higher cost, greater complexity, longer examination, reduced availability, and difficulty monitoring critically ill patients inside the machine due to the high magnetic field.^174 , 183^

The results of the PIOPED III study(183) showed that MR angiography was technically inadequate in 25% of patients. With these technically inadequate tests included, the method diagnosed PE in 57% of patients. When the technically adequate tests were analyzed separately, MR angiography had a sensitivity of 78% and a specificity of 99% for the diagnosis of PE. When MR angiography was combined with MR venography, the combination showed a sensitivity of 92%, superior to MR angiography alone, and a specificity of 96% for the diagnosis. However, the tests were technically inadequate in 52% of patients, which caused a major problem.

The PIOPED III study^183^concluded that MR angiography should only be used for the diagnosis of PE at centers of excellence in MR imaging and when other methods are contraindicated. Currently, the main indication for MR imaging is as an alternative method to CT scanning in patients with iodinated contrast allergy.^174 , 183^

## 6.8. Digital Subtraction Angiography

For a long time, digital angiography was considered the gold standard for the diagnosis of PE. Invasive nature, greater exposure to ionizing radiation, and complications of up to 0.5% of mortality, 1% of severe complications, and 5% of minor complications,^184^together with the advances of CT angiography (similar accuracy with a less invasive procedure), are determining factors for digital subtraction angiography to have virtually fallen into disuse for the diagnosis of acute PE. However, the method remains an important diagnostic tool in the evaluation of chronic PE, especially in candidates for balloon angioplasty.

## 6.9. Recommendations

- The indication of imaging methods for the diagnosis of acute PE should be based on hemodynamic status and clinical assessment of pretest probability, with the application of validated rules (Wells score, Geneva score, PERC criteria) combined with D-dimer. The aim is to avoid unnecessary use of imaging methods (grade of recommendation: I; level of evidence: A);
- CT angiography is the imaging method of choice for diagnosing acute PE. It can be replaced with V/Q scintigraphy in cases of severe iodine allergy or in special situations such as pregnant women or patients with severe renal insufficiency. MR angiography is reserved for centers of excellence in the method or for when CT angiography and V/Q scintigraphy are inappropriate or unavailable;
- The diagnosis of acute PE should be rejected in patients with low or moderate probability and negative CT angiography (grade of recommendation: I; level of evidence: A);
- The diagnosis of acute PE should be accepted in patients with moderate or high probability and CT angiography demonstrating thrombi in segmental or proximal branches (grade of recommendation: I; level of evidence: B);
- Lower-extremity CT venography is not recommended because of high exposure to ionizing radiation and should be replaced with IVUS for the evaluation of DVT (grade of recommendation: III; level of evidence: B);
- In pregnant patients, both CT angiography and V/Q scintigraphy can be used. With modern techniques, both tests cause little maternal and fetal exposure to ionizing radiation.^78 , 185^In normal radiograph cases, both CT angiography and V/Q scintigraphy can be used; however, in abnormal radiograph cases, the recommendation is to proceed with CT angiography (grade of recommendation: IIa; level of evidence: C);
- Increased dimensions of the right heart chambers, with RV/LV ratio > 1.0, are linked to 5-fold higher mortality rates due to acute PE.^186^This should be considered a tomographic criterion of RV dysfunction and a marker of poor prognosis, even in patients at clinically low risk (grade of recommendation: IIa; level of evidence: B).

Diagnostic flowcharts in hemodynamically stable and unstable patients, according to the clinical probability of PE, are shown in Charts 5 and 6 . Chart 12 shows the advantages and disadvantages of using CT pulmonary angiography in the diagnosis of PE.

**Chart 12:** – Advantages and disadvantages of CT angiography for the diagnosis of PE

CT PULMONARY ANGIOGRAPHY	
ADVANTAGES	DISADVANTAGES
Widespread availability	Ionizing radiation exposure
Excellent accuracy	Need for iodinated contrast material
Small number of inconclusive studies	Tendency to indiscriminate use because of easy access
Quick procedural time	Overdiagnosis – able to identify subsegmental thrombi, whose clinical significance is uncertain
Possibility of investigating alternative diagnoses	

## 7. Lung scintigraphy

### 7.1. Evidence

#### 7.1.1. Introduction

For practical purposes, “lung scintigraphy” is defined in this section as a single test containing different combinations of lung ventilation/perfusion scans, or even a perfusion-only study. Based on international guidelines proposed by the US Society of Nuclear Medicine and Molecular Imaging (SNMMI), the European Association of Nuclear Medicine (EANM), and the European Society of Cardiology (ESC), the main indications for lung scintigraphy are listed and discussed below.^22 , 187 , 190^

**Primary indication:** determine the probability of PE.^22 , 187 , 196^

**Secondary indications:**

- Record the degree of PE resolution in the follow-up of chronic PE;
- Quantify differential lung function before surgical resection of lung cancer;^197 - 199^
- Assess lung transplants;^200 , 201^
- Assess congenital heart or lung disease, or conditions such as cardiac shunting, pulmonary artery stenosis, and arteriovenous fistula, including their treatment.^202^
- Confirm the presence of a bronchopleural fistula.^203 - 205^
- Assess chronic parenchymal lung disorders such as cystic fibrosis;^206 , 207^
- Assess PE as a cause of pulmonary hypertension.^208^

The following findings are considered positive for PE:^189^

- Disagreement between ventilation and perfusion studies in at least one segment or two subsegments, with hypouptake present only or more extensively in perfusion compared with normal ventilation (known as a mismatch), conforming to the pulmonary vascular anatomy (wedge-shaped hypoperfusion areas with the base projecting to the lung periphery).

The following findings are considered negative for PE:

- Normal perfusion pattern conforming to the anatomic boundaries of the lungs;
- Matched pattern between ventilation and perfusion studies (defects of any size, shape, or number);
- Mismatch between ventilation and perfusion that does not follow a lobar, segmental, or subsegmental pattern.

The following findings are considered nondiagnostic for PE:

- Widespread ventilation/perfusion abnormalities not typical of specific diseases.

#### 7.1.2 Evidence Regarding Imaging Interpretation Criteria

In scintigraphic evaluation for the detection of PE, there is sufficient scientific evidence for the use of well-defined interpretive criteria, as proposed by the Prospective Investigation of Pulmonary Embolism Diagnosis (PIOPED), PIOPED II, and PISAPED studies, as well as for interpretation based on the clinical experience of the nuclear medicine or imaging physician (clinical Gestalt).

The revised PIOPED criteria proved to be more accurate than the original PIOPED criteria,^209 , 210^while the PIOPED II (which uses fewer scan categories) and PISAPED criteria resulted in a smaller number of indeterminate or inconclusive studies.^211 , 212^Both PIOPED II and PISAPED criteria had equivalent performances in terms of interpretation when chest radiography was combined with lung ventilation scintigraphy.^213^There are, however, insufficient studies replicating these results, with no double-blind multicenter comparisons between the two strategies, which jeopardizes the finding that the methods have equivalent accuracy.

Therefore, the combination of ventilation studies is recommended whenever possible, mainly because of the increased specificity of the combined study compared with radiography alone. In older adults, up to 60% of false-positive tests may result from absent ventilation, given the high prevalence of respiratory diseases.^214^

An analysis based on the experience of the nuclear medicine physician (clinical Gestalt) may provide a more accurate interpretation than studies using well-defined criteria alone. For the interpretation to be the best possible, the physician should provide an opinion based on detailed knowledge of the different objective criteria listed above.^209^

With regard to the imaging method of single-photon emission tomography (SPECT), there is a considerable body of literature supporting its use, including evidence regarding SPECT studies combined with low-dose CT, which seems to increase diagnostic accuracy.^215^The most recent EANM consensus (2019) suggests that tomographic imaging (SPECT or SPECT/CT) should always be performed routinely, leaving planar imaging for when the former is no longer possible.^188^

Although there are no robust multicenter studies comparing SPECT with planar scintigraphic imaging or even CT angiography,^216^or robust prospective studies evaluating patient outcomes with SPECT versus planar scintigraphy and CT angiography,^190^some reports have showed an increased diagnostic accuracy of SPECT and SPECT/CT. There remains some uncertainties about the interpretation of SPECT and SPECT/CT with the PIOPED and similar criteria, the best SPECT protocol for ventilation imaging, and the optimal imaging protocol (ventilation/perfusion SPECT, SPECT/CT with or without contrast enhancement, etc.).^213 , 216^However, findings of a segmental ventilation/perfusion mismatch should be regarded as positive. Findings of a single subsegmental mismatch should not be regarded as suspects for PE.^166^

Additionally, it is worth noting that, regardless of the criteria adopted for interpretation, there is a consensus that patients with low probability of PE on lung scintigraphy have a favorable prognosis^191 - 193^and that a normal perfusion study virtually excludes PE.^194 - 196^

#### 7.1.3. Diagnostic Accuracy

There is a marked difference in the diagnostic accuracy of lung scintigraphy between older studies, performed in the 1980s and 1990s, and more recent studies using updated machines and tomographic methods.^217^Older studies had a significantly lower accuracy, in addition to a rate of nondiagnostic studies nearing 65% in the original PIOPED study. The rate currently ranges from 1% to 4%, which is significantly lower than that of other imaging methods.^189 , 209 , 217^

Overall, the diagnostic accuracy of more recent studies, especially those of SPECT, is equal to or greater than those of CT angiography; in some studies, such as the PIOPED II, scintigraphy had a greater capacity to detect subsegmental PE. Therefore, there are no robust scientific data to support the superiority of CT angiography over scintigraphy.^218^

Lung scintigraphy performed as SPECT is the imaging test with the lowest rate of inconclusive results (<3%).^189 , 190^However, in practice, CT angiography remains the method of choice, mainly because of its quick procedural time and greater availability (24 hours) compared with nuclear medicine tests.^176^

The main differences in diagnostic accuracy between planar and SPECT studies are due to the greater detection of changes in subsegments and in medial segments close to the mediastinum; in the latter, SPECT can detect up to 53% more areas suggestive of PE than planar studies.^219^There was an important variability in sensitivity (Se) and specificity (Sp) values in the initial studies: Se = 67% for planar imaging and Se = 93% for SPECT in an animal model (pigs);^220^Se = 80% and Sp = 78% for planar imaging; Se = 80% and Sp = 96% for SPECT;^221^and Se = 76% and Sp = 85% for planar imaging vs. Se = 97% and Sp = 91% for SPECT.^222^Therefore, diagnostic accuracy in the initial studies generally rose from 70%-80% with planar imaging to more than 90% with SPECT.

More recent studies of over 5,000 SPECT tests have shown a negative predictive value = 97%-99%, Se = 96%-99%, and Sp = 96%-98%, which provides wide support for its use instead of planar imaging.^222 - 229^Combining SPECT with low-dose CT improves the method even further by increasing its specificity and adding the capacity of low-dose CT. This is useful in the differential diagnosis for identifying emphysema, pneumonia, and/or other parenchymal changes that cause vascular compression and may therefore explain perfusion defects.^215 , 230^( Figure 28 )

**Figure 28:** – A: series of images of planar V/Q scintigraphy with 99mTc-labeled DTPA/MAA, with the anterior, posterior, lateral, and oblique views showing normal ventilation and perfusion. B: series of images of V/Q SPECT combined with low-dose CT with 99mTc-labeled DTPA/MAA, from left to right: first column, ventilation SPECT; second column, perfusion SPECT; and third column, chest CT. The ventilation image is normal, while the perfusion image shows a perfusion defect in the lower segment of the left upper lobe (arrow) and normal CT, which characterizes PE.

More recent articles have compared SPECT with CT angiography and provided new evidence, but there remains some controversy about which method performs better.^231^Apparently, SPECT and SPECT/CT provide a more accurate diagnosis of PE in cases of pulmonary diseases with comorbidities that may hamper the diagnosis by CT angiography.^223 , 232 - 234^In a systematic review with meta-analysis, SPECT performed better than CT angiography,^231^and direct comparisons showed that SPECT is superior to CT angiography in a ROC curve analysis.^235^Importantly, SPECT has greater sensitivity in patients with chronic PE, thus being considered the reference test.^236^

In conclusion, both tests have advantages and disadvantages, with scintigraphy being superior to CT angiography in cases of concomitant diseases, chronic PE, and indeterminate CT angiography results.^223 , 231 - 234^Thus, when both modalities are available, lung scintigraphy performed as SPECT (or SPECT/CT, preferably) should be recommended, as it has no contraindications, a lower rate of indeterminate test results, and reduced exposure of the female breast to radiation (see below).^237 - 241^

Comparisons of perfusion-only scintigraphy with CT pulmonary angiography as the gold standard demonstrated a sensitivity of 86% and a specificity of 93% for perfusion imaging. When the PISAPED criteria were applied to the PIOPED II study population, perfusion-only scintigraphy had a weighted sensitivity of 82% and a weighted specificity of 96%.^213^Perfusion-only studies are recommended in patients suspected of massive PE and pregnant patients, as discussed below. Using low-dose CT instead of a ventilation study increases sensitivity compared with perfusion-only studies; however, this strategy leads to a higher rate of false-positives^215 , 23 , 242^and increased radiation exposure in pregnant women.

#### 7.1.4. Indications for Lung Scintigraphy Due to Limitations of CT Pulmonary Angiography

In addition to the previously discussed items, lung scintigraphy has been the preferred choice over other imaging methods in pregnant women because of reduced breast exposure to radiation and in some cases of limitation of contrast use on CT angiography. Such indications will be detailed below.

**a) Exposure to radiation and use during pregnancy:**

Since ventilation studies and chest radiographs are commonly normal in young individuals, a usual recommendation is skipping this portion of the study in those known to be at risk for the effects of radiation, such as women in the first trimester of pregnancy, because of the likely greater vulnerability of the fetus to the effects of radiation.^189 , 190^

In pregnant women, a protocol including a perfusion-only study and reduced radioactivity is initially recommended (see section on imaging techniques). A normal perfusion study excludes PE, and the ventilation study should only be performed in exceptional cases or cases of uncertainty about a positive result the day after the perfusion scan.^243^This ensures reduced radiation exposure compared with CT angiography and no exposure to iodinated contrast material.^189^

The critical maternal organ to be protected in this case is the breast, which absorbs radiation doses between 8.6 to 44 mSv on CT angiography^237 , 238 , 244^and less than 1 mSv on perfusion-only scintigraphy when not combined with tomography or ventilation studies.^237^Fetal doses seem to be low and similar in both methods.

**b) Other clinical limitations of CT angiography:**

Because of the intravenous use of iodinated contrast material, CT angiography might not be performed in a large number of critically ill patients, such as those with renal insufficiency, those who have recently had a myocardial infarction, those requiring ventilatory support, and those with a history of iodinated contrast allergy/anaphylaxis. In such cases (in addition to those mentioned in item A and in the section on diagnostic accuracy) and in rare cases of inability to perform the test, lung scintigraphy should always be indicated, as it has no contraindications, no related complications, and a low rate of inconclusive test results.^189 , 190 , 223^

#### 7.1.5. Protocol

**1. Ventilation study**

**a. Radiopharmaceuticals:**

**i. Inert gases:**

**1.** ^133^ **Xe:**

- This is historically the agent used in ventilation studies.^187 , 245 , 246^
- It has a half-life of 5 days and a (low) energy of 81 keV.^187 , 209^
- In the PIOPED I study, the single-breath technique was the most used.^187 , 209^
- ^133^Xe is inhaled during the first 20 seconds, and one image should be obtained from the posterior view.^187 , 247^
- This gas is not available in Brazil.

**2.** ^81m^ **Kr:**

- This is produced from a high-cost generator of rubidium (^81^Ru).^187 , 248^
- It has the ideal gamma energy (193 keV) and a half-life of 13 seconds.^187 , 249^When a patient is breathing air with^81m^Kr at a normal respiratory rate, the regional alveolar concentration of this gas is at steady state, proportional to regional ventilation.^187^During steady-state^81m^Kr inhalation, multiple planar imaging or SPECT imaging are feasible. Very recently, the combination of SPECT with low-dose CT has been described.^187 , 215^
- ^81m^Kr is a true gas that does not cause artifacts due to central airway deposition. An advantage is that ventilation and perfusion can be imaged simultaneously, as the gamma energy of^81m^Kr is greater than that of^99m^Tc (used as a perfusion marker).^187^
- Low radiation exposure makes^81m^Kr the best choice for children.
- This gas is not available in Brazil.

**ii. Radiolabeled aerosols:**

Given the unavailability of^133^Xe and^81m^Kr in Brazil for ventilation studies, radioaerosols are generally used.
2. An aerosol is a relatively time-stable, two-phase system consisting of particles suspended in air. The radiolabeled particles may be liquid, solid, or a combination of the two. The percentage of particles remaining in the lung after inhalation (deposition fraction) depends on the aerodynamic properties of the particles, particularly on their size. The deposition fraction is up to 50%, with ultrafine nanoparticles (diameter, 0.02 ), which are deposited predominantly in the alveolar region by diffusion.^187 , 250^
The most commonly used radiolabeled aerosol is^99m^Tc-DTPA, which is cleared from the alveolar region by transepithelial diffusion. It has a biological half-life of 80 minutes in healthy nonsmokers, 45 minutes in healthy passive smokers, and 24 minutes in healthy active smokers.^251^
Another radiolabeled aerosol available is Technegas, which consists of extremely small^99m^Tc-labeled solid graphite particles generated at high temperatures.^252 , 253^These particles have a diameter of about 0.005 to 0.2 micrometers,^254^are hydrophobic, and tend to aggregate, which is why they should be used within 10 minutes of being generated. They are suspended in argon gas (therefore having the property of gas). Studies using^99m^Tc-Technegas and^81m^Kr have provided comparable results.^255 - 259^However, the availability of Technegas equipment in Brazil is low, especially because of its high cost.

**b. Imaging protocols:**

i. As PE is a life-threatening condition, the recommendation is that the diagnosis be made as soon as possible, usually with 1-day ventilation/perfusion protocols. Lung ventilation studies, which will be used for comparison with the corresponding perfusion study, should be performed before perfusion imaging, requiring very low radioactivity. Sequential perfusion imaging with low-to-moderate radioactivity produces high-quality comparative images, with the diagnosis being made in a short time.

ii. Whenever possible, tomographic imaging should be performed as follows:

1. Ventilation with the appropriate radiolabeled aerosol, patient in the supine position, using a 1 to 4 dose ratio between ventilation and perfusion, respectively (usually 25 to 30 MBq for ventilation studies).
2. A large field-of-view, dual- or triple-head gamma camera and a general-purpose collimator should be used. At least 128 images will be obtained in a 64 x 64 matrix and 10 seconds per image.
3. Perfusion images should be obtained immediately after ventilation images. To avoid patient movement, peripheral venous access for perfusion imaging should be placed before the start of the ventilation study.

**2. Perfusion study**

**a. Radiopharmaceutical:**

i.^99m^Tc-labeled macroaggregated albumin (^99m^Tc-MAA) is used in perfusion studies.

**b. Imaging protocols:**

- Patient should be in the supine position, and venous access should have been previously obtained (prior to ventilation imaging, so that the patient does not move);
- Venous access is used to administer activity between 100 and 120 MBq^99m^Tc-MAA (number of MAA particles is approximately 200,000 to 700,000 in specific clinical situations, such as pulmonary hypertension and right-to-left shunting. The number of particles should be reduced to 100,000 to 150,000).
- In the same gamma camera used for ventilation imaging, without any patient movement, perfusion images should be obtained as follows:
- 128 tomographic images (SPECT) in a 64 x 64 matrix and 5 seconds per image.^260^

**3. Perfusion-only study:** in pregnant patients and those suspected of massive PE, a perfusion-only study is indicated.

#### 7.1.6. Image Reconstruction

Images should be iteratively reconstructed with ordered-subsets expectation maximization (OSEM), according to a suggested protocol with 8 subsets and 2 iterations.^219 , 260 , 261^

#### 7.1.7. Interpretation

**a)** In V/Q SPECT and planar V/Q scintigraphy, images should be interpreted based on:

- Basic criteria for evaluating imaging studies;
- Knowledge and experience of the interpreting physician (clinical Gestalt);
- Pretest probability according to holistic interpretation principles.

**b)** For them to be clinically relevant, lung scintigrams should be described as positive or negative for the presence of PE (PE: YES or NO), not based on probability categories.^84^

**c) Basic criteria for interpreting V/Q scintigrams:**

**i. Reports of absence of PE are based on:**

1. **i.1** . Normal lung perfusion;
2. **i.2.** Matched or reversed-mismatched V/Q defects (presence of ventilation change, no perfusion superposition) of any size, shape, or number, in the absence of true mismatch;
3. **i.3** . Mismatch with no lobar, segmental, or subsegmental pattern.

**ii. Reports of presence of PE are based on:**

1. **ii.1** . V/P mismatch of at least 1 segment or 1 subsegment conforming to the lung perfusion anatomy.
2. **iii. A study is nondiagnostic when:**
3. **iii.1.** Multiple V/P abnormalities not typical of specific diseases are observed.

#### 7.1.8. Chronic Pulmonary Embolism

Chronic PE is a medical condition different from acute PE. It is an insidious, progressive disease that has no treatment and a poor prognosis.^262 , 263^Mortality results from pulmonary hypertension, right heart failure, and arrhythmias. V/Q scintigraphy is generally used as an auxiliary tool in the diagnosis of chronic thromboembolic pulmonary hypertension,^208 , 264^with greater sensitivity and specificity than multidetector CT.^265^

#### 7.1.9. Additional Diagnostic Findings

Ventilation and perfusion scintigraphy with tomographic imaging (V/Q SPECT) has been able to demonstrate the presence of clinical situations other than PE, such as COPD, left heart failure, and pneumonia. In 39% of patients who undergo the diagnostic procedure and do not have PE, and in 22% of those with PE, some of the following additional findings will be present.^223^

**a) COPD:** characterized by matched ventilation/perfusion defects. Ventilation defects are frequently more pronounced than perfusion defects, a phenomenon known as reverse mismatch.^266 , 267^A significant correlation between the degree of ventilation abnormalities detected on scintigraphic studies and the results of pulmonary function tests has been described.^268^PE is frequent in patients with COPD,^269^accounting for about 10% of deaths in those with stable COPD.^270^

**b) Left heart failure:** perfusion is redistributed to the upper lung regions.^271 , 272^Ventilation redistribution is less extensive than perfusion redistribution. A mismatch can be observed in the dorsal regions, with a diffuse nonsegmental pattern, and should not be interpreted as PE.

**c) Pneumonia:** matched ventilation/perfusion defects are observed, and the former are usually larger, causing a reverse mismatch.^273 , 274^A finding suggestive of pneumonia is the stripe sign, which refers to the maintenance of perfusion in the pleural surface adjacent to a central area of matched defect.^275^The stripe sign pattern is best detected on tomographic studies.

#### 7.1.10. Pitfalls in the Interpretation of V/Q Studies

As is the case with any diagnostic method, V/Q scintigraphy requires the nuclear medicine physician reporting the study to be aware of possible errors inherent to the test, such as the following:

a) Technical artifacts may arise from preinjection handling of^99m^Tc-MAA. Drawing blood from a patient into a syringe containing the^99m^Tc-MAA solution may cause aggregation of particles, creating the so-called hot spots. A similar defect may arise from failure to resuspend^99m^Tc-MAA particles prior to intravenous administration.

b) Planar imaging may underestimate the presence and/or extent of perfusion abnormalities due to superposition of normally perfused areas. This artifact is eliminated by scintigraphic imaging with tomographic views (SPECT).

c) Whenever possible, the use of Technegas is preferred over liquid aerosols in patients with COPD. In rare patients with emphysema, Technegas particles are trapped in bullae, leading to a pattern that may be mistaken for a mismatch.^222^

d) Mismatched perfusion defects with no clear segmental pattern may be seen in older patients in cases of partially resolved PE, but not in cases of acute PE. Also, mismatched perfusion defects are observed in other lung conditions, such as lung cancer, mediastinal lymphadenopathy, actinic sequelae, pneumonitis, fibrosis, and heart failure.

e) Ventilation and perfusion scintigraphy may fail to detect thromboembolism that causes only partial vascular obstruction with few hemodynamic effects. This problem has low clinical significance. A possible explanation is that nonocclusive emboli are usually associated with other signs of PE in other regions, leading to a correct diagnosis. Nonetheless, when the presence of partial occlusion is identified (segmental perfusion defect found in the presence of normal ventilation), the study should be reported as positive for PE.

f) Overall, the complete absence of unilateral perfusion in the presence of normal ipsilateral ventilation and without any other mismatch in the contralateral lung is not due to PE.^276 , 277^In such cases, chest CT scans usually reveal the presence of other conditions, such as tumors or other mediastinal processes, congenital pulmonary vascular abnormalities, or aortic aneurysm.

#### 7.1.11. The future of Nuclear Medicine Evaluation of PE

In the 1970s,^68^Ga, one of the first isotopes used in lung perfusion imaging, was introduced as the future of nuclear medicine pulmonary evaluation. As it is a positron-emitting isotope,^68^Ga requires a PET system and is used as^68^Ga-labeled microspheres to assess perfusion. Ventilation, in turn, is assessed with a similar technique to that of Technegas, using^68^Ga-labeled particles called Galligas^278^( Figure 29 ).

**Figure 29:** – On the left, a series of images showing a normal V/Q SPECT/CT scintigram with 99m Tc-labeled Technegas/MAA; from top to bottom: low-dose CT; ventilation SPECT; fused ventilation/CT; perfusion SPECT; and fused perfusion/CT. On the right, a series of images showing a normal V/Q PET/CT scan with 68 Ga-labeled Galligas/MAA; from top to bottom: sagittal views of fused ventilation/CT (left) and perfusion/CT (right). Adapted from Le Roux PY et al. 279 and Bailey DL et al. 280

The advantages of transitioning from V/Q SPECT to PET/CT include superior imaging characteristics, such as higher sensitivity of the ring detector (covering 360 degrees simultaneously), better count statistics, higher spatial and temporal resolution, and reduced acquisition time (approximately 10 min, decreasing to 5 min with new digital PET/CT scans). Other advantages are the commercial availability of respiratory-gated acquisition systems that reduce artifacts, especially at lung bases; the possibility of multiple ventilation studies with or without bronchodilator on the same day owing to the short half-life of^68^Ga; and important characteristics such as being a simple and noninvasive test without contraindications or side effects related to the use of contrast material (eg, allergy and renal dysfunction). At the technical level, the transition does not imply major difficulties or investments because the radiopharmacy requirements remain relatively simple with the use of existing Technegas equipment. The only difference is the modified MAA synthesis and its consequent labeling with^68^Ga (whose generators are increasingly available, enabling widespread adoption) instead of^99m^Tc, with a radiation dose very similar to that of conventional V/Q SPECT/CT scans^108^( Figure 30 ).

**Figure 30:** – On the left, a series of images showing a normal V/Q SPECT/CT scintigram with 99m Tc-labeled Technegas/MAA; from top to bottom: low-dose CT; ventilation SPECT; fused ventilation/CT; perfusion SPECT; and fused perfusion/CT. On the right, a series of images showing a V/Q PET/CT scan with 68 Ga-labeled Galligas/MAA; from top to bottom: sagittal views of fused ventilation/CT (left) and perfusion/CT (right); matched defect, fused PET/CT perfusion image showing a perfusion defect (arrow); and a CT image showing a tumor obstructing the bronchus, corresponding to the ventilation/perfusion defect on PET imaging.

Adapted from Le Roux PY et al.^279^and Bailey DL et al.^280^

In 2018, Le Roux et al.^281^proposed a protocol for systematic review and meta-analysis of diagnostic accuracy and clinical outcome of patients evaluated with V/Q SPECT, whose results are still to be published and will certainly provide more evidence for the adoption of this technique.

#### 7.1.12. Clinical Algorithm for the Investigation of Patients with Suspected PE

Assessing the clinical probability of PE helps physicians choose the most appropriate objective test to diagnose or exclude the diagnosis ( Chart 6 ). Measurement of D-dimer (a degradation product of cross-linked fibrin clot) is widely used in the investigation of patients with suspected venous thromboembolism (VTE). The quantitative D-dimer assay, based on a rapid ELISA test, has high sensitivity (close to 95%) for VTE.^22 , 23^However, the assay has low specificity (40%) because D-dimer levels may be increased in several conditions other than VTE, such as in acute myocardial infarction, stroke, inflammation, active cancer, and pregnancy. Specificity also decreases with age, and it can be as low as 10% in older adults.^120 , 214^Consequently, a negative quantitative D-dimer test has a high negative predictive value for VTE. The results of the studies reveal that the risk of development of PE in patients with low clinical probability who are not treated after a negative D-dimer test is <1% within three months of the initial evaluation.^282^Conversely, because of its low predictive value, a positive quantitative D-dimer test does not modify the pretest (clinical) probability and is therefore clinically useless. Recent evidence, however, suggests that very high D-dimer levels are associated with a 4-fold increase in the probability of PE,^30^which is important for assessing the burden of thromboembolic disease and may have prognostic significance.^27 , 29^

Diagnostic strategies will depend on the patient’s hemodynamic stability, as follows:

**a) Stable patients:**

Based on these considerations, when the clinical probability of PE is low and the quantitative D-dimer test is negative, the diagnosis is unlikely and additional investigations are not needed ( Chart 5 ). When the clinical probability of PE is low and the quantitative D-dimer test is positive, additional investigations may be needed for a range of diagnoses, including PE, especially when D-dimer levels are markedly high. When the clinical probability is other than low, it seems more reasonable to skip the D-dimer test and refer the patient directly to the appropriate imaging technique ( Chart 5 ). It can be V/Q SPECT or CT angiography, depending on local availability, medical knowledge, and the patient’s clinical status. V/Q SPECT has virtually no contraindications and produces a substantially lower radiation burden than CT angiography.^22^

**b) Hemodynamically unstable patients:**

When the patient presents with severe hypotension or cardiogenic shock ( Chart 6 ), transthoracic echocardiography remains the first-line imaging technique, as it detects right heart dilatation and hypokinesia. Embolism is rarely seen within the right heart chambers or the main pulmonary artery. Lung perfusion scintigraphy is an alternative to CT angiography, as it quickly shows several segmental or lobar perfusion defects typical of acute PE.^120^When acute thoracic aortic dissection is suspected due to chest pain, CT angiography allows this differential diagnosis to be evaluated.

Given the need for expediting the diagnosis and treatment of these patients, the strategy employed in a specific health facility should be adapted to each clinical situation and local context. When the initial examination suggests a massive PE, other actions should be adapted to the clinical situation, and antithrombotic therapy may be administered.

#### 7.1.13. Diagnostic Algorithm

When suspected, PE should be confirmed or refuted to avoid the risk of over- and undertreatment, which requires imaging tests. Only the optimal techniques, ie, CT angiography and V/Q SPECT, are recommended. The choice of imaging modality will depend on availability.^120^Although CT angiography is more readily available, it is contraindicated in a substantial number of patients, as shown in the PIOPED II study.^217^Currently, V/Q SPECT is rarely available 24 hours a day, 7 days a week. Consequently, the two tests should be ready for use at least in tertiary care centers, as both are crucial for appropriate diagnostic algorithms of PE. In each center, the diagnostic algorithm used for PE should be based on local conditions and particularly on the availability of V/Q SPECT and CT angiography.

V/Q SPECT, when available, has considerable advantages over other imaging techniques for the diagnosis of PE, such as high sensitivity and specificity, lower predictable radiation burden, and suitability to the follow-up of patients with PE and its natural history.

Diagnostic flowcharts for hemodynamically stable and unstable patients, according to the clinical probability of PE, are shown in Charts 5 and 6 . Chart 13 shows the advantages and disadvantages of lung scintigraphy for the diagnosis of PE.

**Chart 13:** – Advantages and disadvantages of lung scintigraphy for the diagnosis of PE

LUNG SCINTIGRAPHY	
ADVANTAGES	DISADVANTAGES
Low radiation exposure	Limited availability
Can be used in pregnant women	Does not detect the occluded artery with absolute certainty
Radiopharmaceutical does not impair renal function	
High accuracy	
Procedure covered by SUS and by the ANS health procedure list	
Allows the physician to assess chronic PE	
Allows the physician to assess resolution of acute PE	

#### 7.1.14. Conclusions

- V/Q scintigraphy is strongly recommended for the diagnosis of PE, with high accuracy even in the presence of COPD and pneumonia;
- Technegas is superior to DTPA in patients with COPD;
- When available,^81m^Kr is advantageous;
- The radiation dose should be reduced as much as possible, not affecting image quality (ALARA principle). Overall, 30 MBq^99m^Tc-labeled aerosol should be used for ventilation and 100-120 MBq^99m^Tc-labeled MAA for perfusion;
- In pregnant patients, the perfusion-only study is recommended;
- The interpretation should be holistic; the probabilistic interpretation is obsolete;
- A key criterion for the diagnosis of PE is the presence of mismatch in more than 1 subsegment.
